# Anion-Exchange-Membrane Electrolysis with Alkali-Free Water Feed

**DOI:** 10.1021/acs.chemrev.4c00466

**Published:** 2025-08-01

**Authors:** Mohsin Muhyuddin, Carlo Santoro, Luigi Osmieri, Valerio C.A. Ficca, Ariel Friedman, Karam Yassin, Gioele Pagot, Enrico Negro, Anastasiia Konovalova, Grace Lindquist, Liam Twight, Minkyoung Kwak, Enrico Berretti, Vito Di Noto, Frédéric Jaouen, Lior Elbaz, Dario R. Dekel, Piercarlo Mustarelli, Shannon W. Boettcher, Alessandro Lavacchi, Plamen Atanassov

**Affiliations:** † Electrocatalysis and Bioelectrocatalysis Laboratory (EBLab), Department of Materials Science, 9305 University of Milano-Bicocca, U5, Via Roberto Cozzi 55, 20125 Milan (MI), Italy; ‡ Materials Physics and Applications Division, 5112 Los Alamos National Laboratory, Los Alamos, New Mexico 87545, United States; § ENEA Casaccia Research Center, Via Anguillarese 301, Rome 00123, Italy; ∥ Center for Clean Energy Engineering (C2E2), University of Connecticut, 44 Weaver Road, Storrs, Connecticut 06269, United States; ⊥ The Wolfson Department of Chemical Engineering, 26747 Technion − Israel Institute of Technology, Haifa 3200003, Israel; # The Nancy & Stephen Grand Technion Energy Program (GTEP), Technion − Israel Institute of Technology, Haifa 3200003, Israel; 7 Section of Chemistry for the Technology (ChemTech), Department of Industrial Engineering, University of Padova, Via Marzolo 9, I-35131 Padova (PD), Italy; 8 Department of Energy Conversion and Storage, 5205 Technical University of Denmark, Fysikvej, Building 310, Lyngby 2800, Denmark; 9 Department of Chemistry and Biochemistry, 3265 University of Oregon, Eugene, Oregon 97403, United States; 10 201843 Istituto di Chimica Dei Composti OrganoMetallici (ICCOM), Consiglio Nazionale Delle Ricerche (CNR), Via Madonna Del Piano 10, 50019 Sesto Fiorentino, Firenze, Italy; 11 ICGM, Univ. Montpellier, CNRS, ENSCM, 34095 Montpellier, France; 12 Department of Chemistry and the Institute of Nanotechnology and Advanced Materials, Bar-Ilan University, Ramat-Gan 5290002, Israel; 13 Israel National Institute of Energy Storage (INIES), Technion − Israel Institute of Technology, Haifa 3200003, Israel; 14 Department of Chemical & Biomolecular Engineering and Department of Chemistry, University of California, Berkeley, Berkeley, California 94720, United States; 16 Energy Storage and Distributed Resources Division, Lawrence Berkeley National Laboratory, Berkeley, California 94720, United States; 15 Department of Chemical and Biomolecular Engineering, University of California, Irvine, Irvine, California 92697, United States

## Abstract

Hydrogen is a green and sustainable energy vector that can facilitate the large-scale integration of intermittent renewable energy, renewable fuels for heavy transport, and deep decarbonization of hard-to-abate industries. Anion-exchange-membrane water electrolyzers (AEM-WEs) have several achieved or expected competitive advantages over other electrolysis technologies, including the use of precious metal-free electrocatalysts at both electrodes, fluorine-free hydrocarbon-based ionomeric membranes and bipolar plates based on inexpensive materials. Contrasting the analogous proton-exchange-membrane system (PEM-WE), where pure water is circulated (no support electrolyte), the current generation of AEM-WEs necessitates the circulation of a dilute aqueous alkaline electrolyte for reaching high energy efficiency and durability. For several reasons, including but not limited to lower cost of balance-of-plant, lower operating cost and improved device’s lifetime, achieving high cell efficiency and performance using an alkali-free water feed is highly desirable. In this review, we develop and build a foundational understanding of AEM-WEs operating with pure water, as well as discuss the effects of operating with natural water feeds like seawater. After a discussion of the possible advantages of pure-water-fed AEM-WEs, we cover the thermodynamic and kinetic processes involved in AEM-WE, followed by a detailed review of materials and components and their integration in the device. We highlight the influence of electrolyte composition and alkali/electrolyte-free feed on the membrane-electrode assembly, ionomers, electrocatalysts, porous transport layer, bipolar plates and operating configuration. We provide evidence for how the pure water feed engenders several issues related to the degradation of device components and propose mitigation strategies.

## Graphical abstract

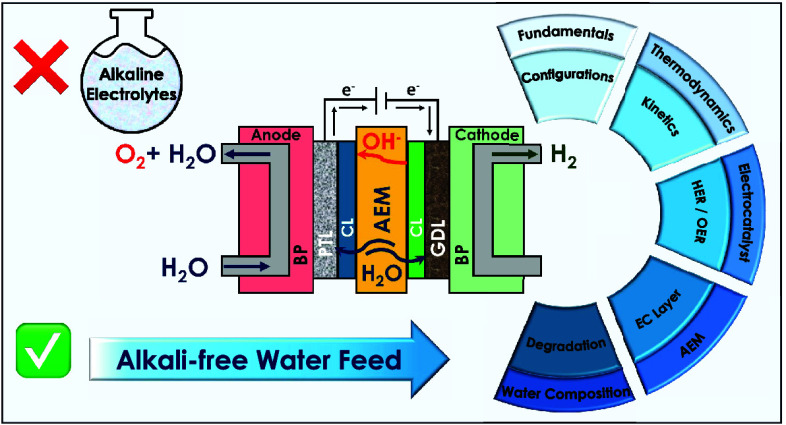

## 1 Introduction

Water availability and quality are critical factors in the sustainable production of hydrogen via electrolysis. While water is abundant, its use in electrolyzer systems presents several challenges, particularly in terms of purification, treatment, and overall operational cost. Traditional water electrolysis methods, such as alkaline water electrolysis (A-WE) and proton exchange membrane water electrolysis (PEM-WE), often require high-purity deionized water or the addition of strong alkaline electrolytes like potassium hydroxide (KOH) to enhance ionic conductivity. While effective, these approaches introduce issues related to system complexity, material degradation, and environmental impact due to the corrosive nature of alkaline solutions and the reliance on costly, often scarce materials.

Hydrogen is a critical element to achieve the decarbonization goal. The International Energy Agency (IEA) underscores the urgency of moving beyond fossil fuels, urging the achievement of a fully decarbonized society by 2050.^1^ Aligning with this vision, both the EU and the US have incorporated this objective into their decarbonization agendas. This shift mandates a significant integration of renewable energy sources across all economic sectors. However, the intermittent nature of renewable energy poses various challenges. Variability in supply, intrinsic to natural weather patterns, necessitates advanced energy storage solutions for both short (days to weeks) and longer (seasonal) timeframes that will complement battery systems and other energy storage technologies.^2–4^

Hydrogen is a promising fuel for long-haul transportation via trucks, ships, and planes, both for fuel cells and internal combustion engines, while hydrogen vehicles promise enhanced urban air quality. Overall, hydrogen can strengthen energy security as a fuel that can be produced domestically.^5^ In the chemical sector, ammonia and fertilizer manufacturing require new sources of hydrogen not derived from fossil fuels. For industrial processes using high temperatures, as in cement, iron, and steel production, hydrogen may play another important role as a reducing agent and for the decarbonization of such processes. Whether it is balancing renewable energy overproduction during certain periods, direct conversion to electricity via fuel cells, or being utilized as a chemical reactant, hydrogen’s adaptability and broad applications are evident.

Traditionally hard-to-abate sectors are significant sources of greenhouse gas emissions and must be addressed for a successful climate strategy. In a 2019 assessment, emissions were distributed as follows: electricity and heat production (15.83 billion tons CO_2_-equivalent), transportation (8.43 billion tons), industry processes (3.06 billion tons), and aviation and shipping (1.31 billion tons).^6^ While “green hydrogen” produced using renewable-sourced electricity is on the horizon, a 2021 report from the International Renewable Energy Agency (IRENA) showed that ∼ 96% of hydrogen produced today originates from fossil fuels via steam reforming of natural gas (47%), coal (27%), and oil (22%). Only 4% of H_2_ was produced through water electrolysis.^7^ To ensure hydrogen’s role in decarbonization, its production must be free from greenhouse gas emissions. Consequently, only hydrogen production powered entirely by renewable energy can be classified as environmentally sustainable.^8^

Green hydrogen is produced by water electrolysis, using various technologies with different degrees of maturity. Some processes remain in the proof-of-concept phase – technology readiness level (TRL) 1, for instance, photoelectrochemical hydrogen production – others have progressed to lab or pilot-scale development (TRL 4–7), such as anion exchange membrane water electrolyzers, AEM-WE, discussed here. Some are navigating through the prototyping and scaling process, while others have matured, finding integration into industrial settings (TRL 8–9), notable examples being PEM-WE, A-WE, and solid oxide electrolyzer cells (SOEC).^9^

Desired properties of water electrolyzer systems include:

- *High durability*: Ensuring components maintain consistent functionality over a prolonged duration
- *High hydrogen production rates and electrical efficiency*: High rates (current) over a small footprint
- *Low capital expenditure*: preferential use of abundant and cheap materials, scalable synthesis of functional materials
- *Ability to electrochemically pressurize hydrogen*: Pressurized H_2_ is needed for transportation applications and reducing the reliance on external compressors brings energy savings.

Water Electrolyzer systems are broadly classified into high-temperature (typically > 100 °C, where water is vapor) and low-temperature (slightly <100 °C, where water is liquid) systems. SOECs operate typically within the 500 to 900 °C range and use a solid ceramic separator.^10,11^ While maintaining elevated temperatures causes challenges when integrating with intermittent renewable energy sources, SOECs can have exceptionally high efficiency due to fast electrode kinetics at high temperatures and do not need precious metals.^12^ SOECs thus tend to integrate well with nuclear energy, where consistent baseload electricity and residual heat sidestep the challenges of intermittency.

Low-temperature WEs can be subdivided into three main types, in the chronological order of invention: A-WE, PEM-WE, and AEM-WE. Their principles of operation are schemed in Figure 1.

**Figure 1:**
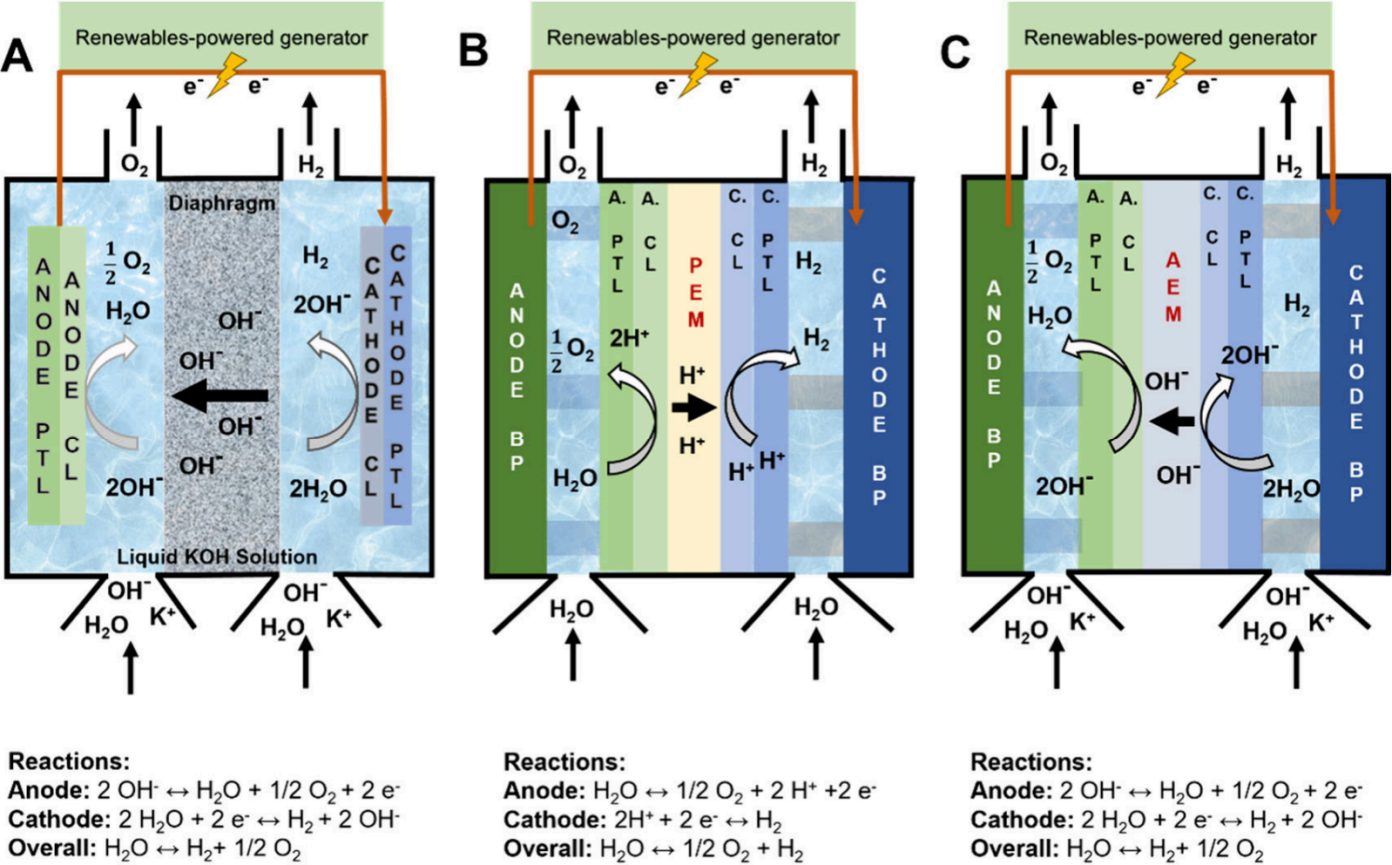
Schematic of low-temperature WEs. (A) A-WE, (B) PEM-WE and (C) AEM-WE. The PTL is the porous transport layer, usually a metal- or carbon-based porous layer that supports the catalytic layer (CL), which is the electrocatalytically active region of the device. The CL is composed of electrocatalyst powder or self-supported electrocatalyst surface, ionomer solid electrolyte, porosity for water and gas transport, and also for ionic conduction when a support electrolyte is used, i.e., for A-WE and some operating configurations of AEM-WE.

A-WE is the oldest,^13^ most mature (TRL 8–9) and most widely manufactured electrolyzer technology.^14,15^ A-WE consists of anode and cathode electrodes separated by a diaphragm. Both electrodes are immersed in a water-based, highly concentrated (5–7 M) KOH electrolyte that is recirculated, guaranteeing continuous operations. The diaphragm is a porous material generally made of zirconium oxide stabilized with polyphenylene sulfide.^16–18^ The diaphragm is used as a passive separator, and the ionic transport is provided by the KOH electrolyte that fills its pores, typically several hundred nanometers in dimension. A-WEs can operate in the temperature range of 70–90 °C with an operating pressure of 1–30 bar.^19–22^

The second type of low-temperature WE is the PEM-WE, which also has a TRL of 8–9.^23^ PEM-WEs consist of an anode and cathode mounted on each side of a PEM forming the so-called membrane-electrode-assembly (MEA). The membrane is a proton-conducting polymeric material, which also electronically separates the anode from the cathode and strongly mitigates the cross-diffusion of H_2_ and O_2_. The state-of-the-art PEM is currently based on a polymer backbone of polytetrafluoroethylene (PTFE) with fluorinated vinyl ether lateral chains ending with sulfonic groups. The PEM structure consists of two main regions: 1) the hydrophobic PTFE backbone; 2) the hydrophilic end chains including sulfonic acid groups.^24,25^ PEM-WEs are fed with deionized water (DI) in the temperature range of 50–80 °C with an operating pressure of up to 70 bar.^26^

The third and most recent low-temperature WE is the AEM-WE.^19^ AEM-WE shares the same architecture as PEM-WE with the MEA based on sandwiched anode|membrane|cathode. AEMs are composed of a polymeric backbone functionalized with cationic groups that allow anions to move through the membrane. As PEM, here the AEM acts as a physical, nonporous separator. AEM-WE usually operates with supporting electrolytes, mainly KOH, potassium carbonate (K_2_CO_3_) or sodium carbonate (NaHCO_3_), which, however, are more diluted compared to the A-WE ones. AEM-WEs operate in the range of temperature of 40–60 °C with an operating pressure of up to 35 bar.^20^

AEM-WE is still in the developing phase and the wide range of the technology is due to the drastically different readiness levels of electrolyte-fed systems (TRL 6–7) and pure-water-fed systems (TRL ≤ 4).^27^ Pure water-fed AEM-WEs, in principle, offer significant advantages to PEM-WE and A-WE by: (i) operating efficiently and durably without critical raw materials (CRM), containing the costs and improving the sustainability due to the reduction of CO_2_ emissions caused by the energy-intensive mining of rare metals like iridium; (ii) operating without supporting electrolyte, enabling the use of inexpensive stainless steel components in the balance of plant (BoP) and simplifying BoP design, substantially reducing capital expenses, (iii) operating expenditure decrease avoiding the usage of KOH or other supporting electrolytes that have a cost and their production is energy intensive,^16^ (iv) operating with polymeric membranes that do not contain potentially harmful fluorinated polymers; (v) pressurizing hydrogen internally across the membrane using voltage (lowering the operation costs); and vi) efficiently maintain separated hydrogen and oxygen by using a polymeric membrane (avoiding safety issues of A-WE, especially under intermittent operation). If AEM-WE operating with a pure-water feed can match the performance metrics reached to date with electrolyte feeds, it will open the door to a competitive technology that has the potential to overcome the issues of both PEM-WEs and A-WEs while keeping their advantages. Thereby, it would offer a solution to produce green H_2_ at a competitive cost and in a sustainable way. Despite the increased interest in AEM-WE research in the past decade, progress with pure water-fed AEM-WEs has lagged behind, as most studies have hitherto been conducted with supporting electrolytes, as reported in Figure 2, where the number of publications related to devices using AEM, AEM-WE operating with supporting electrolytes and water-fed are shown.

**Figure 2:**
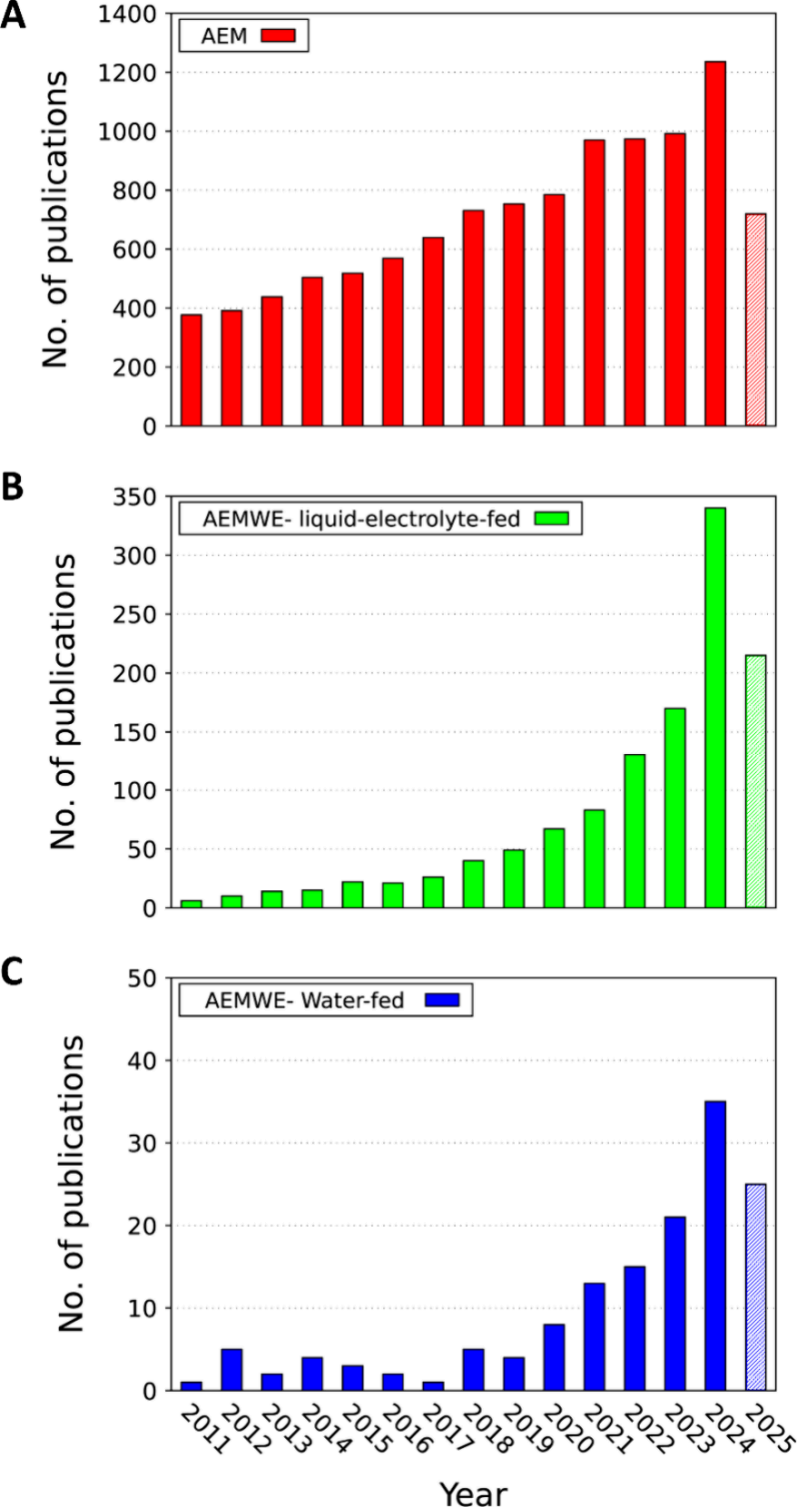
Annual number of publications in the field of AEMs (from Web of Science, accessed June 25, 2025). Search terms: “Anion exchange membrane”, “electrolyte-fed AEM-WE”, “water” and “electrolysis”.

This review focuses on AEM-WE operating without the use of supporting electrolytes (e.g., KOH), using nominally pure water feed. We also discuss progress and challenges feeding abundant natural water, like seawater and the effect of elements, anions, cations, and various molecules is also discussed. In AEM-WE, KOH is typically added to the water as an electrolyte to enhance the ionic conductivity that significantly boosts the performance; however, the operational durability still remains under question due to the stability limitations of the anion exchange polymer under harsh anodic conditions.^28^ Adding KOH even at concentrations as low as 1 M creates a very corrosive environment (especially at the typical operating temperature of 70–90 °C) that necessitates using expensive components in the system. Seawater, despite being a practically “free” electrolyte with no water purification cost, is also quite corrosive, particularly as chloride can be converted during operation to highly oxidizing products at the anode, affecting the durability of the AEM-WE. Cells operating with pure water will have lower operational costs if the underlying stability and efficiency issues can be solved. This would be beneficial, even though the feedwater must be demineralized and/or purified, because these processes are not energy-intensive compared to the energy required to produce hydrogen. Currently, the industry is commercializing AEM-WEs operating with dilute KOH because the cost and performance are reasonably compelling. However, the interest remains extremely high in AEM-WE operating with pure water without supporting electrolytes.

This review is divided into seven main sections. Following the introduction (section 1), we discuss in section 2) the theoretical advantages of performing WE in neutral to high pH environments rather than in low pH environments. In section 3) the configurations of AEM-WE and its operation with pure water are described. The quality parameters of water and the cost of its purification are considered. In section 4) the fundamental aspects related to the thermodynamics and the kinetics of water electrolysis occurring in the presence of pure water with a circumneutral pH are highlighted. In section 5) the materials are described considering the main components of the AEM-WE, e.g., ECs, AEM and anion exchange ionomer (AEI). Their integration into MEAs and the effects of pure-water feed are discussed. Key properties and functions of the porous transport layer (PTL) and bipolar plate (BP) are presented. In section 6) the advantages and disadvantages of using pure water feed in AEM-WE are discussed in terms of performance. Comparisons with AEM-WE systems operating with supporting electrolytes are drawn and the intrinsic challenges to the use of pure water are highlighted. Last, in section 7) the durability and degradation phenomena of AEM-WEs operating with pure water feed are discussed as well as mitigation strategies.

## 2 Advantages of Anion-Exchange-Membrane Water Electrolyzers

To define the roadmap for the large-scale deployment of WEs and their diffusion and integration in the various sectors, a fair and critical comparison is crucial, considering their maturity stages, or in other words, the TRL. For practical application, the performance, cost, durability and sustainability must all be considered. For cost reasons, but also environmental impacts and supply chain risk, the utilization of CRMs in electrolyzer devices and other energy-related technologies is receiving increased attention. The list of CRMs is regularly updated in the EU by the EU Commission.^29^ While the criticality of a material can depend on a nation’s perspective, one can observe that the USGS 2022 CRM list and the EU 2023 CRM list have a strong overlap.

The chemical compositions of the various components differ for different types of low-temperature devices, in particular due to their exposure to liquids of vastly different pH values. The main components of low-temperature WEs are: 1) polymeric membrane and/or separator, 2) electrode/EC (anode and cathode), 3) porous transport layer (anodic and cathodic), 4) bipolar plate (anodic and cathodic), 5) frames and sealings. The simplified chemical composition and/or architecture of each component for A-WE, PEM-WE and AEM-WE is summarized in Table 1. Many materials utilized are not uniformly adopted by companies but can differ significantly, as highlighted by the colored cells of Table 1. Generally, it can be seen that for PEM-WE some platinum group metals (PGMs) such as Pt and Ir and some other CRMs such as Ti, and precious metals (e.g., Au) are systematically used (Table 1).^16,30^ In contrast, fewer (or no) precious metals are used in A-WE and this situation is also envisioned to apply to optimized AEM-WEs, without compromising the performance. However, especially in AEM-WE, Pt/C is still often used to catalyze the hydrogen evolution reaction (HER), due to the lower overpotentials achieved with Pt/C as compared to PGM-free electrocatalysts (ECs). In general, more expensive materials are used for BP, PTL and ECs on the anode side, which presents a harsh oxidative environment.

**Table 1:** Material Compositions of the Main Components of WEs^a^

Component	A-WE	PEM-WE	AEM-WE
Electrode/EC (oxygen side)	Nickel-coated perforated stainless steel	Iridium Oxide	High surface area Nickel or NiFeCo alloy
Electrode/EC (hydrogen side)	Nickel-coated perforated stainless steel	Platinum nanoparticles on carbon black	High surface area nickel
Anode side PTL	Nickel mesh (not always Present)	Platinum-coated sintered porous titanium	Nickel foam
Cathode side PTL	Nickel mesh	Sintered porous titanium or carbon cloth	Nickel foam or carbon cloth
Anode-side bipolar plate	Nickel-coated stainless steel	Platinum-coated titanium	Nickel-coated stainless steel
Cathode-side bipolar plate	Nickel-coated stainless steel	Gold-coated titanium	Nickel-coated stainless steel
Frames and sealing	PSU, PTFE, EPDM	PTFE, PSU, ETFE	PTFE, Silicon

a Components can vary among different companies. Data is reproduced from ref 16. Copyright IRENA (2020), Green Hydrogen Cost Reduction: Scaling up Electrolysers to Meet the 1.5 °C Climate Goal, International Renewable Energy Agency, Abu Dhabi.

### 2.1 Performance

The significant figures of merit related to the performance of WEs are taken from the Clean Hydrogen Partnership (CHP, former Fuel Cell and Hydrogen Joint Undertaking, FCH JU, initiative) targets.^31^ These figures are related to the stage of TRL where the low temperature WE stands. In other words, a comparison in performance between A-WE and PEM-WE can be considered fair, as the figures of merit are related to the system at the MW scale. Instead, direct comparison between A-WE and PEM-WE vs AEM-WE seems to be harsh to envision and be completely fair, as AEM-WE technology has not yet reached the readiness to be scaled up at the MW scale. However, those figures are important to take into consideration. In general, in the CHP targets, an increase in current density is envisioned concurrently with an improvement in stability and durability, a decrease in electricity consumption and a decrease in CRM used and consequently a reduction in costs.

State-of-the-art A-WEs performance metrics in 2020 are reported to operate at an imposed current density of 0.6 A cm^–2^ (1.9 V). Similar current density is envisioned for 2024 despite a decrease in: (i) CRM from 3.4 to 2.1 mg cm^–2^; (ii) degradation rate from 0.12 to 0.11% per 1000 h; (iii) electricity consumption at the nominal capacity from 50 to 49 kWh kg^–1^.^31^ State-of-the-art PEM-WEs (in 2020) should operate at an imposed current density of 2.2 A cm^–2^ (2.1 V).^31^ Whereas the target for 2024 is set for 2.4 A cm^–2^ (@1.95 V) and the target for 2030 is 3 A cm^–2^ @1.9 V. Moreover, other parameters are also proposed to decrease by 2024. Particularly: (i) PGM loadings in electrodes should decrease from 2.7 to 1.25 mg cm^–2^; (ii) degradation rate from 0.19% to 0.125% per 1000 h; (iii) electricity consumption at the nominal capacity from 55 to 52 kWh kg^–1^.^31^ On the other hand, a rapid performance improvement is expected as important efforts and resources have been recently devoted to AEM-WEs technology. The operating current density of AEM-WE is expected to increase from 0.5 A cm^–2^ (2.07 V) in 2020 to 0.6 A cm^–2^ (2.0 V) in 2024 and the final goal is 1.5 A cm^–2^ (1.8 V) in 2030.^32^ An important decrease in electricity consumption is expected over time from 55 kWh kg^–1^ (2020) to 53 kWh kg^–1^ in 2024 and the final goal is 48 kWh kg^–1^ in 2030.^32^ Importantly, the utilization of CRMs is expected to decrease from 1.7 mg W^–^^1^ in 2020 to 0.4 mg W^–1^ in 2024 and is envisioned to eventually disappear in 2030.^32^ However, following the IRENA analysis that makes a screenshot of the current situation, it can be seen that today the operating current density of AEM-WE is variable (0.2–2 A cm^–2^) with a recorded voltage of 1.4–2.0 V.^16^ IRENA analysis envisioned a long-term target of >2 A cm^–2^ for 2050 (at a cell voltage <2 V).^16^ The present performance attributes and the expected trends of PEM-WE at acceptable efficiency (cell voltage) are much higher compared to A-WE, although this is offset by the higher capital cost of PEM-WE, which has to be mitigated seriously.^20^

### 2.2 Hydrogen Pressure and Safety

Direct production of pressurized hydrogen (30–70 bar) within WEs is an important goal because it strongly reduces the additional energy needed for the final compression to 350–700 bar, needed for transportation. In WEs, the hydrogen can be pressurized internally with only a small penalty (increase) in thermodynamic cell voltage. The latter is calculated to be 34 mV at 25 °C and 48 mV at 150 °C when varying the gas pressure from 1 to 200 atm and considering that the gas pressure is the same at both electrodes. Recently, a voltage drop of 55 mV due to an increase in the differential pressure was calculated at 60 °C.^33^

While thermodynamics does not predict a strong performance penalty upon high internal pressurization of H_2_ (and O_2_), the practical WEs might undergo mechanical stress, component failure, or safety issues. A-WE does not historically possess a physically continuous separator, as the diaphragm is porous. Therefore, high differential pressure between the anode and cathode compartments leads to a high crossover of the gaseous products, with relevant safety issues and potential explosions. Moreover, as the separator is porous, the produced hydrogen in A-WE might have lower purity (99.0 to 99.5 vol.%) compared to PEM-WE, especially when operating intermittently, and it needs to go through drying and additional oxygen removal before reaching the required purity.^14^ In practice, A-WE operates at low internal pressure (up to ∼ 20 bar, balanced with O_2_ and H_2_) and therefore the hydrogen produced needs to be pressurized externally with additional capital (CAPEX) and operational (OPEX) costs of mechanical compressors. The use of nonporous polymeric ionomer membrane, such as in the case of PEM-WE and AEM-WE, allows the direct production of pressurized hydrogen with a relevant reduction of operating costs, without significantly pressurizing the produced oxygen that is typically vented to the atmosphere. The polymeric membrane separator also allows the production of hydrogen with a high level of purity (typically up to 5.0 grade or 99.99999 vol.%).^32^ PEM-WE and AEM-WE also display higher performance than A-WEs due to relatively thin membranes, leading to lower cell ohmic resistance than A-WEs. However, thin membranes can face mechanical failure at lower operating pressure, and overly thin membranes can lead to significant H_2_ or O_2_ crossover. Oxygen crossover through PEM was evaluated in studies related to PEM fuel cells, where hydrocarbon membranes showed 1 order of magnitude less oxygen permeability compared to fluorinated polymers.^34,35^ Hydrogen and oxygen crossover through AEM is less studied. In PEM-WE, recombination layers are used on the anode side to enable the reaction between the hydrogen being permeated through the membrane and the generated oxygen, forming water. In this way, the concentration of hydrogen in the anodic oxygen flow remains below 4 vol.%, avoiding eventual safety issues.^36^

A compromise between membrane thickness and operating pressure must be found to ensure optimal performance, without jeopardizing the stability and durability of the system.^37^ Due to the maturity of PEM, know-how and greater investment in this technology, at the moment, PEM-WEs can operate at higher pressure compared to AEM-WE. According to the IRENA analysis, the general goal is to increase the current system pressure.^16^ The envisioned Key Performance Indicators (KPIs) by IRENA for 2050 are >70 bar (A-WE), > 70 bar (PEM-WE) and >70 bar (AEM-WE).^16^

### 2.3 System Cost and Critical Raw Materials’ Utilization

While briefly analyzing the materials used for low-temperature WEs, as mentioned above, PEM-WE relies strongly on PGMs.^30^ For instance, Pt is not only used in the PEM-WE cathode EC, but it is also a crucial component in the anode PTL and BP for corrosion protection in harsh oxidative environments. The loading of Pt is quite high and it heavily affects the overall cost of the device.^16^ Moreover, the use of CRMs is economically nonviable due to uncertain supply and skyrocketing costs since their mining is localized in a few socio-politically unstable regions. A-WEs involve CRM-free components that are stable in the alkaline operating environment; hence, cost-effectiveness and scalability can be ensured. On the other hand, although the performance attributes of AEM-WE are encouraging, it is still in the development phase and significant scientific efforts are needed to become commercially rationalized. AEM-WEs are currently relying on PGMs, especially on the cathode where Pt/C is used; however, in the medium or long-term perspective, CRM-free components are expected to be widely used in AEM-WEs as highlighted by the roadmap identified by the Clean Hydrogen Partnership (CHP) for 2030.^32^ Another factor is the operational durability, PGM nanoparticles supported over the carbon, typically employed at the AEM-WE’s cathode, are highly unstable in the KOH electrolyte.^38–42^ and may also undergo degradation at the AEM interface.^43^ when subjected to potential cycling-a situation that is unpreventable during the startup and shutdown operations. Therefore, similar to A-WE, the use of CRM/PGM-free materials will not only lower the CAPEX of the AEM-WE but also help in enhancing the performance durability.

### 2.4 Life Cycle Considerations

In scaling technology, environmental impacts and sustainability are of utmost importance. CRMs imply problems related to the high cost and shortage in the supply chain in the case of large utilization, but they also mean difficulties in mining and material purification. Despite these environmental issues and recent interests, to date, there is no valid procedure or internationally shared protocols to recycle PGMs derived from WEs and fuel cells.^44–46^ Anyways, A-WEs and AEM-WEs can rely on CRM-free components, which should also translate to a lower carbon footprint during manufacturing and environmental safety.

Another important aspect is the use of fluorinated polymers in PEM-WEs. These polymers are based on per- and polyfluoroalkyl substances (PFAS) that are resilient to degradation, accumulate in the environment (mainly in water and soil) and can enter and accumulate in the food chain, negatively affecting the whole ecosystem.^47^ PFAS can also accumulate in human bodies and cause a series of negative and deadly diseases.^48,49^ The European Union has issued a ban on PFAS for 2035,^50^ and this could affect the manufacturing and sale of PEM-WE, despite the WE is a closed system where PFAS can be prevented by proper handling at the end of life and during water changes. Despite alternative hydrocarbon-based ionomers having been investigated for more than a decade,^51^ a robust and resilient substitution has not yet been found. The membrane chemistries in A-WE and AEM-WE typically do not use PFAS. Teflon can be used for sealing, but alternatives can be found.

A recent study on life cycle assessment (LCA) on water electrolyzers highlighted that AEM-WE technology has a lower environmental impact compared to PEM-WE in 24 impact categories (out of 27), including climate change.^52^ Naturally, this comparison is not exactly fair since PEM-WE is commercially available while AEM-WE is still not fully developed. On a positive note, a large room for improvement is foreseen and further eventual decrease in the environmental impact can be expected.

## 3 Configurations and Operating Modes AEM-WEs

Given the considerations discussed above, AEM-WE will be suited for hydrogen production at a massive scale if the envisioned performance and durability levels can be reached. Given the low current TRL, tremendous R&D efforts are needed to bring deployable AEM-WE, particularly for systems with pure water feed that truly combine the advantages of both PEM-WE and A-WE. Operating with a water feed and locally alkaline environment, PGMs could be potentially substituted with PGM-free and CRM-free materials. This aspect is crucial because, to date, Pt/C is still the best EC for HER in alkaline media and important work is still ongoing and needed to fully replace PGMs at the cathode.^53,54^ The substitution of PGM with PGM-free materials is beneficial in terms of cost, usage, or rare and critical materials and so it contributes to the wide commercialization of water electrolyzers.

Compared to A-WE, the use of a polymer membrane in AEM-WE is important for driving performance due to the vicinity of the electrodes assembled on the membranes lowering the ohmic resistance of the MEA. The presence of a dense and nonporous polymeric membrane allows the pressurization of hydrogen without compromising the safety of the device. Continued engineering and thinning of the membrane may lead to dramatically higher currents in AEM-WE. The lack of liquid electrolytes and the presence of flow fields in the BPs may further facilitate gas collection compared to A-WE.

The main configurations for feeding the water/electrolyte in AEM-WEs are reported in Figure 3. The first one involves feeding them with supporting electrolyte (e.g., KOH) in both the anode and cathode compartments (Figure 3.A) or simply to the anode side (Figure 3.B) or the cathode side (Figure 3.C). The second configuration involves the feeding of pure water that can be fed to both compartments (Figure 3.D) or is fed uniquely at the anode (Figure 3.E) or the cathode compartment (Figure 3.F). The configuration in which pure water is fed uniquely to the anode side is the one used commonly in PEM-WE, in agreement with the fact that water is the sole anode reagent in acidic OER.^30,55^ All the configurations shown above are used and up to now, there is no clear and conclusive operational configuration that outperforms the others. Interestingly, the configuration with only anode feeding (Figure 3.B and 3.E) is counterintuitive in alkaline conditions. This is because water is a reagent for the hydrogen evolution and therefore it would be required at the cathode compartment for evolving the gas of interest. In practice, water is provided for HER by osmosis, diffusion, or drag from the anode compartment. Interestingly, this configuration is often more efficient because hydrogen is produced with the cathode electrode not submerged in a liquid and, therefore, the release of gaseous hydrogen encounters lower resistance than if water (or KOH) were fed to the cathode. The produced hydrogen also includes a lower quantity of water in such a configuration, which is beneficial because the hydrogen produced needs to undergo dewatering before being further pressurized to be stored or used. However, in some cases, other water feed modes may be advantageous.

**Figure 3:**
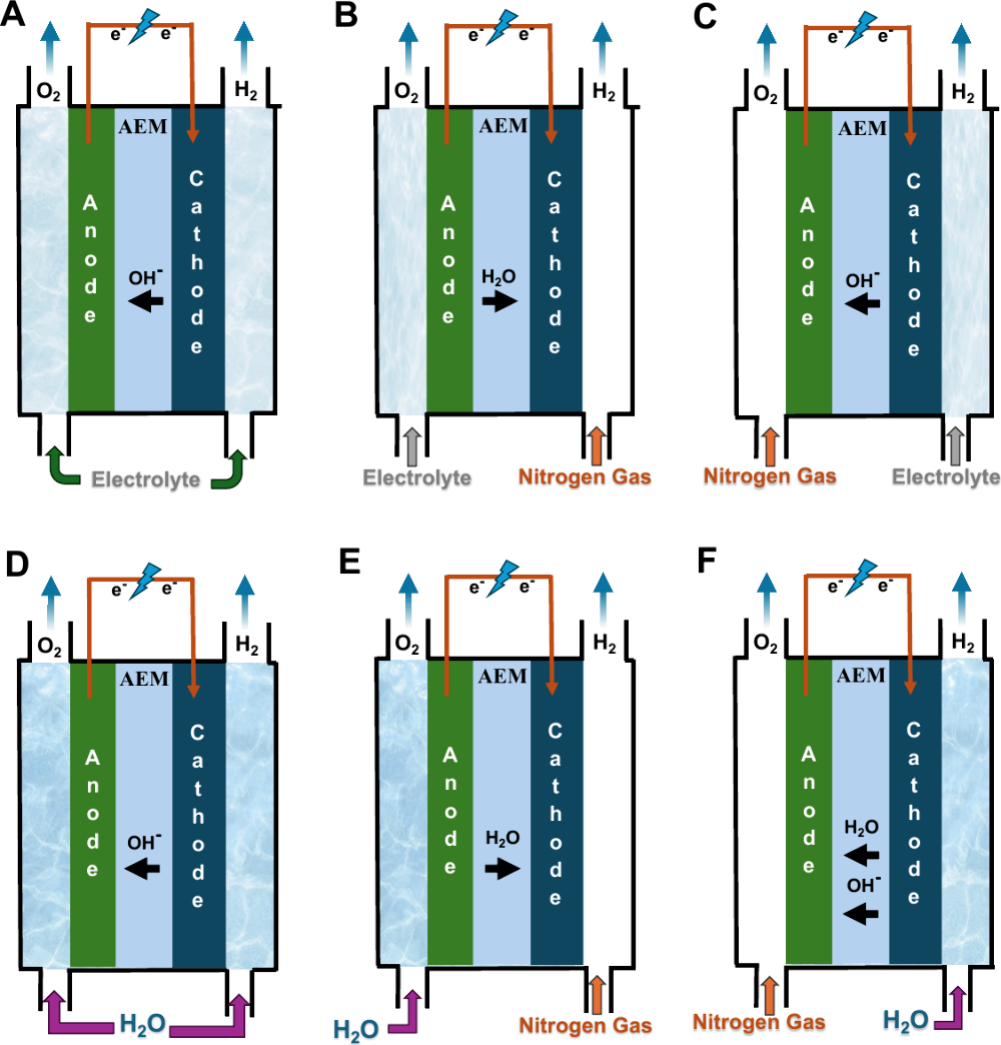
Schematic of the operating configurations for AEM-WE with supporting electrolyte fed on both anode and cathode (A), or only fed to the anode side (B) or the cathode side (C). Schematic of the operating configurations for AEM-WE with pure water fed on both the anode and cathode (D), or only fed to the anode side (E) or the cathode side (F).

Another important aspect is to evaluate what is actually occurring within the core of the single-cell AEM-WE, named the MEA. A cross-section of a single cell AEM-WE is schematized in Figure 4. Key interfaces for both electrical and ionic interconnections among the different layers are identified and highlighted. Transport phenomena occurring at different scales and involving different layers and different electrically and ionically conductive components are also labeled in Figure 4. Importantly, the spatial scale of the transport phenomena taking place in the various components is reported.

**Figure 4:**
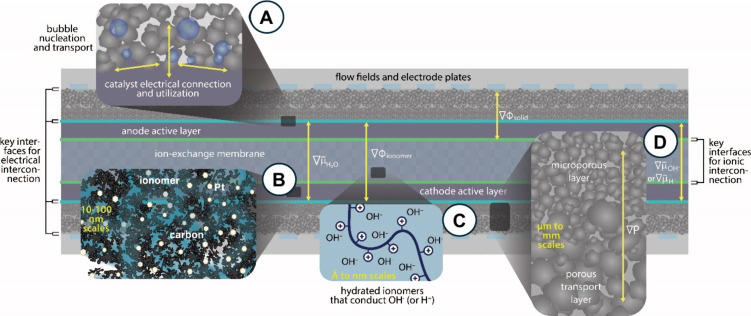
Cross section of a single cell AEM-WE identifying the key interfaces for electrical and ionic interconnection and particularly highlighting (A) catalyst electrical connection and utilization; (B) carbon/platinum interaction with the ionomer; (C) Hydroxide transport within the AEM; (D) pressure gradient within the catalytic layer.

### 3.1 AEM-WE Operating with Different Electrolytes

AEM-WE operates with a wide array of feeding solutions, including deionized water (DI),^56–60^ seawater and brackish water,^61–66^ impure tap water,^67^ or water with supporting electrolytes such as potassium hydroxide (KOH),^68–71^ sodium or potassium carbonate (Na_2_CO_3_ or K_2_CO_3_)^19,72^ and others. To date, however, none of these feed solutions, except for KOH, provide expected performance and durability metrics for commercialization. There are many possible issues with fouling of the electrodes with impure feeds. Further, any salt present will lead to concentration polarizations that reduce efficiency through a concentration overpotential. A simple example is the use of a carbonate feed. Carbonate concentration polarization leads to a pH gradient across the device and an increase in the Nernstian voltage for electrolysis.^73^

### 3.2 Justification of Operating AEM-WE with Pure Water

Scientists and industries are increasingly interested in utilizing pure or distilled water for the operation of AEM-WEs. In parallel with the PEM-WE, the AEM-WE operating with purified water brings a complication since purified water needs to be produced. The water available on the planet is 97% seawater and 3% freshwater.^74^ Seawater is too salty to be consumed directly (“as it is”) by humans or animals, to be used to grow crops and it is quite complicated (if not impossible) to be used for industrial purposes, except for very specific purposes (e.g., cooling). Fresh water supply is under stress due to the anthropogenic impact on the environment and this stress is increasing significantly with time. Each year, humanity consumes 4600 billion m^3^ of water for its activity, 70% of which is spent in agriculture, 20% in industry, and 10% in domestic usage.^75^ These numbers are the result of a steady increase in fresh water consumption of roughly 1% per year since 1980,^76^ every single drop of which comes from the freshwater reservoirs of Earth, accounting for 3% of the global water.^77,78^ The concern about water affordability for the future is thus real and mainly related to the increase in world population, directly translating into increased food and drinking water requests along with energy demand. Actually, within the water consumption of the industry sector, 75% of that is related to the conversion of energy, with the most demanding energy sources being biofuel, fossil fuel, and nuclear energy.^79^ The production of renewable electricity directly from wind and solar does not need water for cooling or operating and the impact on water consumption (volume of water per MWh electricity) is *ca* 100 times lower than that of fossil fuels and nuclear power plants.

In the first decade of this century, some researchers expressed their skepticism about the hydrogen economy,^79–81^ especially in terms of water consumption. However, in the past decade, a vast research effort improved the efficiencies of the WE, paving the way to the mass production of green hydrogen with limited impact on planet resources. Regarding the impact on Earth’s water reservoirs, a distinction must be made between water withdrawn and consumed: the former is intended as the quantity of water that is extracted and then returned to the whole body of water, while the latter refers to the amount that is lost. A recent study by Oliveira et al.^82^ estimated the future total demand for green hydrogen at 2.3 Gt per year, corresponding to 20.5 billion m^3^ of withdrawn freshwater.^83^ These numbers were calculated considering the stoichiometric ratio of the water splitting, which requires 9 kg of water to produce 1 kg of hydrogen. However, considering the requirement of distilled water for the WE operation, the multiplication coefficient could be higher. Other authors have calculated a global water demand of 34.5 billion m^3^.^84^ Nevertheless, in comparison, the power generation and energy production from fossil fuels withdrew 251 billion m^3^ of freshwater and consumed 31 billion m^3^ in annexed operations like mining, cooling, refining, etc.^85^ At the same time, agriculture impacts on freshwater account for 2700 billion m^3^, more than 40% of which is consumed.^85^ It is thus straightforward that future water utilization for hydrogen production will have a modest impact on the environment, estimated at 1.5 ppm of Earth’s total freshwater.^83^ Moreover, the circularity of the economy already sets alternatives to water withdrawal, exploiting waste products that must be processed before discarding, e.g., wastewater produced in large amounts each day. This could help to boost the spread of the technology in developing countries and especially in dry/desertic regions of the planet where access to fresh water is limited,^84^ leading to a real zero impact on freshwater reservoirs along with reduced effects on the environment. In addition, the volume fraction of water for the projected demand of H_2_ can be further reduced if seawater is considered instead of freshwater. In this case, desalination operations and their water consumption must be considered in the life cycle assessment of WE, leading to an amount of water required for green hydrogen that was estimated at 30 ppb of seawater per year.^83^ The use of seawater to produce green hydrogen can thus be considered negligible.

The possibility of operating AEM-WE with impure, or at least less purified, water was recently shown^86^ and compared to the distilled water used in PEM-based technology. Therefore, it would seem logical to operate the AEM-WE with readily available seawater due to its abundance and lower effect on resource availability. However, the presence of anions and cations, solids and microorganisms^67^ might lead to a faster deterioration of materials, leading to a shortening of the useful lifetime of the AEM-WE^86^ challenging the targeted durability in the order of thousands of hours.

Therefore, irrespective of the primary source, water fed to the AEM-WE must be purified. The three main methodologies for such a purpose are (i) thermal treatments, usually based on solar or geothermal heat like multiple effect distillation (MED) where seawater is evaporated, cooled, and condensed, (ii) membrane distillation, based on thermally driven physical filtration through semipermeable membranes, and (iii) reverse osmosis (RO) based on pressure-driven membrane filtration.^87,88^ Actually, the trend of this field of research is to develop hybrid or cogenerated systems to reduce costs and increase the efficiency of energy recovery.^89–92^ Different purification processes, along with a comparison of cost per m^3^ and per kWh, were extensively revised by Yadav et al.^87^ Indeed, the MED system is considered more expensive, with 80.6 kWh of heat per m^3^ of freshwater produced, while RO is less energy demanding with just 3–5 kWh of power per m^3^ of purified water.^93^ It is thus straightforward that RO will be the leading technology to be coupled with AEM-WE, with estimated costs in 2009 of 0.5 – 1.5 USD per m^3^ of purified water,^77^ which decreased to 0.33 USD per m^3^ in 2018.^89^ These numbers are not negligible but strengthen the direction of pursuing the utilization of purified water as a feeding electrolyte for AEM-WE since the cost and energy used to purify the water impact the total cost of 0.01 USD per kg of H_2_ produced.^83,94^ Naturally, these considerations are done considering only the operational costs, without considering the CAPEX.

## 4 Thermodynamics and Kinetics of Water Electrolysis

### 4.1 Bird’s Eye View of the Energetics of Water Splitting in Alkaline Media

This section focuses on defining the most relevant aspects of water electrolysis that are influenced by pH variations, which can occur locally in pure water electrolysis systems. It is well-known that pH, along with other factors like impurities in the water and the nature of supporting electrolytes, significantly impacts the energetics of electrolysis. Both the HER and oxygen evolution reaction (OER) are pH-dependent, with the most notable effect observed on HER, which is much slower in alkaline and neutral electrolytes compared to acidic systems.^95^ To understand and rationalize these effects, as well as to explore strategies for improving performance, this section consists of an overview of the chemical thermodynamics of water splitting and the kinetics of both OER and HER for alkaline systems.

Water is a notoriously stable molecule and the water-splitting reaction (eq 1) has a Gibbs free energy which defines a theoretical cell voltage of 1.23 V between the anode and the cathode of a WE, at standard conditions.

$$H_{2}O\to H_{2}+\frac{1}{2}O_{2}$$ 1

Electrolyzers usually operate at a larger cell voltage than the theory predicts, as many other factors significantly contribute to the overall voltage when the cell delivers a current. The different contributions to the total voltage of an operating WE can be subdivided according to eq 2

2 $$E_{ap}=1.23V+\eta_{HER}+\eta_{OER}+\Delta V_{other}$$

where *η*_*HER*_ and *η*_*OER*_ are the overvoltages for hydrogen and oxygen evolution, respectively. From eq 2, it can be seen how thermodynamics and kinetics both affect the operating potential quantitatively. These values depend strongly on the current density and the type of ECs, as the activation energy of both reactions is strongly affected by the nature of the catalytic surface. In addition, some potential (ΔV_other_) is needed to overcome the resistances of electrolytes, contacts, and membranes, as well as the parasitic resistance of side reactions such as electrode corrosion and oxygen reduction.^96^

The dependence of the thermodynamic potential of water splitting on pressure and concentration is described by the Nernst equation (eq 3).

$$E=E_{o}\left(T\right)+\frac{RT}{2F}\ln(\frac{a_{H_{2}}\Delta a_{O_{2}}^{1/2}}{a_{H_{2}O}})$$ 3

Remarkably, eq 3 shows that the thermodynamics of water splitting does not depend on pH. Current AEM-WE technologies can reach 1 A cm^–2^ even at less than 2 V using KOH electrolytes, depending strongly on whether PGM or PGM-free ECs are used. These values cannot be achieved with conventional alkaline electrolysis (A-WE), where the maximum operating currents are generally around 0.7 A cm^–2^.^31^ Depending on the applied current density, overpotentials of the half-reactions (HER and OER) contribute to the overall water splitting overpotential to the extent of 30–40%. To understand the chemical basis of this, it must be considered the cathode and anode half reactions of water occurring in alkaline systems that are described by eq 4 and eq 5:

$$\mathrm{CATHODE HALF REACTION}:2H_{2}O+2e^{-}\to H_{2}+2OH^{-}$$ 4

5 $$ANODEHALFREACTION:4OH^{-}\to O_{2}+2H_{2}O+4e^{-}$$

It is worth mentioning that eq 4 and eq 5 are only the total electrochemical reactions taking place at the cathode and anode, respectively. To fully understand the relevance of the pH in the kinetics, and its implication in high or mild alkaline environments or with pure water (circumneutral pHs), the elementary steps occurring in HER and OER need to be broken down and the rate-determining steps (RDS) must be identified. A view on the current knowledge in HER and OER kinetics is the focus of the next two subsections, where, despite the extensive research efforts, many aspects remain unsolved. In fact, a universal descriptor for alkaline HER and OER able to predict the behavior of all the different EC families is still a “Chimaera”.

### 4.2 Electrochemical Kinetics of Alkaline Electrolysis

#### 4.2.1 Hydrogen Evolution Reaction

In alkaline systems, the kinetics of the HER is 2–3 orders of magnitude slower than in acids and becomes comparably limiting to the OER process at the anode, even with PGM-based ECs. A strong research focus on HER in alkaline and on PGM-free (CRM-free) ECs is hence justified, with a huge potential to deliver solutions to increase the energy efficiency of electrolysis.^97^

The widely accepted breakdown of the alkaline HER into its elementary steps is described by the set of eqs 6 and 8 or eqs 7 and 8.^98^

6 $$H_{2}O+e^{-}+M↔M-H_{ad}+OH^{-}$$

7 $$H_{2}O+M-H_{ad}+e^{-}↔M+H_{2}+OH^{-}$$

8 $$2M-2H_{ad}↔2M+H_{2}$$

Equation 6 is known as the Volmer step, which consists of the adsorption of hydrogen onto the metal. Remarkably, this step differs for alkalis from that for acids because the proton source is water, not hydronium ions.^99^

The adsorbed hydrogen can then be converted into molecular hydrogen either by the Heyrovsky step (eq 7), or the chemical Tafel recombination (eq 8). The use of the Tafel slope can help in identifying the rate-determining step (RDS). Typically, it is ca. 120 mV dec^–1^ when the Volmer step is rate-determining, while it is ca. 40 and 30 mV dec^–1^ when the Heyrovski or Tafel step is rate-determining, respectively.^100^ Thus, the identification of the RDS due solely to the value of the Tafel slope might be considered. However, the surface coverage of the adsorbed hydrogen may also play a crucial role in the reaction kinetics. Indeed, Tafel slopes of 120 mV dec^–1^ have been observed for the HER when the coverage of adsorbed hydrogen exceeds 0.6, even when the RDS is the Heyrovsky step. The Tafel slope is also potential dependent and, according to microkinetic models, this may result in turn from the potential dependence of the adsorbed hydrogen coverage. A comprehensive picture of the HER can be obtained from microkinetic models based on accurate atomistic modeling.^101^

In an alkaline system, as in acids, the HER activity follows a typical volcano plot. The Volcano diagram is obtained by plotting a parameter related to the reaction rate (e.g., current density normalized by the electrochemical surface area, or active site turnover frequency, or exchange current density) against the adsorption binding energy. This can be done by plotting the exchange current densities as a function of the hydrogen binding energies across a range of materials.^102,103^ Figure 5.A and 5.B show that a volcano plot for the HER activity is obtained across different metallic surfaces in both acid and alkaline electrolytes.

**Figure 5:**
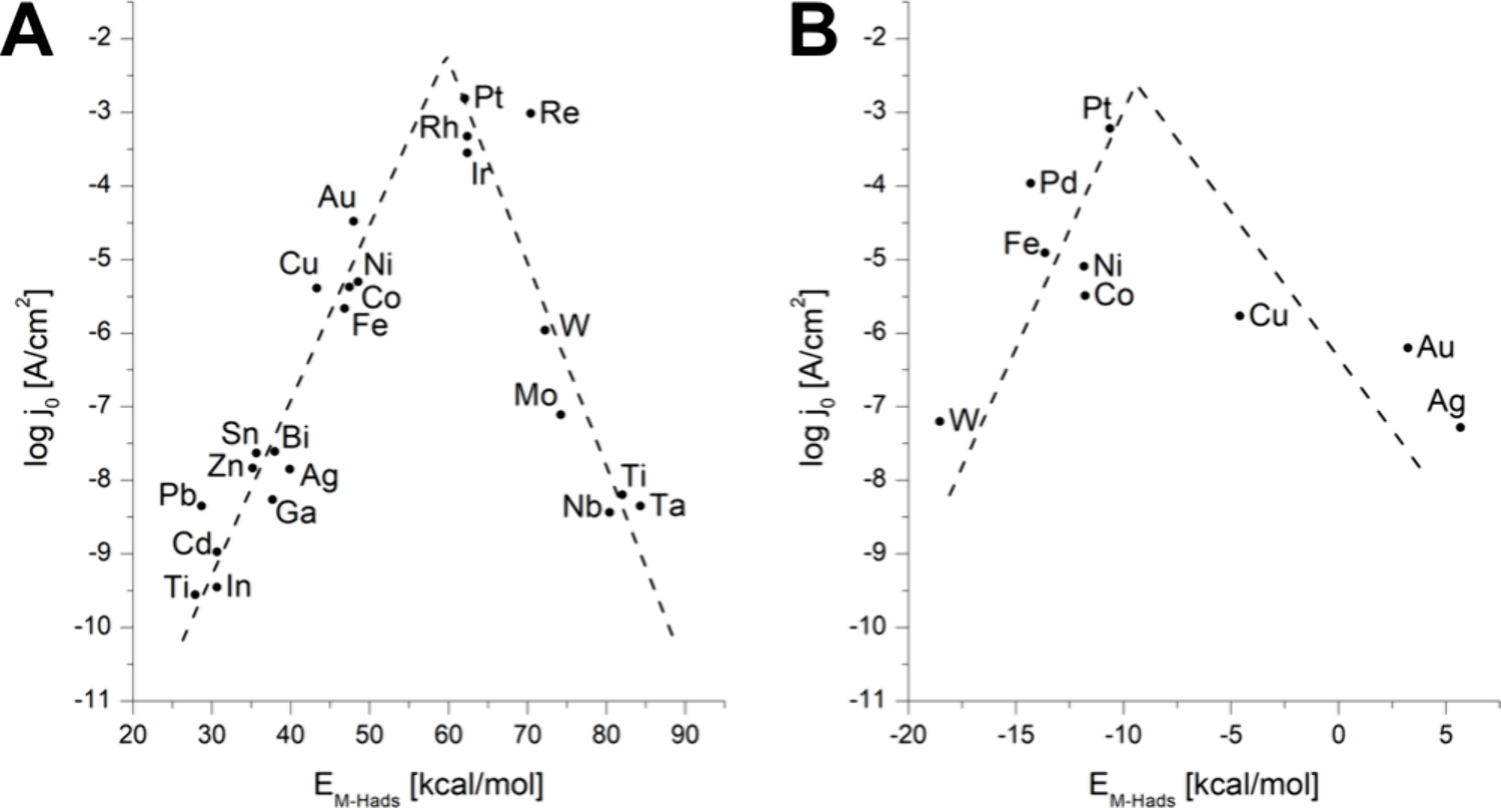
Volcano plots of several transition metals for HER in (A) acidic and (B) alkaline conditions. In (A), E_M‑Hads_ is an experimental value, an operative electrochemical adsorption heat used by Trasatti for its plot, and reprinted with permission from the work of Krishtalik, ref 104. Copyright 2010, American Chemical Society (ACS). In (B), E_M‑Hads_ was calculated using DFT, reprinted with permission from ref 105. Copyright 2013, The Royal Society of Chemistry.

The volcano diagram results from the free energy of hydrogen adsorption.^106,107^ In the case of a weak binding, adsorption is the rate-determining step. This is the so-called Volmer step (eq 6). In the case of a very strong binding, desorption dominates the rate and the desorption steps, i.e., Heyrovsky/Tafel (eqs 7 and 8), control the reaction. To achieve the highest activity, the hydrogen bonding at the EC surface must be neither too strong nor too weak. Accordingly, the necessary condition for an active HER EC is that the free energy of adsorption is close to zero.^106,107^ The Sabatier principle is the result of such just-right adsorption energy and does not hold only for the HER but also for many others. The Sabatier principle and the volcano plot give indications on how to design ECs that have high activities. While the use of volcano plots can certainly deliver an excellent indication, it is not the only element to consider when designing ECs. More accurate and efficient techniques of computing electrochemical barriers for proton transfer processes involving both hydroxide and hydronium ions^103,108–111^ are needed for a complete quantitative understanding of these effects.^112^

Figure 6 shows the dependence of the cyclic voltammetry of Pt (111) on the pH. No significant changes in hydrogen underpotential deposition are observed in the alkaline environment (0.10–0.35 V vs RHE). However, HER shows a significant shift of the overpotential to a more negative value with the increase in pH. Remarkably, the dependence of the rate of HER on pH is much more pronounced than the dependence on hydrogen underpotential deposition. It can be noticed that the hydrogen underpotential deposition is related to the hydrogen binding energy and that accordingly, the latter does not depend on the pH. This is a clear indication that the significant variation of the HER rate at a given overpotential on Pt (111) is not related to the negligible shift in hydrogen underpotential deposition, a fact that raises concerns about the use of the hydrogen binding energy as a single descriptor for the HER.^113^

**Figure 6:**
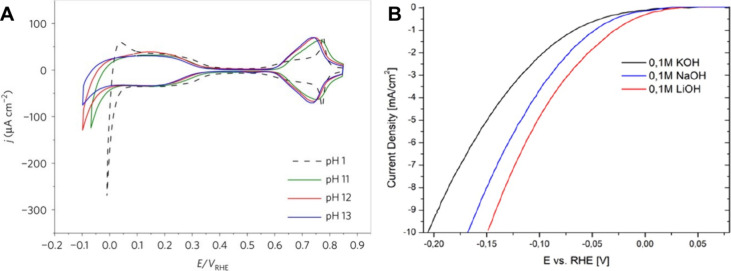
A) The dependence of the HER rate on the pH. Comparison of cyclic voltammetry scans on Pt (111) at various pH. Adapted with permission from ref 113. Copyright 2017, Springer Nature. B) Linear sweep voltammetry in the HER region of polycrystalline platinum in 0.1 M alkali with different cations. Modified with permission from ref 114. Copyright 2022 Springer Nature Limited.

The poor activity in alkali is the result of the low proton concentration and the slow water dissociation that produces a poor supply of dissociated hydrogen to the surface of platinum.^115^ To overcome this limitation, the synergistic effects of two or more elements can be considered. It has been demonstrated that the addition of TMs, e.g., Ni, Co, Fe, to Pt, even in the form of oxides, skyrockets the Volmer step reaction rate and also increases the adsorbed hydrogen supply for the Heyrovsky and Tafel steps.^116,117^ The role of the interface in this EC seems to be extremely relevant. Indeed, it was first speculated that the water dissociation happens mainly at the interface between platinum and the transition metals (TMs), where the adsorbed OH and H bind respectively with the transition metal and Pt. This highlights the importance of increasing the ratio between the interfacial Pt and bulk Pt atoms, which is generally very small. However, a study by Markovic et al. demonstrated that water dissociation happens mainly inside the TM regions. Accordingly, the formed adsorbed hydrogen should travel to the platinum areas where it might undergo recombination and desorb to release gaseous hydrogen.^118–120^ This is the hydrogen spillover that might provide clear indications on how to boost the activity of HER ECs. The hydrogen spillover consists of the migration of adsorbed hydrogen from the regions where it is formed to regions where it is weakly adsorbed.^121–124^ This is the case of platinum, which shows moderate values of H adsorption energies.

The spillover effect might be used to make ECs that are relatively insensitive to pH conditions. Indeed, in ref 125 it was argued that spillover can provide significant hydrogen evolution activities also in a neutral or basic environment. For example, oxygen-deficient tungsten oxide promotes the HER activity of ruthenium.^126^ Also, Ni­(OH)_2_ has been shown to promote HER activity on Pt.^127^ The presence of oxygen vacancies in tungsten oxides boosts the proton-storage capacity and the charge-transfer rate by increasing the hydrogen supply to the ruthenium metal surface, where the hydrogen can recombine and desorb as H_2_. Water dissociation occurs on the tungsten-oxide surface in the presence of the electrochemical double layer^128–130^ generating protons that spill over to the ruthenium surface.

Based on changes in the Tafel slope, the spillover appears to change the RDS from the water dissociation reaction (Volmer step) to the hydrogen recombination (Tafel of Heyrovsky step). Multicomponent ECs thus offer the opportunity to tune the binding energies of the hydroxyl and hydrogen at different catalytic sites^131^ which is central to minimizing HER overpotential in AEM-WE, and has been hypothesized to increase HER activity by simultaneously reducing the H_2_O dissociation and H_2_ formation energies.^132–135^ Both the hydrogen and hydroxyl binding energies are important in determining HER activity,^136^ where binding hydroxyl is central to the water-dissociation step, releasing protons to form metal hydrides ultimately on the metallic catalyst surface.

Indeed, in these cases, the excessive coverage of the surface may result in EC poisoning that depresses the activity. Again, spillover may help to mitigate this effect by increasing the rate of H removal from the surface.

Related spillover effects are also central to achieving fast hydrogenation catalysis with thermal processes.^137,138^

Based on the above work, spillover effects are evidently an important tool in designing advanced catalysts for alkaline HER in AEM-WE. These advanced ECs might be designed to be resilient toward local variation in the pH that might occur for the combination of the low OH^–^ mobility and the use of pure water in ionomer/catalyst porous electrodes. However, developing a theoretical framework that supports the development of ECs is not trivial. The question of the factors that determine the HER rate is still debated and there is no single descriptor that can be used to explain the activity trends of ECs even in the simplest case of Pt in acidic electrolytes, let alone the complex situation of multicomponent surfaces and spillover in neutral to basic media.^115^ Koper and co-workers reported a 3D volcano plot that considers both hydrogen and hydroxyl binding energies.^139^ These relationships are available for a limited number of metals; however, they may represent a more comprehensive model for the HER activity.

Other authors suggest that the role of hydroxyl might be indirect, e.g., in stabilizing the transition state or modifying the interfacial water orientation. For example, some have argued that the operating potential for the reaction compared to the potential of zero free charge (PZFC) is critical. Near the PZFC, the interfacial water has high entropy, allowing facile water reorganization as ions transfer through the electrochemical double layer. In some cases, for example, the transfer of the adsorbed OH to form OH^–^ in the bulk electrolyte may be a descriptor.

All these hypotheses and underlying mechanisms have been supported by select experiments, but fail to be universal. Problems arise from the nature of many ECs, which often have complex surface structures, absorbed species (especially from impurities^140^), and are not easy to model. The presence of nanoparticles with surfaces rich in defects and a variety of terminations in a hybrid system of interfaces further complicates understanding. The contact between phases further introduces electronic effects due to interfacial charge transfer and Fermi energy equilibration.

The presence of metal cations in the supporting electrolyte can also significantly affect the reaction rate, and a variety of explanations have been proposed. Particularly, it has been argued that the role of the cation is linked to the role of adsorbed hydroxide in the HER. In ref 141 the authors propose that cations stabilize the OH at the interface and the transition state of the water dissociation, which is thought to be rate-limiting for the noble metals in alkali. It was shown that a Brönsted-Evans–Polanyi relationship exists between the energy of the transition state for the dissociation of water and the adsorption energy for the hydroxide.^139^ According to this model, a cation stabilizes the transition state for water, enhancing the HER rate, particularly for metals like Au, Cu and Ag. More oxophilic metals that absorb hydroxide strongly (e.g., Ir, Pt, Pd) have a smaller activation barrier for water dissociation and the removal of the hydroxide from the surface becomes relevant. It was shown that in alkaline media, the HER rate decreases with the increase of cation size on reactive metals (Ir, Pt, Pd),^141,142^ while an opposite trend is observed with noble metals. Even in the absence of a clear interpretation framework, it is likely that the mobility of the cation within the double layer is important. Large cations have weaker coordination with the water molecules, allowing larger mobility compared to smaller cations that bind water more rigidly.

A step forward in the understanding of the cation effect is reported by Shah et al.^114^ Smaller solvated cations at the Pt surface were found to increase the coverage of adsorbed OH in the HER potential window. The adsorbed OH was proposed to act as an electronically favored proton acceptor, explaining the trend of increasing HER activity with decreasing size of the cation (Figure 6.B).^114^

For Au at pH 11, the increase in the cation size is beneficial to the HER rate, and the reaction order for the cations is 0.5. The authors concluded that the cation is central in determining the activity and that this is related to the stabilization of the transition state of the Volmer step by interacting favorably with the dissociating water molecule. Moving from pH 10 to pH 13 was thought to affect the local field strength, enhancing the HER rate by affecting the concentration of the local cation. It was also argued that excess near-surface cation concentration negatively affects the HER rate.

The discussion so far has focused on explaining HER performance of different ECs as a function of the structure and composition of the catalytic surface and also on the composition of pure and well-defined alkaline electrolytes. Yet in pure-water-fed AEM, the only cations present are those associated with the anion-conducting ionomer phase, typically quaternary ammonium groups bound to the polymeric backbone. It remains an open question how these typically larger and less mobile cations affect the interfacial water, surface-absorbed hydroxide, and local transport. Depending on the precise nanoscale and local structure and interactions between the HER EC and the ionomer, these effects could vary substantially. For example, with pure-water feed, even the local pH at the active surface of the EC is uncertain and likely depends on the magnitude of the current and specific electrode processing and ionomer character. The situation is not different from PEM technology; however, OH^–^ transport in liquid water is three times slower than proton transport, leading to larger concentration polarizations (pH gradients), providing a significant concentration gradient and eventually pH differences between the anode (locally getting more acidic with current due to hydroxide consumption) and the cathode (locally getting more basic with current due to hydroxide generation). Understanding these effects thus remains an important area for future analytical work in device-relevant architectures to enable improved design, but it is apparent that electrocatalysts resilient toward local pH variation might help in promoting reaction kinetics and improving device performance and durability.

#### 4.2.2 Oxygen Evolution Reaction

The mechanism of the OER is more complex compared to HER, consisting of at least four elementary steps involving the transfer of electrons and protons (or, equivalently, hydroxide/water).^143^ Correspondingly, the OER is slower with kinetic overpotentials in the best cases of ∼ 300–400 mV at relevant current densities of ∼ 0.5–2 A cm^–2^, in porous water electrolyzer anodes with optimal loading.^118^ Despite considerable efforts in the last few decades, elucidating experimentally the details of the OER mechanism is still difficult even in “simple” systems with soluble aqueous electrolytes like KOH and with single-crystal or planar thin film electrodes. Although it is trivial to measure kinetic parameters such as the Tafel slope ζ,^118^ it is difficult to connect these parameters to specific microscopic steps of the reaction. Recent progress in computation has provided insight into the molecular OER mechanisms, particularly by coupling density functional theory approaches, including advanced solvation models with microkinetic modeling,^144^ which is strongly affected by the pH of the environment.^145–148^ In a recent study, it has been shown that pH strongly affects the oxygen evolution rate for IrO_2_ ECs (Figure 7),^149^ which has been previously shown for various first-row transition-metal oxide perovskite ECs.^150,151^

**Figure 7:**
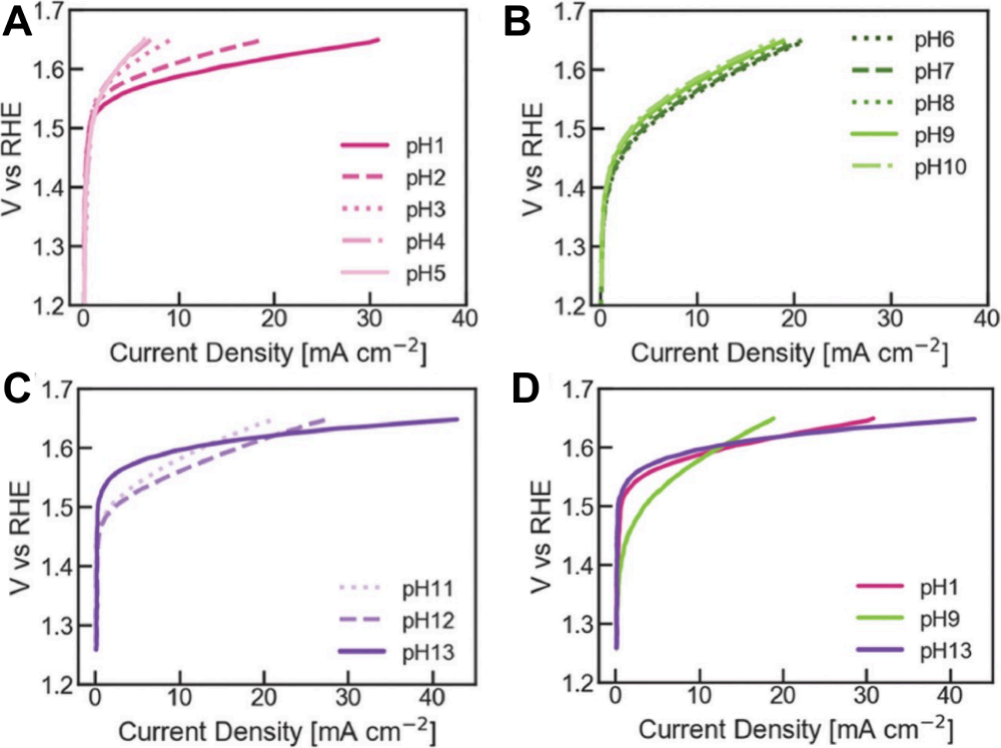
Linear-sweep voltammograms of the OER region for an IrO_2_ electrode at various pH: pH 1–5 (A), pH 6–10 (B), pH 11–13 (C) and pH 1, 9, 13 (D). Adapted with permission from ref 149. Copyright 2022, Elsevier. CC-BY 4.0 license, https://creativecommons.org/licenses/by/4.0/.

The evidence reported above indicates that the lower performance of AEM-WEs operating with deionized water, compared to AEM operated with KOH electrolytes, may in part be due to slower oxygen evolution kinetics. In deionized-water systems, while the ionomer supplies OH^–^ from the anode through the AEM, the local pH at the anode EC surface is almost certainly lower than in KOH-fed AEM electrolysis, particularly under steady-state operation. The anode active layer, thus, should be engineered with transport and local pH effects to either maximize the local pH or use pH-insensitive ECs.

Indeed, in ref 152 it was shown that significant pH differences between the anode and cathode compartments affect material corrosion and the kinetics of water splitting. This aspect is not relevant in AEM-WEs fed with highly concentrated alkali due to the buffer power of the electrolyte, but it might result in significant pH variation in AEM-WEs fed with deionized water or very low supporting alkali concentrations, where the buffer power of the electrolyte is low. Transport issues might further modulate local pH variations through the depth of the catalytic layer, thus affecting the performance of the devices. This aspect is still largely unexplored. Tools capable of determining such local pH values would be particularly relevant, providing critical information to engineer the catalytic layer.^153^

In both the neutral^154^ and the alkaline^145^ media, the OER is represented by the overall process reported in eq 5. Such a process is often assumed to be composed of four elementary steps (eqs 9–12),^146,154,155^ although other microscopic mechanisms can be hypothesized.

9 $$M+OH_{\left(aq\right)}^{-}\to M-OH+e^{-}$$

10 $$M-OH+OH_{\left(aq\right)}^{-}\to M-O+H_{2}O_{\left(l\right)}+e^{-}$$

11 $$M-O+OH_{\left(aq\right)}^{-}\to M-OOH+e^{-}$$

12 $$M-OOH+OH_{\left(aq\right)}^{-}\to O_{2\left(g\right)}↑+H_{2}O_{\left(l\right)}+e^{-}$$

M represents the active site on the “surface” of the electrode, while M–OH, M-O and M-OOH identify the species adsorbed on the active site. The term “surface” here is used loosely as it is well established that essentially all catalysts for the water oxidation layer restructure and form interphases where there is some degree of volume activity and not an atomically sharp liquid/solid boundary.

It is possible to evaluate the change in the free energy associated with each of the steps in eqs 9–12.^146^ These are indicated respectively as

(9) → ΔG_OH*_ [formation of adsorbed OH, release of a first electron];
(10) → ΔG_O*_ [conversion of an adsorbed OH into an adsorbed O, release of a second electron];
(11) → ΔG_OOH*_ [conversion of an adsorbed O into an adsorbed OOH, release of a third electron];
(12) → ΔG_O2_ [the adsorbed OOH releases one additional electron and O_2_ is evolved from the electrode surface].

The minimum “thermodynamic” overpotential of the OER in the neutral/alkaline medium is determined by the largest term among ΔG_1_, ΔG_2_, ΔG_3_ and ΔG_4_, in compliance with eq 13 ^156^

13 $$\eta^{OER}=\frac{1}{e}\underset{n=1,2,3,4}{\max}\left[\Delta G_{n}\right]-U_{0}$$

where U_0_ = ΔG_0_/4e = 1.23 V is the equilibrium potential for the OER, which is independent of pH and defined at p = 1 bar and T = 298.15 K.^157^ This is a minimum “thermodynamic” overpotential because it is set by the reaction energies of the intermediates along the reaction, not by the transition-state energies between the reaction intermediates. Experimentally the overpotential is set by the passing current, which can be observed for any nonzero overpotential with sufficiently sensitive measurement. Reaction intermediate energies are much easier to calculate than transition states and the associated kinetic barriers. Often, the Bell-Evan-Polanyi principle, where the transition-state barrier is proportional to the reaction energy, is invoked to describe trends using simplified computational approaches of the complex electrochemical process, particularly in the approaches developed and popularized by Norskov and co-workers.^158^

Using DFT to compute reaction energies, OER ECs can be distinguished by the magnitude of the different ΔG_*x*_ (x = 1, 2, 3, 4), that in turn is affected by the specific physicochemical features of the EC surface (e.g., chemical composition, structure and others).^159,160^ The magnitudes of the ΔG_*x*_ values are typically not independent of each other. Computations (without explicit solvation and with the relatively simple computational hydrogen electrode) show that in most oxide-based electrodes, a scaling relationship exists between the binding energy of HOO* and HO* on M.^161^ These computations therefore enable one to (i) propose the rate-determining step of the OER by evaluating the ΔG_*x*_ values associated with the reactions reported in eqs 6–9; and (ii) correlate those with features of the EC surface that play the most relevant role in minimizing such ΔG_*x*_ values to justify a reduced OER overpotential.^162,163^ Connecting these predictions to real experiments is challenging due to the complexity of structural changes at the surface with potential and solvation effects that are computationally difficult to account for, which may have profound differential effects on the intermediate and transition state energies. Nonetheless, calculated ΔG_OOH*_ using the simplest DFT computational hydrogen electrode approaches show a universal scaling relationship with ΔG_OH*_ ^161,164^ described by the simple equation ΔG_OOH*_ = ΔG_OH*_ + 3.2 eV. Thus, the theoretical (minimum) overpotential for an EC can be estimated with only two descriptors: ΔG_O*_ and ΔG_OH*._ The scaling law is generally accurate, at least based on computational data.^164^

Similar to the HER, volcano plots can be obtained for the OER. The first example was reported by Trasatti^142,165^ who used the heat of formation of the oxides as a descriptor for the electrocatalytic activity. Similarly, the free energies for eqs 9–12 can be used to generate a volcano plot using the descriptors ΔG_O*_– ΔG_OH*_ (for steps 2 and 3) and ΔG_OH*_ (for steps 1 and 4). The resulting two-dimensional plot shows that the most promising ECs stay in the red zone in the middle of the diagram, where the minimum overpotential is of the order of 0.4 eV, with deviation from the scaling relationships that may be as large as ± 0.1 eV (Figure 8).^142^ Significant information from the plot is that a relevant number of ECs range in the ± 0.1 eV within the peak and suggests a significant issue that must be overcome to increase electrolyzer performance. Considering an electrolyzer operation at 2 V, the OER kinetic overpotential accounts for ∼ 20% of the electrical energy input. In Figure 8, the computational volcano plot can be divided into four regions that correspond to different steps that are rate-controlling. Among the considered ECs, most materials have the adsorbed hydroxyl oxidation to adsorbed oxygen and the oxidation of adsorbed oxygen to OOH* is predicted to be rate-determining based on this simple model.^142^

**Figure 8:**
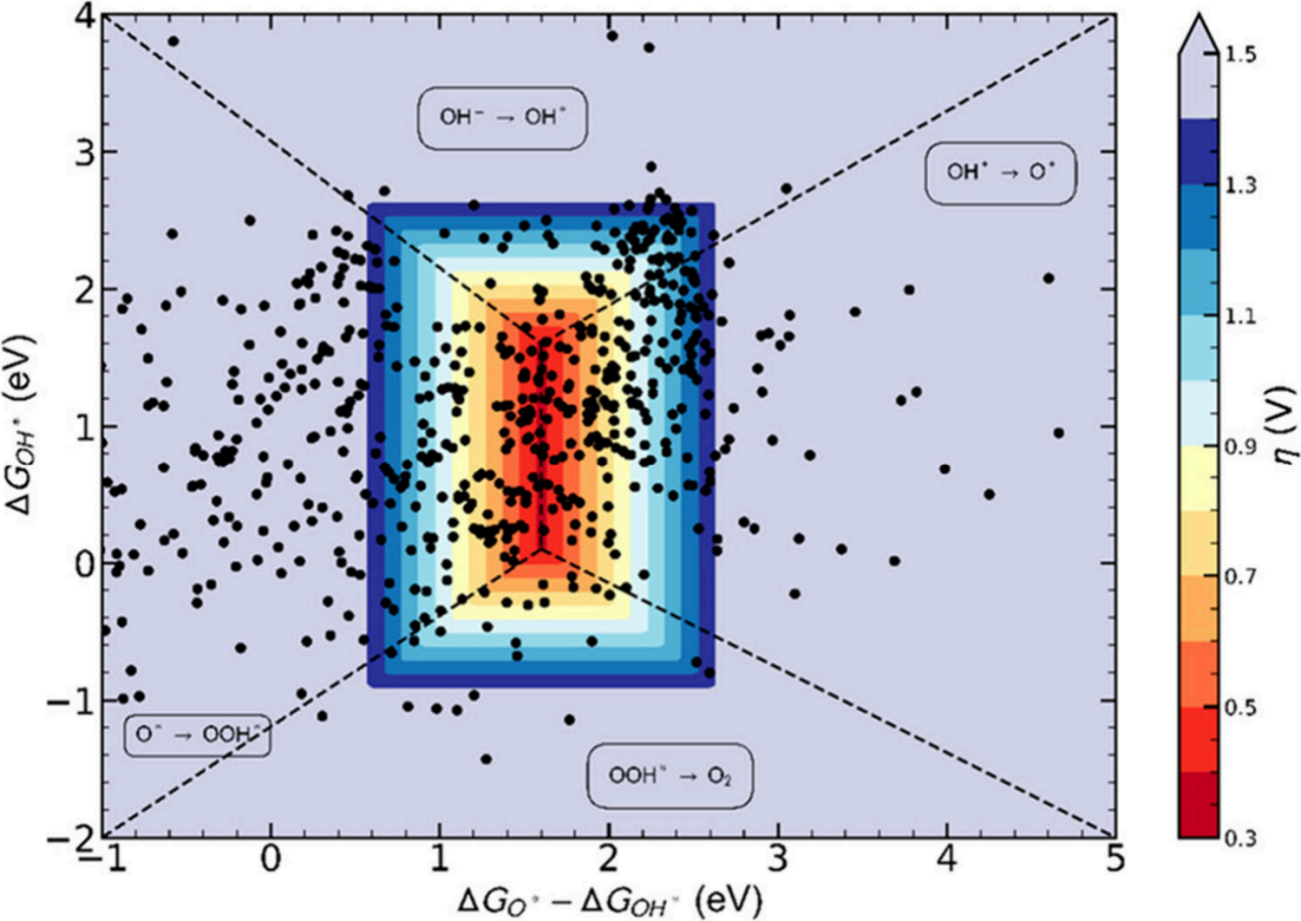
Volcano plot for the OER plotted using the ΔG_O*_ – ΔG_OH*_ and ΔG_OH*_ descriptors. Adapted from ref 142. Copyright 2022, American Chemical Society. Licensed under CC-BY-NC-ND 4.0.

While these calculated thermodynamic descriptors provide useful insight, the connection to experimental systems can be tenuous. For example, alkaline solutions are often contaminated with Fe impurities, the oxide surfaces are reconstructed to form disordered surface-oxyhydroxide phases, which do not have the same properties as the underlying oxides and the dramatic differences in electrical conductivity across oxide and oxyhydroxide phases are not accounted for in these models. The intrinsic activity trends of these transition metal-based oxyhydroxide-type phases (which are derived often from parent oxides) have more recently been reconsidered taking these issues into account,^166^ but a mechanistic understanding in the context of thermodynamic intermediate energies, particularly for the fastest ECs that are multicomponent phases like Ni­(Fe)­OOH and Co­(Fe)­OOH (discussed more below) is still unsettled.^167^ Craig et al. showed that inhomogeneous systems in this descriptor fail to predict the electrocatalytic behavior of Ru ECs with a significant underestimation of the activity. In these cases, it is relevant to consider the variation of the oxidation state of the metal site, an element that poses serious issues in the calculation.^168^ There is still a need to significantly improve the current interpretation framework to rationalize and predict the activity through accurate descriptors and the resulting plots. However, it is not yet clear whether fundamental limitations in the knowledge faced today can be surpassed effectively in the future.

Besides the complex theoretical framework, there are also issues with the experimental methods. Particularly, the preparation methods of the electrodes and the need for thick EC layers create significant difficulties in the definition of active surface areas, the electrical conductivity of the layer and the local surface structure. The uncontrolled experimental condition provides uncertainty in the normalization of the data and the comparison of the intrinsic OER activity of materials. The introduction of fabrication methods that will allow precise control of the EC surface and condition is recommended. Such methods would allow an easy determination of the turnover frequency, which is a good metric for benchmarking the various materials.^169^

## 5 Materials and Their Integration

A single-cell AEM-WE is composed of several single components that are integrated into the membrane electrode assembly (MEA) that is the core of the system, as presented in Figure 9, in an AEM-WE operating with “dry-cathode”.^170^ The AEM is at the center of the AEM-WE. On the anode side, where the OER takes place, a catalytic layer (CL) composed of the EC and the AEI is in contact with the AEM on one side and on the other side with the PTL. The anodic BP closes the anode side. Similarly, on the cathode side where the HER occurs, a CL composed by the EC and the AEI is between the AEM and the cathode PTL. At the end of the cathode side, there is the BP. The CL can be integrated into the AEM or the PTL as EC Coated Membrane (CCM) or as EC Coated Substrate (CCS), respectively. In the next subsection, single components and their integrations in MEAs are discussed.

**Figure 9:**
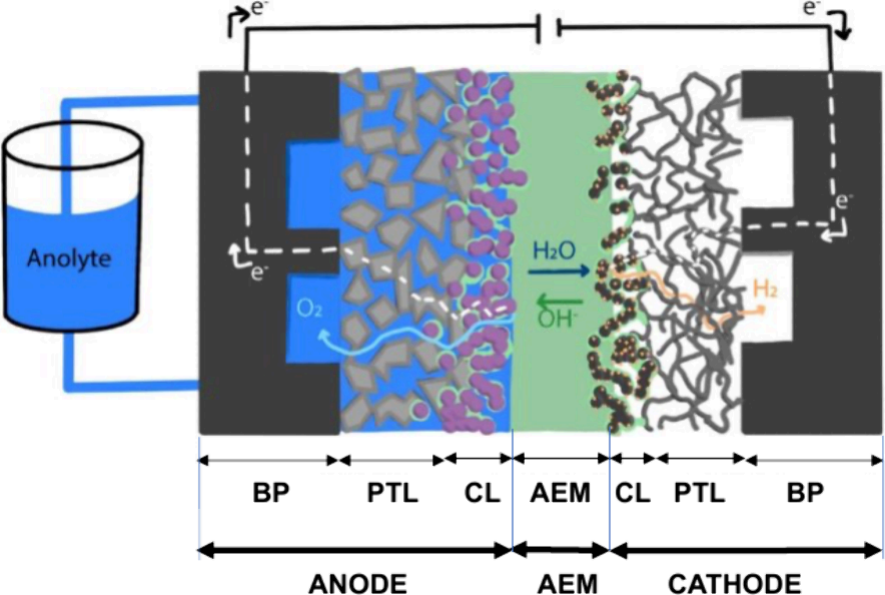
Schematic of a single cell AEM-WE including the single components and their integration. Adapted from ref 170. Copyright 2021, IOP Publishing Limited. Licensed under CC-BY 4.0.

### 5.1 Electrocatalysts for the Hydrogen Evolution Reaction

As discussed above in section 4.2.1, the HER in the alkaline medium is hindered by the water dissociation process. This is a significant difference with respect to the acidic medium. Consequently, even ECs exhibiting excellent performance in the acidic medium could experience performance decline in the alkaline medium. It is generally believed that platinum-supported carbon (Pt/C) is the best HER EC both in acidic and alkaline media. This is a consequence of the optimal compromise between facile adsorption of the water reagent and an unhindered release of the H_2_ product.^171^ The other PGMs (e.g., Pd, see Figure 5.B) also exhibit a good HER performance in the alkaline environment. The low abundance of PGMs in Earth’s crust^172^ challenges the scaling of the technology implementing high loadings of PGMs.^173^ However, the use of cathodes with a low loading of PGMs (e.g., near or below 0.1 mg cm^–2^) may be tolerable at scale, as recently mentioned in the publication from the US Department of Energy Hydrogen and Fuel Cell Technologies Office.^174^

HER ECs for operation in alkaline media based on Pt have been extensively studied and implemented in AEM-WEs. ECs, including active sites only comprising Pt, yielded good performance in operating conditions in AEM-WEs fed with both pure water^56^ and alkaline electrolyte.^175^ With respect to Ni-based HER ECs (see below), the improved efficiency leads to a lower consumption of energy upon AEM-WE operation. This offsets the higher cost of the Pt-based HER EC. The HER activity of Pt/C is improved upon the combination with layered metal hydroxides; the latter are supposed to enhance the dissociation of water and the production of hydrogen intermediates.^127,176^ A number of bimetallic Pt-based HER ECs were studied as well. The second metal promotes the kinetics of the HER either: (i) by facilitating the cleavage of the HO-H bond (e.g., in the case of PtNi^177^ and PtCo^178^); or (ii) by decreasing the Pt–H* binding energy (e.g., in the case of PtRh^179^ and PtRu^180^). HER ECs comprising PtRu active sites are particularly promising and have demonstrated their potential to devise AEM-WEs exhibiting an outstanding performance. For instance, an AEM-WE mounting a NiFe anode EC, a PtRu/C cathode EC, a HTMA-DAPP AEM and fed with 1 M NaOH at 60 °C was able to operate at 5.32 A cm^–2^ at a cell voltage of 1.8 V.^181^

Ni-based ECs are promising alternatives to PGMs to promote the HER, since they exhibit reasonable activity and a significant abundance in Earth’s crust.^182^ Several approaches were adopted to reduce the size of Ni particles composing the electrode EC and increase the interfacial area.^183,184^ Raney Ni ECs demonstrate a high activity in the HER thanks to the porous morphology and the large surface area obtained during the synthetic process.^185^ Further improvements were achieved by developing different Ni alloys such as Raney Ni–Sn EC (Figure 10.A).^186,187^ In these studies, a reduction in the Tafel slope from 120 to 70 mV dec^–1^ was achieved with the Sn-added Raney Ni ECs. Other binary and ternary Ni alloys were explored, including Ni–Co, Ni–Fe, Ni–Cr, Ni–Mo–Fe, Ni–Mo–Cu and Ni–Mo–Co, taking advantage of the good lattice match of Ni with other metals.^188–190^ Among such alloys, Ni–Mo systems yielded an overpotential of only 35 mV at a current density of 10 mA cm^–2^ and a Tafel slope of 45 mV dec^–1^, which is comparable to the performance of a commercial Pt/C EC,^187,191^ albeit with much higher mass loading for the Ni–Mo system. Ni–Mo HER ECs are highly promising as they have demonstrated remarkable performance in single AEM-WE.^53^ With respect to a reference AEM-WE mounting a conventional PtRu/C HER EC, an AEM-WE comprising a nanocomposite Ni–Mo HER EC supported on oxidized Vulcan carbon black yielded only a 50–100 mV higher cell voltage at geometric current densities of 1 A cm^–2^ or higher.^53^ This latter Ni–Mo EC exhibited: (i) a mass-specific HER activity within 1 order of magnitude of a commercial Pt/C EC; and (ii) significant internal mass transfer limitations even at loadings as low as few tens of μg_cat_ cm^–2^, suggesting that the above results are lower-bound estimates of the intrinsic HER/HOR activity of Ni–Mo composites. The remarkable performance of such Ni–Mo EC was rationalized on the basis of DFT calculations, suggesting that the introduction of Mo in Ni–Mo alloys weakens hydrogen adsorption and raises HER activity.^53^ Additional studies are still needed to improve durability and elucidate failure modes under HER conditions, thus reaping the full benefits of Ni–Mo HER ECs for the alkaline environment.

**Figure 10:**
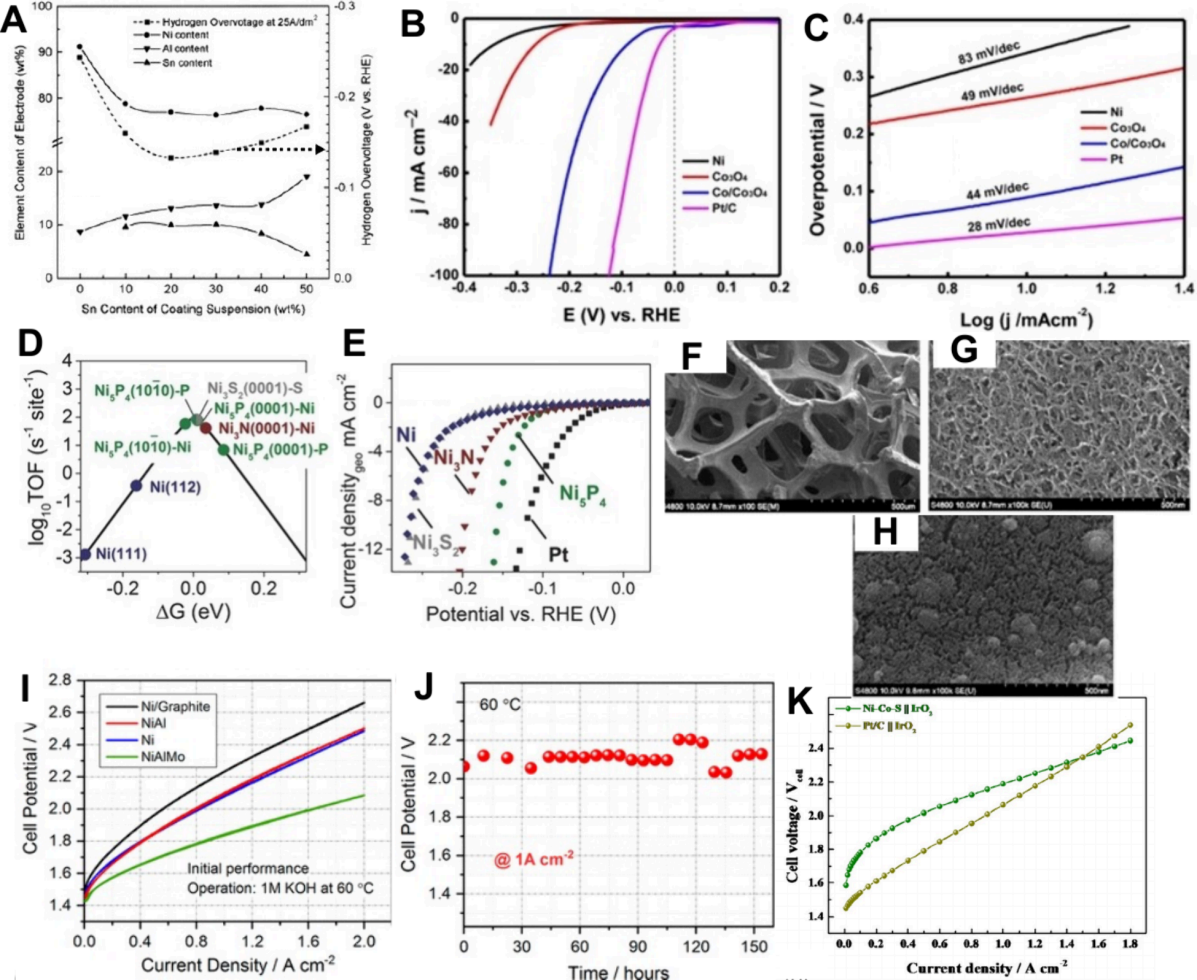
(A) Impact of the chemical composition of a Raney-Ni electrode on the HER overpotential at 25 A/dm^2^ at 25 °C. Reprinted with permission from ref 186. Copyright 2000, Elsevier. Published by Elsevier Science Limited. (B) Polarization curves of bare Ni foam, Co_3_O_4_ nanosheets, Co/Co_3_O_4_ nanosheets, and Pt wire. (C) Tafel plots derived from (B). (B) and (C) adapted with permission from ref 192. Copyright 2015, American Chemical Society. (D) Position of the different crystal surfaces of Ni_*x*_M_*y*_ (M = P, S, N) in the HER activity volcano plot as a function of the calculated hydrogen adsorption-free energies. (E) “*Ex-situ*”current–voltage characteristics of Pt, Ni_5_P_4_, Ni_3_N, Ni_3_S_2_, and Ni working in 1 M KOH. (D) and (E) were adapted with permission from ref 193. Copyright 2016, WILEY-VCH Verlag GmbH. Morphology of HER ECs: (F) nickel foam; (G) detail of the surface of a branch; (H) surface of a branch after deposition of Ni­(OH)_2_, adapted with permission from ref 194. Copyright 2008, The Royal Society of Chemistry. (I) AEM electrolyzer cell performance at 60 °C using NiAlMo cathode and different anodes: Ni/graphite, NiAl, Ni, and NiAlMo; (J) durability test of the cell using NiAl anode for about 154 h under a current density of 1 A cm^–2^, reprinted with permission from ref 195. Copyright 2019, American Chemical Society. (K) Polarization curves for AEM electrolyzer single cells with Ni–Co–S/CP and commercial Pt/C/CP cathodes, reproduced with permission from ref 196. Copyright 2020, John Wiley & Sons Limited.

Metal oxides by themselves are not very active in promoting the HER due to their poor electrical conductivity and hydrogen binding energies. However, when metal nanoparticles are at least partially covered by suitable metal oxides, such as in the case of Co/Co_3_O_4_, Ni/NiO and Ni/CeO_2_–CNT systems,^192,197,198^ bifunctional effects are likely triggered, yielding a remarkable HER performance. Ni and NiO typically coexist on the surface of Ni-based electrodes (e.g., nickel foams).^199^ The presence of NiO on the surface of a Ni electrode significantly raises the intrinsic kinetics of the HER in an alkaline environment. This evidence was interpreted assuming that the introduction of NiO raises the free energy of the adsorbed hydrogen intermediate and enhances the kinetics of the Volmer step.^200^ Indeed, a lower overpotential in the HER was demonstrated for these heterostructured ECs with respect to the single metal or metal oxide systems (Figure 10.B and Figure 10.C).^192^ Similarly, Ni/CeO_2_–CNT ECs demonstrated an overpotential of *ca*. 100 mV at a current density of 10 mA cm^–2^.^198^ The introduction of heteroatoms such as S, N and P forming active sites based on transition metal sulfides, nitrides and phosphides gives rise to an enhancement of the HER activity of the EC. In particular, it was demonstrated that these heteroatoms are not just spectators and play a crucial role in the HER mechanism (Figure 10.D).^193^ In the presence of such heteroatoms, the hydrogen adsorption-free energy approaches zero. This latter feature is strongly correlated with a high HER activity.^107^ A few examples of such ECs include systems based on Ni_3_S_2_, Ni_3_N, Ni_5_P_4_, CoP, CoS_*x*_, MoP, MoS_*x*_ and WP_*x*_ (Figure 10.E)).^193,201–204^ In this group of ECs, the best performance in *“ex-situ”* tests was obtained by devising nanocrystalline CoP nanosheets supported on carbon cloth, which: (i) were able to activate the HER with an overpotential of 48 mV at a current density of 10 mA cm^–2^; and (ii) exhibit a Tafel slope of 43 mV/decade.^201^

#### 5.1.1 HER Electrocatalyst for AEM-WEs and Their Differences with Respect to A-WEs

The key distinction between A-WEs and AEM-WEs lies in the electrolyte and the operating conditions, such as the nature of feed^205^ and lower operational temperature, along with the possibility of pressurizing H_2_ within the WE.^20^ Thus, the choice of HER EC depends on the specific type of WE and its compatibility with the operating conditions.^205,206^ Compared to PEM-WEs, an advantage of AEM-WEs is the possibility to implement lower-cost TM in HER ECs that would be unstable in acid.^59^ In addition, the three-dimensional design of the porous electrode is a crucial factor in ECs for AEM-WEs: nickel foam (NF), graphene (G), graphene oxide (GO) and carbon nanotubes (NTs) are often used supports for the preparation of MEAs for AEM-WEs in academic laboratories. The introduction of such supports enhances the porosity, raises the electrochemically active surface area (ECSA) and facilitates gas desorption (see Figure 10.F, Figure 10.G and Figure 10.H).^194^ These supports often provide high electrical conductivity, thus minimizing ohmic losses. However, there are design trade-offs. Excessively thick EC layers, even if they provide high surface area, can lead to higher electrical, ionic, and mass transfer resistances that ultimately decrease cell performance.^207^

AEM-WEs and A-WEs usually adopt HER ECs with the same or similar chemical composition as they both operate in an alkaline environment.^205^ Typically, PGM-free ECs for HER are based on Ni. For instance, Ni–Mo alloy EC demonstrated a good performance in full-cell at 50 °C, achieving a current of 1 A cm^–2^ at 1.9 V.^208^ Alloys of nickel with cobalt supported on GO were tested at room temperature, revealing a decent performance of 100 mA cm^–2^ at 1.9 V.^209^ In addition to binary alloys, ternary Ni alloys were developed, such as NiAlMo and NiFeCo.^195,210^ Both performed well when in a full AEM-WE operating at 60 °C and fed with 1 M KOH, achieving a current of 2 and 1 A cm^–2^ at 2.09 and 1.90 V, respectively (see Figure 10.I and Figure 10.J).^195,210^ The AEM-WEs, including the NiAlMo and NiFeCo HER ECs, mounted a hexamethyl-p-terphenyl poly-(benzimidazolium) (HMT-PBI) AEM and a PBI-based AEM, respectively.

Sulfide-, oxide- and nitride-based HER ECs were also developed. A Co_3_S_4_ EC showed a high current density of 0.43 A cm^–2^ with a cell voltage of 2 V,^211^ while a Ni–Co–S EC exhibited a current density of 1.7 A cm^–2^ at 2.4 V (see Figure 10.K).^196^ A NiCu mixed metal oxide EC demonstrated an AEM-WE performance of 1.85 A cm^–2^ at 2 V in 1 M KOH at 50 °C.^207^ Finally, an HER EC named as “NiMo-NH_3_/H_2_”, including a NiMoN_*x*_ component and obtained by annealing NiMoO_4_ on nickel foam (NF) in H_2_/NH_3_ (5% H_2_/ 95% NH_3_) for 2 h at 550 °C achieved a current density of 1 A cm^–2^ with an applied potential as low as 1.57 V.^212^ It is highlighted that the literature rarely reports the chemical composition of HER ECs after operation. Hence, a possible selective leaching of species (e.g., Al, Cu) from the ECs upon carrying out the HER is not probed. This hinders a detailed understanding of the real chemical composition of the active sites and, consequently, of the actual HER mechanism and its interplay with the physicochemical properties of the EC.

### 5.2 Electrocatalysts for Oxygen Evolution Reaction

In an alkaline medium, in principle, a broad spectrum of metal elements can be used to produce ECs to promote the OER (Figure 11.A). The elements span from PGMs (e.g., Pt, Ir, Ru)^213^ to first-row TMs, particularly Fe, Co, Mn and Ni.^214^

**Figure 11:**
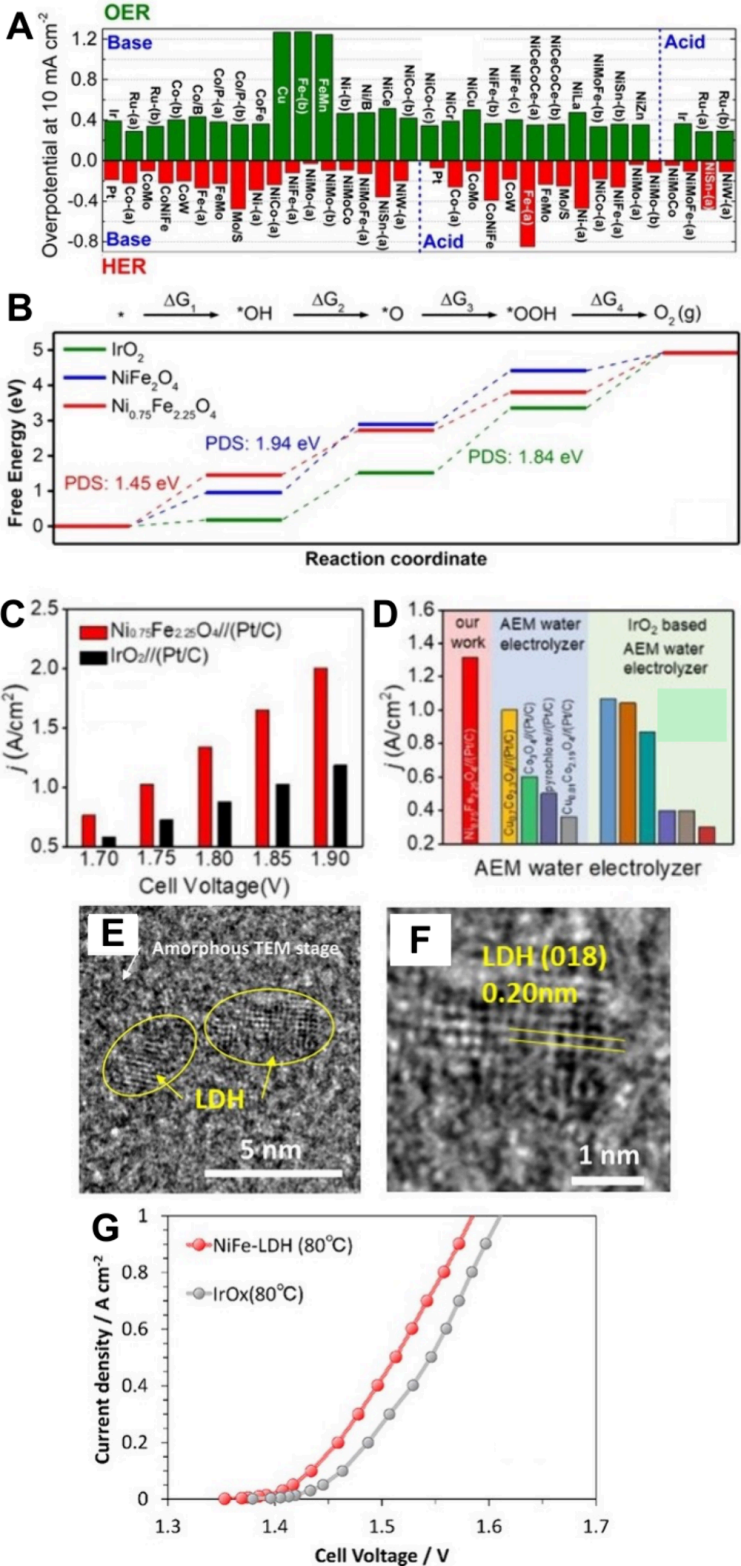
(A) Activity of various OER ECs (ECs) after 2 h of operation at 10 mA·cm^–2^, reprinted with permission from ref 214. Copyright 2015, American Chemical Society. (B) Free energies of OER intermediates on the IrO_2_(1 1 0), NiFe_2_O_4_(3 1 1) and Ni_0.75_Fe_2.25_O_4_(3 1 1) surfaces at U = 0 V. PDS = potential-determining step. (C) Current density at various cell voltages for Ni_0.75_Fe_2.25_O_4_//(Pt/C) and IrO_2_//(Pt/C) cells. (D) Comparison of AEM-WE for the Ni_0.75_Fe_2.25_O_4_//(Pt/C) cell, other reported AEM-WE cells, and the reported IrO_2_-based AEM-WE. (B–D) were reproduced with permission from ref 232. Copyright 2020, Elsevier B.V. (E, F) High-resolution TEM image of NiFe layered double hydroxide (NiFe-LDH) OER EC. (G) Current–voltage curves obtained for MEAs using (red) NiFe-LDH and (gray) IrO_*x*_ at 80 °C as an anode EC. (E–G) were reproduced with permission from ref 233. Copyright 2020, American Chemical Society.

With respect to PGMs, the activity trend is nominally Pt < Ir < Ru,^213^ although the Ru and related oxides dissolve under anodic polarization in basic electrolyte. The combination of different PGMs can trigger synergistic effects, raising OER performance and durability. For instance, doping RuO_2_ with Ir to yield Ru_0.9_Ir_0.1_O_2_ significantly raises the durability without compromising the OER performance.^215^

OER ECs based on non-noble metals, in fact, have intrinsically higher OER activity than PGMs^216^ and can be infinitely stable/durable, often due to dynamic dissolution-redeposition processes,^217^ particularly for Fe-containing Co/Ni ECs with record activity.^205,214^ This is a primary advantage of A-WEs and AEM-WEs over systems operating at lower temperatures in acidic conditions (e.g., PEM-WEs). At comparable energy conversion efficiencies, the operating current density increases significantly from A-WEs (*ca*. 0.5–1 A cm^–2^)^205^ to PEM-WEs (*ca*. 2–4 A cm^–2^), resulting in more compact devices.^218^ However, this high-performance level is only achieved by PEM-WEs whose electrodes are based on PGMs such as Pt, Ir and Ru.^218^

Over the past 50 years, there have been substantial efforts to develop high-performing ECs able to promote the OER based on earth-abundant elements (especially Fe, Co and Ni; other elements were also investigated, such as Ti, Mn and Pb).^161^ A *“baseline”* OER EC for the alkaline environment is based on Ni or highly porous Raney Ni deposited/applied on a stainless steel current collector.^219,220^ At their surface, these ECs are transformed in the active phases, nominally consisting of Ni­(Fe)­OOH. The thickness and local structure of this dynamically formed active phase, along with the surface area of the initial porous metal electrode, determine the ultimate operating electrode potential as a function of current.^221^ Several researchers have synthesized and tested various metal oxides of Ni, Co, and Fe with other elements added, including Zn, Cu, Al, and many others.^215,222^ In these cases, the active surface phases are always the disordered Ni or Co-based oxyhydroxides with Fe incorporated either intentionally or unintentionally from electrolyte impurities.^223,224^ Elements like Zn, Cu, and Al have been invoked to enhance activity, which is due to their solubility; these elements leach during operation, leading to a faster formation of the active phases discussed above with a higher degree of (initial) local disorder. A huge variety of initial precatalyst materials, including metals, alloys, oxides, phosphates, phosphides, sulfides, selenides and nitrides, have been reported and the resulting materials were all claimed to be able to yield an excellent OER performance.^225,226^ This is because the real active sites for the OER are the oxyhydroxides, almost uniformly containing Ni and/or Co along with Fe, that are formed during the electrochemical oxidation of water.^227,228^ This is true regardless of the chemical composition of the parent material (pure metal, alloy, or compound). NiFe alloys, including steel, can be used as parent materials to obtain OER electrodes.^229,230^ Upon suitable treatment (e.g., dealloying), highly active and stable electrodes can be obtained.^229,231^ During operation, the exsolution of the bulk components is known to further activate the OER electrode and yield a more stable system in comparison with heterodeposited active layers. Furthermore, such a system may self-heal during operation.^229,231^ The features of the parent material and the subsequent surface treatments, if any, are the avenues by which the physicochemical properties of the oxide and oxyhydroxide active sites formed *operando* on the EC are achieved. This modulates the apparent performance and durability in the OER of the EC, along with cell engineering parameters as discussed in more detail below.

#### 5.2.1 OER Electrocatalyst for AEM-WEs and Their Differences with Respect to A-WEs

Similarly to A-WEs, AEM-WEs can run with PGM-free OER ECs and exhibit a performance high enough for practical applications.^195^ At the same time, the baseline OER ECs used in academia for AEM-WEs are still based on PGMs such as Ru and, more commonly, Ir (typically as IrO_*x*_),^56,232,234^ though it would probably be more appropriate to adopt other OER benchmark ECs, including Co_3_O_4_ or other oxides.^19^

A first family of promising PGM-free OER ECs comprises active sites based on Ni, usually coupled with other transition elements such as Fe (Figure 11.(A-D))^232^ and/or Co^235^ similar to other OER ECs designed for A-WEs.^223,224^ The performance of PGM-free OER ECs in an operating AEM-WE is often superior to that afforded by baseline IrO_*x*_ ECs.^233^ For example, in 1 M KOH and at T = 80 °C, an AEM-WE with a NiFe layered double hydroxide (LDH) operated at 1.59 V at 1 A cm^–2^. In the same conditions, IrO_*x*_ EC operated at 1.61 V.^233^ It is also feasible to devise entirely PGM-free AEM-WEs. One such device was configured with either a binary NiFe hydroxide or a ternary NiFeCo hydroxide OER EC, coupled with a Ni mesh as the cathode.^235^ The resulting AEM-WE yielded 500 mA cm^–2^ at 2.22 V in 1 M NaOH; such a result was obtained with a ternary NiFeCo hydroxide OER EC wherein the Ni:Fe: Co molar% ratio was 23:8:69. Co-based OER ECs also yielded very good performance. Some examples include bimetallic OER ECs wherein CoFe nanoparticles (Co: Fe molar ratio: 0.9/0.1) were deposited on highly graphitized carbon nanofibers (H–Co_0.9_Fe_0.1_-CNF).^236^ The corresponding AEM-WE achieved a current density of 0.794 A cm^–2^ at 1.7 V. Bimetallic Cu_0.5_Co_2.5_O_4_ OER ECs were synthesized by coprecipitation at pH = 11 of the corresponding precursors. The AEM-WE using Cu_0.5_Co_2.5_O_4_ OER EC yielded a current density of 1.3 A cm^–2^ at 1.8 V.^237^ Several other combinations of metals can be introduced in OER ECs; one example is a Ni/CeO_2_–La_2_O_3_/C system.^206^ The main differences between OER ECs for A-WEs and AEM-WEs are associated with the features of the interfaces between the electrode configurations and the separator in the two families of devices. In conventional A-WEs, the OER EC is fabricated onto a porous current collector by a variety of approaches, including electrodeposition^238^ and plasma spraying.^239^ As discussed in detail below, it is also important to distinguish between AEM-WE results where the water feed is alkaline (e.g., 0.1 M KOH) or pure water, as this affects the suitable EC properties.

On the other hand, in AEM-WEs, massive efforts are devoted to obtain highly refined “three-phase boundaries” (TPBs) on the active sites of the ECs at the interfaces between each electrode configuration and the AEM, able to: (i) minimize the sources of overpotential not associated with the intrinsic electrochemical kinetics of either the OER and the HER, and (ii) achieve a sufficient AEM-WE durability.^206^ To achieve this goal, in AEM-WEs, the electrode configurations and the AEM are fused together to obtain the MEA.^205^ In an MEA, the anodic and cathodic TPBs are very close to one another on the opposite faces of the AEM, allowing for to minimization of the ohmic losses due to the migration of OH^–^ species through the membrane and achieving the high operation current density characterizing AEM-WEs.^205,206^

To reduce the overpotentials due to mass transport, the OER ECs for AEM-WEs are to be supported on highly porous matrices allowing for a facile expulsion of the O_2_ bubbles obtained as the product; a typical example is nickel nanofoam (NiNF).^240^ Other possibilities include substoichiometric metal-oxides, carbides, and nitrides, which are expected to retain their porous structure even under the high oxidative potentials found at the AEM-WE anode.^241^ A high AEM-WE performance is also promoted by other morphological features beyond porosity, such as the surface area and the shape of the OER EC, which ensures the access of ions to the active sites. Sample morphologies include nanoparticles,^242^ nanoflowers,^243^ nanorods,^244^ and many others. Another crucial condition to achieve a high AEM-WE performance is to maximize the electrical conductivity of the OER EC (or anode active layer), to curtail the losses associated with charge transport. One possibility to achieve this outcome is to enhance the contact between the surface of the electrode and the OER EC; this result is often achieved by the direct growth (e.g., by electrodeposition) of the EC on the electrode surface.^240^ Another approach involves the introduction of carbon-based materials in the OER EC; good results are claimed, especially upon doping such carbon-based materials (e.g., graphene) with nitrogen moieties. Pyridinic N and graphitic N are claimed to raise charge delocalization, promoting the OER.^245^ It is to be highlighted that OER operation takes place at high electrode potentials (typically, E > 1.5 V). In these conditions, carbonaceous species are bound to undergo corrosion.^246^ On these bases, it must be stressed that even though carbon-supported ECs for the OER are widely studied in the scientific literature for fundamental purposes, they stand little chance of achieving in realistic conditions the durability level that is necessary for practical applications.

One key difference between A-WEs and AEM-WEs is the level of durability demonstrated in practical operating conditions. A-WEs, after decades of improvements and refinements, exhibit a durability often exceeding 100,000 h.^205,206^ On the other hand, the practical lifetime of AEM-WEs is not well-defined, especially beyond the 10,000 h limit and with intermittent operation.^206^ Intermittency and variable load operation are common if the electrolyzer is run by electricity obtained from renewable sources such as the sun and the wind and in response to electrical price signals.^205^ AEM-WEs must exhibit a 40,000 h lifetime to compete successfully with PEM-WEs.^26^ The main reason underlying this discrepancy between A-WEs and AEM-WEs is that the latter technology is still under research and development and is not as technologically mature. Hence, in most instances, there are no practical conditions to carry out an extensive 100,000 h durability test on an AEM-WE including developmental components. Most durability studies on AEM-WE components, such as OER ECs, are carried out through accelerated aging tests, fixing either the operating potential or the current density (10 mA cm^–2^ < *j* < 1000 mA cm^–2^) for limited timespans up to several hundred hours.^210^ Another possibility is to implement cyclic voltammetry durability tests within high and low potential limits for several hundred cycles (>1000 cycles).^247^

A variety of features of the OER EC are used to reveal signs of degradation. The latter include: (i) morphological changes; (ii) compositional changes; or (iii) changes to the oxidation states of surface elements that may affect the OER activity. For instance, in nonoxide OER ECs the nonmetallic element (e.g., P in FeNiCoP systems) was shown to dissolve upon AEM-WE operation.^248^ The oxidation state of Mn could rise as well, increasing charge transfer resistance and negatively affecting the performance of the MEA.^244^ There are several additional factors negatively influencing the durability of AEM-WEs, but most are related to other components of the MEA (e.g., the binder, the AEM, the fabrication approach) and not to the intrinsic features of the OER EC. However, the OER EC and the electrode must remain in good contact during AEM-WE operation. Indeed, EC detachment is well-known to be a major source of performance loss in AEM-WEs.^206^ Such a detachment is easily triggered by the release of gas bubbles during AEM-WE operation, especially at high current densities (Figure 11.E-G).^233,249^ In addition, the formation of bubbles effectively raises the electrode resistance simply by inhibiting the access of the electrolyte to the surface of the OER EC.^244^ One way to address this shortcoming could be the enhancement of the OER EC porosity and hydrophilicity, to promote the expulsion of bubbles.^250,251^

### 5.3 Impact of Membrane Electrode Assembly on AEM-WE Performance

Despite the progress in the development of individual components of the MEA, such as ECs, membranes and ionomers, the lack of careful integration of such may hinder the improvement of AEM-WE performance. This is particularly important in AEM-WE operated with pure water, since an extended interface between the electrocatalyst and the solid polymer electrolyte in both the anode and cathode catalyst layers is essential to maximize the reaction rates. Conversely, in the presence of a supporting electrolyte solution (e.g., KOH), the whole wetted EC surface constitutes the EC-electrolyte interface.^252^ In this case, the ionomer is not crucial for OH^–^ conduction, and it acts solely as a binder.

#### 5.3.1 Role of the Membrane

AEMs typically consist of a polymer backbone with cationic anchoring groups that provide anion conductivity and selectivity. The hydrocarbon polymeric backbone commonly uses polysulfone or polystyrene to connect divinylbenzene, and specific ion exchange groups involving N-based groups,^20^ with piperidinium^255^ and spirocyclic^256^ being currently state-of-the-art. New ion exchange groups with great potential to achieve improved alkaline stability were recently reported.^257–265^ Multiple rigorous reviews on the research advancements regarding the polymer chemistry of AEMs are already available and are therefore not the focus of this review.^68,266^ Figure 12 presents a schematic representation of an AEM based on a quaternary ammonium pendant functional group and the chemical structures of selected commercial AEMs (Sustainion by Dioxide Materials, PiperION by Versogen and Aemion by Ionomr) adapted from refs 253 and 254. Table 2 summarizes key performance metrics for these AEMs. This comparison highlights varying characteristics and performance parameters, noting that specific performance differences may depend on application and testing conditions.^267^

**Figure 12:**
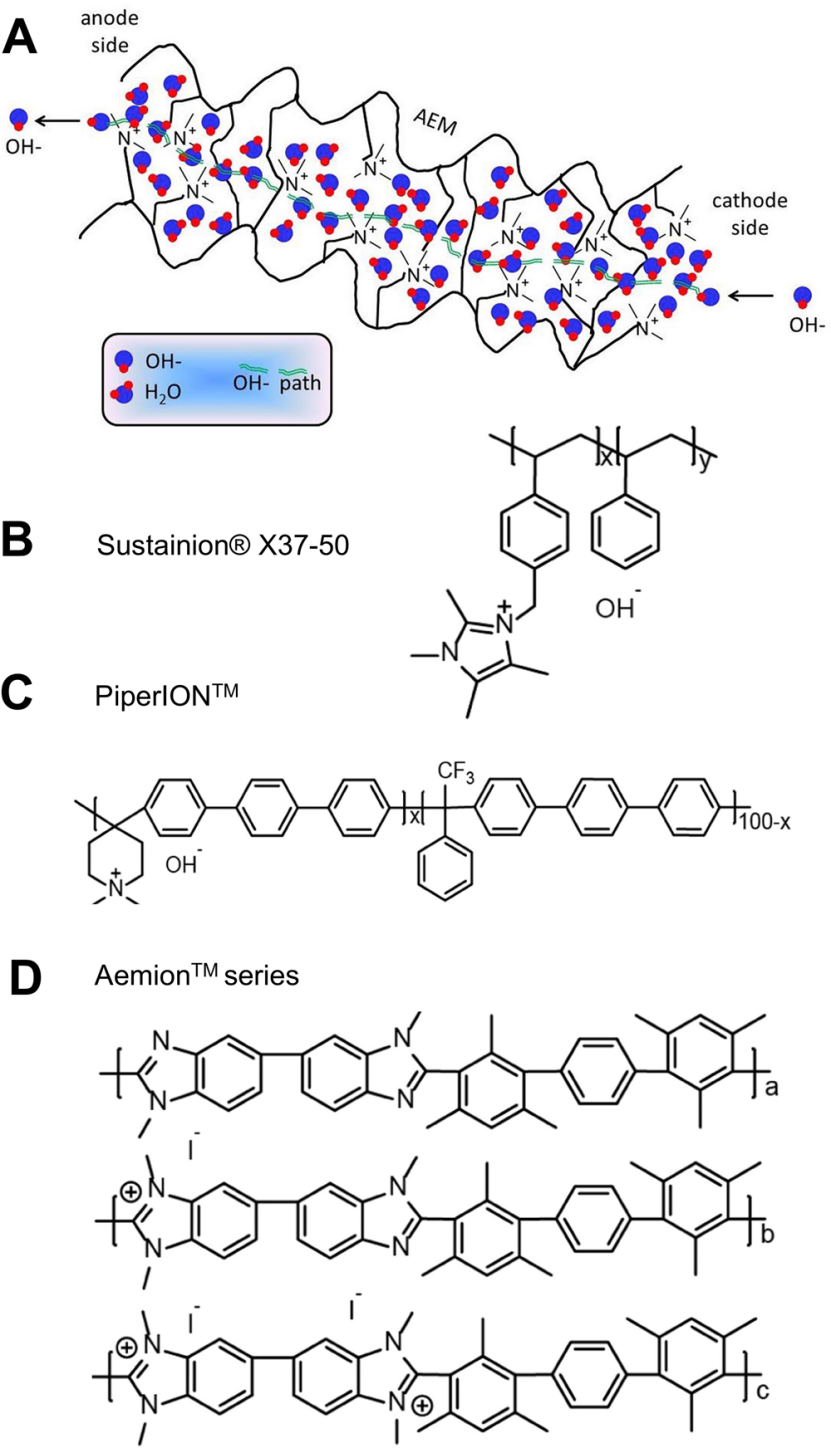
Schematic representation of an AEM based on a quaternary ammonium pendant functional group (A). Adapted from ref 253. Copyright 2017, Elsevier. Licensed under the CC BY-NC-ND 4.0. Chemical structures of selected commercial AEMs such as Sustainion X37-50 (B), PiperION (C) and Aemion series (D) adapted with permission from ref 254. Copyright 2022, American Chemical Society.

**Table 2:** Comparison of Commercial AEM Properties^a^

Parameter	[-]	Sustainion X37-50	PiperION	Aemion:
IEC	meq g^–1^	1–1.25	2.1–2.3	1.4–2.7
Conductivity	mS cm^–1^	140 at 80 °C	149 at 80 °C	130 at 50 °C
Thickness	μm	50	20–80	25–75
Tensile strength	MPa	21	21–60	53–60
Elongation break	%	52	52–390	42–110
ASR	Ω or Ω cm^–2^	0.3 Ω at RT	0.35 Ω at RT	0.065–0.68 at 60 °C
		0.045 at 60 °C		
Water uptake	wt %	83 @ RT	54 @ 80 °C	13–40 @ RT
Swelling ratio	%	15.1 @ RT	8 @ 80 °C	9–18 @ RT
Chemical stability		n.a	60%–80% OH- conductivity reduction after 4 weeks in 1 M KOH at 60 °C.	39%–72% Cl conductivity reduction after 7 days in 3 M KOH at 80 °C.

a Data were taken from ref 267. Elsevier, Licensed CC BY-NC 4.0.

The AEI is cast to form the AEM, which is responsible for transporting OH^–^ from the cathode to the anode electrode and maintaining necessary water hydration at both electrodes while preventing gas crossover. Historically, AEMs have suffered from low ionic conductivity and chemical stability, which hinder the performance and durability of AEM-WE systems compared to PEM-WE systems when fed with pure water. The conductivity of OH^–^ in AEMs is significantly lower than that of H^+^ in PEMs. Consequently, for membranes with the same thickness, the AEM-WE system experiences considerable voltage losses from ionic conduction through the membrane compared to PEM-WE when no OH^–^-containing supporting electrolyte is used. To improve hydroxide conductivity to compete with PEM-WE, the number of cationic groups in AEMs should be increased; however, this can lead to increased water uptake, resulting in swelling and mechanical instability issues. Very recently, AEMs with hydroxide conductivity as high as 300 mS cm^–1^ were reported,^268^ thanks to their capability to operate at temperatures higher than 100 °C.^269,270^

Despite ongoing efforts to enhance alkaline electrolysis performance in pure water by utilizing conventional AEMs, optimal performance remains elusive due to the low ionic conductivity of these membranes in pure water. In general, the ionic conductivity of AEMs could be enhanced by increasing the ion exchange capacity (IEC); however, raising the IEC may lead to significant swelling or even dissolution of the membrane, ultimately compromising its mechanical stability. Cross-linking is commonly used to reduce dimensional changes, although this approach can adversely affect the solubility of the ionomer, which is essential for MEA preparation. Increasing the molecular weight of AEM polymers can also help to reduce swelling and improve mechanical stability. Consequently, developing an ideal AEM involves achieving a careful balance between moderate IEC and high molecular weight to ensure effective operation in pure water-fed conditions. While these issues for the AEM have been addressed to some extent, significant conductivity and stability issues remain for the AEI in the catalyst layer, which will be discussed later.

Nevertheless, AEMs still face water transport limitations unique to their operating requirements. For all membrane-containing WE systems, it is preferred to flow water to the anode only and operate the cathode nominally “dry” (with water only transported through the membrane to the cathode), as this reduces the amount of gas/liquid separation required in the BoP (Figure 9). However, in PEM-WEs, H^+^ is transported from the anode to the cathode, and therefore, electro-osmotic drag pulls additional water to the cathode, aiding electrode hydration. In an AEM with electrolyte or water feeding only to the anode, the flux of OH^–^ away from the cathode can lead to dehydration of the cathode interface and water transport limitations, especially at high current density and in the presence of ionic salts.^271^ It is worth noting that while water at the cathode in PEM-WE only matters for hydration, water at the cathode in AEM-WE serves as a reactant; thus, sufficient transport of water to the cathode is crucial for the AEM-WE system. However, the water transport behavior and swelling management during pure water operation are not fully understood.

Water transport or diffusion limitations significantly reduce the hydration levels in the cathode catalytic layer (λ < 6) and at the membrane/cathode interface. These low hydration levels greatly accelerate the chemical degradation of the ionomeric material in both the membrane and the cathode catalytic layer. This phenomenon is more pronounced at high current density, where the rate of water consumption exceeds the water flux from the anode to the cathode sides.^273^ Recently, Wang et al.^272^ conducted an investigation into the impact of water diffusion on the performance of AEM-WE during cathode dry operation, employing both “single AEM” and “double AEM” (two AEMs of same thickness stacked on each other) in MEAs as illustrated in Figure 13.A. Their findings revealed an enhancement in cell performance when utilizing the “single AEM”, indicating improved performance with thinner AEMs. Notably, one of their key observations pertained to differences in the relative humidity of the hydrogen at the cathode, which is related to water transport. Given that water diffuses from the anode to the cathode based on the gradient in hydration number, λ, and is subsequently consumed by the cathode reaction, they depicted λ profiles in the MEA for cells at the same current density (Figure 13.B). This figure illustrates that higher λ values are estimated in the AEM on the cathode side when employing the ’single’ AEM configuration, which is believed to be the cause for the enhancement in performance. Therefore, the introduction of electrolytes, e.g., dilute KOH, to the cathode side fails to accurately simulate the natural electrolyte-free environment of AEM-WEs, leading to misleading and inaccurate assessments of degradation rates, particularly under conditions of relatively low hydration expected during the dry cathode operation.

**Figure 13:**
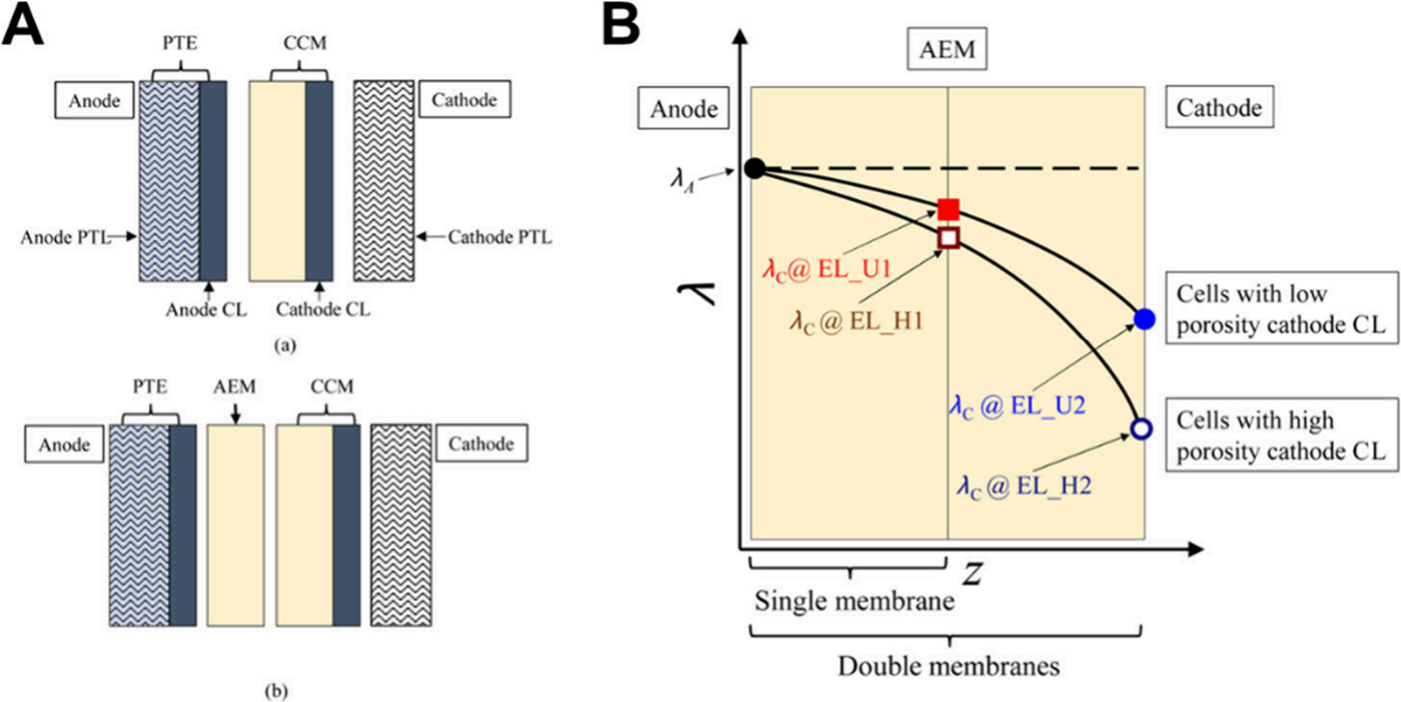
(A) Schematic representation of MEA structure, featuring both single and double AEM. (B) Schematic depicting the distribution of water content (λ) within the membrane in various electrolytic cells when current density ≥ 0.6 A cm^–2^. (A-B) were reprinted with permission from ref 272. Copyright 2022, Elsevier Ltd.

*Ex-situ* methods reported in the literature have been developed to assess the alkaline stability of AEMs immersed in aqueous alkali solutions for extended periods. Figure 14 provides an overview of various *ex-situ* methods found in the literature for determining the alkaline degradation of AEMs in alkaline solutions, focusing on evaluating changes in both performance and chemical structures. Generally, the hydroxide concentration in these alkali solutions is above 1 M, equivalent to hydration numbers higher than 6.^275^ Above this hydration level, sufficient quantities of water molecules are present to fill the first solvation sphere surrounding the OH^–^ ions and influence their nucleophilicity. In contrast, the performance of AEMs in pure water conditions remains a critical area of study, as conventional AEMs may exhibit low ionic conductivity under these circumstances, impacting their effectiveness in applications such as water electrolysis.

**Figure 14:**
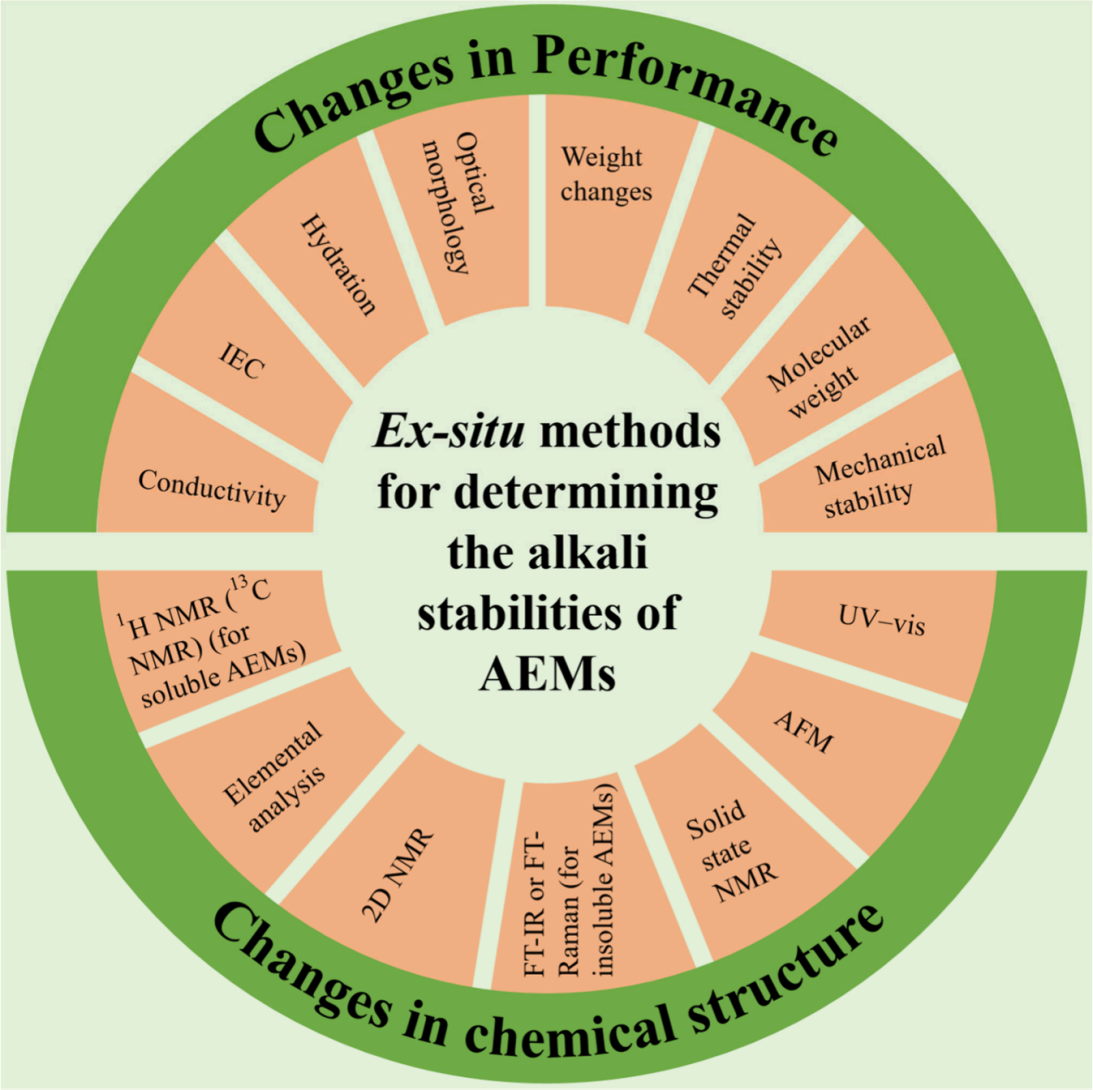
*Ex-situ* methods reported in the literature to assess the alkaline stability of AEMs. Data adapted with permission from ref 274. Copyright 2021, Springer Nature.

Figure 15.A represents a schematic diagram illustrating nucleophilic attack onto trimethylbenzyl ammonium (TMBA), both with and without the presence of water.^275–278^ This scheme highlights the role of water molecules, which reduce their nucleophilicity when firmly bound to the hydroxide, effectively acting as a ’shield’ against QA attack. To gain comprehensive insights into the chemical stability of AEMs in harsh alkaline environments, it is crucial to employ techniques that accurately replicate real-world conditions. Ex-situ methods, which involve characterizing AEM properties before and after their use in AEM-WE and AEM-FC, provide controlled environments where researchers can precisely manipulate hydration levels and other variables to mimic conditions experienced by AEMs in operation. This deeper understanding facilitated by ex-situ methods not only enhances the comprehension of AEM behavior but also aids in the selection of materials better suited for durable and efficient performance in practical applications.

**Figure 15:**
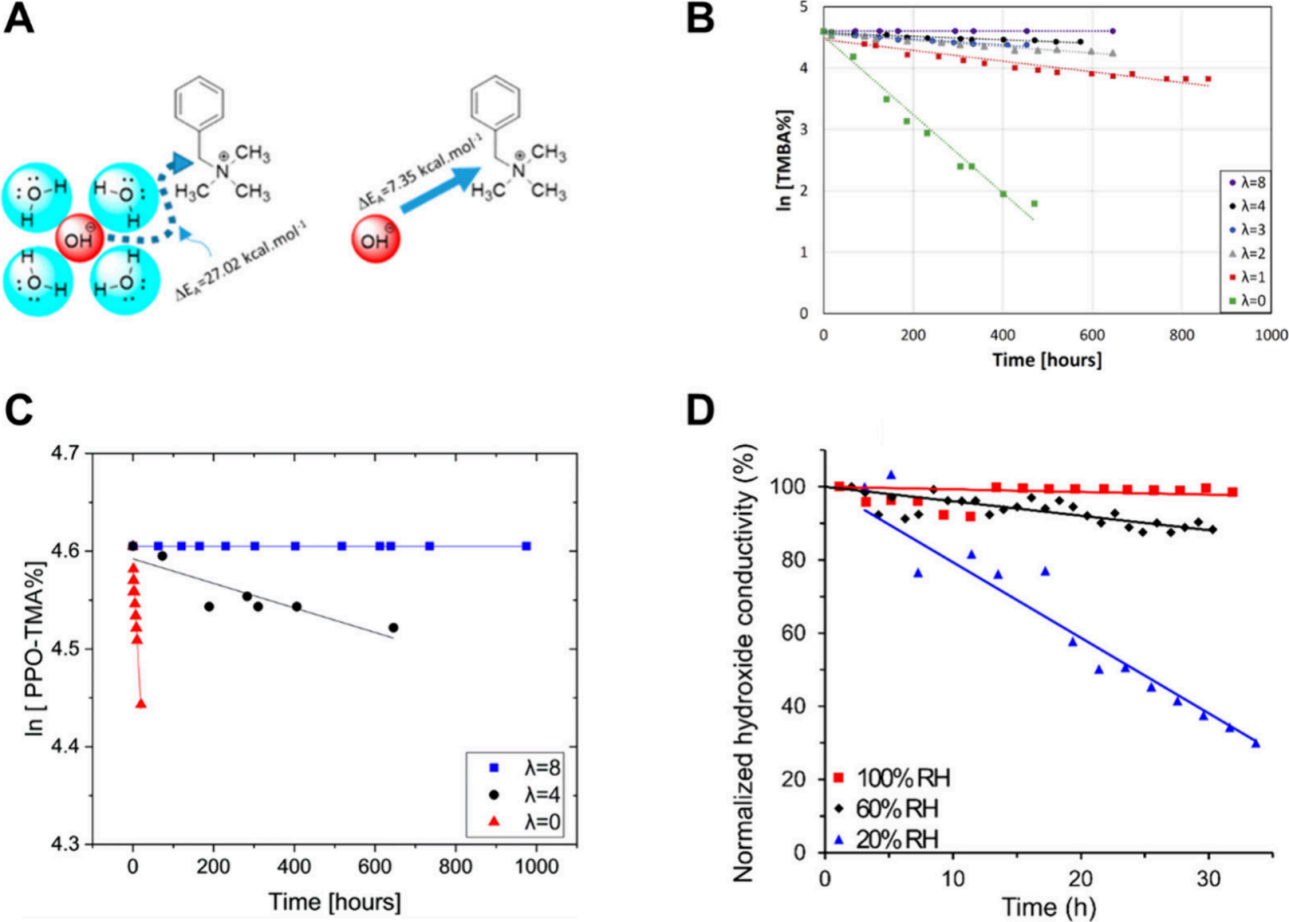
(A) Schematic diagram illustrating nucleophilic attack onto TMBA, with and without water. Figure reproduced from ref 276. Copyright 2017, American Chemical Society, Licensed under CC-BY 4.0. (B) Remaining TMBA fractions over time, varying with the number of water molecules per OH^–^ (λ = 0–8), in 0.6 M OH– DMSO-d6 solutions at room temperature. Figure reproduced with permission from ref 275. Copyright 2017, Elsevier B.V. (C) Remaining PPO–TMA fractions as a function of time with λ = 0, 4 and 8, in 0.06 M OH^–^ DMSO-*d*_6_ solutions at room temperature. Figure reproduced from ref 277. Copyright 2018, Royal Society of Chemistry. Licensed under CC-BY 3.0, (D) Normalized true OH^–^ conductivity of the BTMA-LDPE AEM as a function of test time (80 °C, 100 μA, and a nitrogen flow of 500 sccm/min) at different RH levels. Figure reproduced from ref 278. Copyright 2020, American Chemical Society. Licensed under CC-BY 4.0.

Dekel and co-workers^278–281^ developed a practical and reproducible *ex-situ* technique for simulating the true environment in MEAs. This method allows for measuring the harsh alkaline environment at low hydration levels (specifically at the cathode side), mimicking the true nature of AEM-WE and AEM-FC devices. The alkaline stability of AEMs has been evaluated by storing membranes in alkaline solutions at elevated temperatures.^282^ However, the alkaline stability of AEMs was measured without using hydroxide-based electrolytes, but with various hydration levels. A comprehensive examination was conducted to identify the influence of water on the chemical stability of the QA functional group. As illustrated in Figure 15.B, the degradation rate of TMBA exhibits a noticeable increase as the water content decreases.^275^ Furthermore, the protocol was extended to assess the stability of QA functional groups within the AEM backbone. Figure 15.C illustrates a reduction in the chemical stability of poly­(phenylene oxide) quaternized with trimethylamine (PPO-TMA) as the water content decreases.^277^ Additionally, by employing this technique, the AEM degradation under varying and more rigorous environmental conditions was investigated, replicating the operational conditions.

Figure 15.D demonstrated a more rapid decline in the normalized true OH^–^ conductivity of BTMA-functionalized LDPE-based radiation-grafted AEM as relative humidity decreased, indicating an increased chemical degradation of the AEM over time.^278^ Adopting such a method should provide further insights into the membrane behavior inside the AEM-WE. Accordingly, it offers important indications for developing highly conductive and stable AEM for liquid-electrolyte-free AEM-WE.

#### 5.3.2 Role of Ionomers

AEIs are a crucial component in CLs used in AEM-WEs. These polymers contain positively charged functional groups, act as a binder for electrocatalyst particles and create “a passageway” for ions to travel by improving the contact between the mentioned components (EC, the PTL, and AEM) in the MEA. In practical terms, similar to the case of AEM-fuel cells, the use of ionomers with high ionic conductivity can broaden the three-phase region, leading to an increased active area within the EC layer during the electrolysis of pure water.^283^ Additionally, ionomers in the CL facilitate the exchange of water and ionic products at the EC surface through their charged functional groups.^284,285^ Finally, maintaining good adhesion of the ionomer and EC to the current collector in the electrode is particularly crucial for AEM-WEs that operate with liquid water and gas evolution at the electrodes. These gases can accumulate and form bubbles between the EC layer and the membrane, which can compromise the adhesion of the layers and ultimately lead to the delamination of the MEA.^286^ Removing evolved gas bubbles from the EC-AEI interface may be a critical factor for high current density performance for DI water-fed AEM-WEs.^252^

Under fully hydrated conditions, ionomers with a high IEC and water uptake have a more significant dimensional change,^287^ which causes ionomer detachment from the EC.^181^ Due to the small EC-electrolyte interfacial area, this phenomenon is more common in AEM-WE operated with pure water. As a result, chemical or electrochemical degradation of the ionomer may significantly decrease the reaction rate. It has been recently revealed that higher IEC ionomers with light cross-linking are favored in the cathode electrode as this fine-tunes the water uptake and swelling of the ionomer.^288^ Particularly, a chemically tailored OH^–^ conducting polymer, poly­(2,6-dimethyl-1,4-phenylene oxide) (qPPO), was synthesized via amination and subsequent quaternization and it was blended with poly­(vinyl alcohol) (PVA) to provide an environment analogous to basic water solutions.^262^

Huang et al.^288^ showed the application of cross-linked ionomers at the anode of an AEM-WE operated with 3% K_2_CO_3_ solution. The cross-linking in ionomers with high IEC allowed them to limit hydrophilicity and water uptake, enabling them to have high ionic conductivity without the penalty of excess swelling. In this work, electrodes prepared using ionomers with IEC as low as zero outperformed ionomers with high IEC due to the excessive swelling of the latter ones, causing an increase in ionic and electronic resistance, and EC detachment. Adding a hydrophobic agent (PTFE) to control the water content in the AEM-WE anode CL was found to be less effective than the cross-linking within the ionomer to limit swelling caused by the ionomer water uptake. In particular, cross-linked ionomers with high IEC had comparable water uptake to non-cross-linked ionomers with low IEC but showed better performance (∼150 mV lower cell voltage) when used at the AEM-WE anode. A trade-off between water uptake and IEC of the ionomer is crucial to achieving optimal AEM-WE performance, and the proposed ionomer cross-linking approach enabled us to take advantage of the benefits of a high IEC by mitigating the swelling.

Light cross-linking was also shown to independently reduce ionomer water uptake in the anode electrode while maintaining high ionic conductivity.^288^ Koch et al.^289^ showed that under dry cathode operation, the cell with high IEC ionomer (IEC (AP2-HNN8–00-X with IEC of 2.3–2.6 meq g^–1^) in the cathode performs better than the mid ionomer IEC (AP2-HNN6–00-X, IEC = 1.8–2.2 meq g^–1^) and has lower high-frequency resistance (HFR). This is explained by the better water retention in the MEA compared to the lower IEC ionomer over the whole current density range. It was found that dry cathode operation can cause a considerably higher degradation rate, but this effect is partially reduced when an ionomer with a higher IEC is used in the cathode EC layer. This suggests that utilizing ionomers with high IEC in the EC layer could provide an alternative to flowing liquid water to the cathode and as well as a way to reduce degradation rates associated with dry cathode operation, hence contributing to improved performance and durability. Therefore, developing high IEC ionomers with moderate water uptake that ensures high ionic conductivity and stability is critical for achieving high performance and durability in pure water-fed AEM-WE.

Recent work from Mayerhöfer et al.^290^ showed the impact of the ionomer content on the anode prepared using a PGM-free EC (Cu–Co oxide) deposited on a Ni-felt PTL. Two systems containing 10 and 30 wt % of Aemion ionomer were compared in pure water and 0.1 M KOH solution. With DI water, the electrode with higher ionomer content (30% vs 10%) performed better, while an opposite trend was observed in the presence of a supporting electrolyte (0.1 M KOH). The AEM and the AEI undergo a different swelling degree in different electrolytes having different pH (7 to 12.7), varying from above 100% in pure water in pristine condition to less than 50% in 0.1 M KOH electrolyte.

In addition, the sole presence of AEI within the electrode of the AEM-WE operated in DI water, neutral pH, may not be sufficient to provide a high enough pH, especially during operation, when OH^–^ ions are consumed at the anode. Therefore, the presence of supporting electrolytes, even in a low concentration, can increase the performance of the cell. However, an excessive amount of AEI in the anode CL can reduce the accessibility to the EC active sites, lowering the AEM-WE performance. AEIs in the AEM-WE anode CL require a different approach when operating with DI water or with a supporting electrolyte.

The work of Motz et al.^291^ showed how the chemical structure and the physical properties of the ionomer influence the performance of an AEM-WE. Various AEMs and AEIs with different cationic functional groups were selected and tested in DI water, 1 wt % K_2_CO_3_ and 1 wt % KOH. These AEMs were: (i) alkyl trimethylammonium functionalized poly­(styrene-ethylene-styrene) block copolymer (SES-TMA), (ii) alkyl trimethylammonium functionalized Diels–Alder poly­(phenylene) (HTMA-DAPP) and (iii) polytetrafluoroethylene (PTFE) reinforced alkyl ammonium tethered poly­(meta-terphenylene) (m-TPN1) (Durion, Xergy). The AEIs tested had different cationic groups, such as Alkyl ammonium (HTMA-DAPP and FLN55) and Benzimidazolium (HMT-PMBI). The results showed that the impact of the ionomer on the performance is larger when the AEM-WE is operated with DI water. In fact, AEI FLN55 possesses higher IEC than HTMA-DAPP AEI (2.5 meq. g^–1^ vs 1.5 meq. g^–1^), higher water uptake and higher hydroxide conductivity. AEM-WE with AEI FLN55 showed higher performance and higher operational stability in pure water. Another conclusion from this study is that the different AEIs tested (FLN55, HTMA-DAPP and HMT-PMBI) in DI water play a more significant role than the respective AEMs on the AEM-WE performance, pointing out the crucial importance of the EC-ionomer interaction within the electrode, even more than the AEM itself, as shown in Figure 16.A. The ionomer plays a more important role, especially in DI water operation, which is demonstrated by the larger differences in performance observed in DI water compared to operation with an alkaline supporting electrolyte solution.

**Figure 16:**
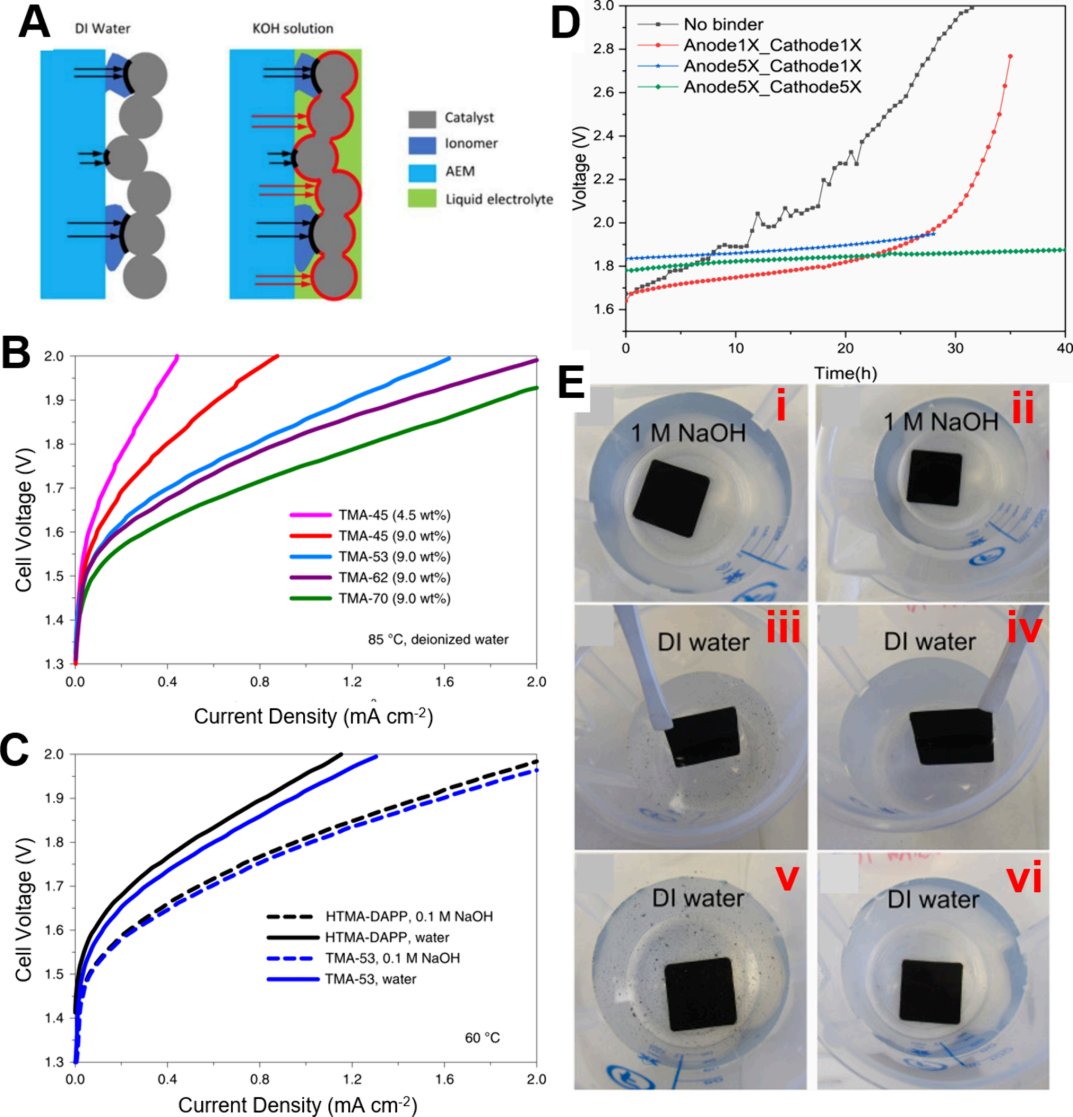
(A) Comparison between the OH^–^ ions pathway in the anode of an AEM-WE operated with DI water (left) and with a supporting electrolyte solution (right). EC-ionomer interface is marked in black and the EC-liquid electrolyte interface is marked in red. Reproduced from ref 252. Copyright 2021, IOP Publishing Limited under CC BY-NC-ND 4.0 license. (B) AEM-WE performance of MEAs where AEIs with different IECs were used. (C) MEA performance comparison between HTMA-DAPP-bonded and TMA-53-bonded MEAs at 60 °C in water and 0.1 M NaOH. (B–C) were reproduced with permission from ref 181. Copyright 2020, Springer Nature Limited. (D) Cell voltage vs time for MEAs with various epoxy binder contents at 0.5 A cm^–2^. Reproduced with permission from ref 293. Copyright 2022, Elsevier B.V. (E) CL delamination observed without Nafion binder when exposed to DI water vs no delamination observed when Nafion binder is used; observing binder-free (i) and binder-containing (ii) electrodes in 1 M NaOH, when binder-free (iii) and binder-containing (iv) electrodes immersed in DI water, and eventually the binder-free (v) and binder-containing (vi) electrodes after being left in the DI water for several hours. Reproduced with permission from ref 294. Copyright 2022, Elsevier B.V.

In a recent work from Lindquist et al.,^56^ three different commercial materials (PiperION from Versogen, Sustainion from Dioxide Materials, and Aemion from Ionomr) were tested. Here, AEIs were used in combination with the respective AEMs, and the AEI amount on the electrode was kept to 10 wt % for all three materials. The cell voltage difference for the three materials is significant in pure water operation, ranging over more than 200 mV at the same current density of 1 A cm^–2^. The three materials also showed considerably different durability behavior when subjected to a durability test at a constant current density of 500 mA cm^–2^ in DI water. The PiperION MEA was more durable overall and started from a lower voltage due to better performance at the beginning of the test (∼ 1.85 V vs ∼ 2.00 V of Sustainion and Aemion). However, all three ionomers showed a similar high degradation rate in the first 20 h of the test, in the range between 11 and 15 mV h^–1^.^56^ These results pointed out how challenging the durability of AEM systems operated in DI water is, mostly due to ionomer degradation. In fact, a high loading (2.3 – 2.8 mg cm^–2^) of commercial PGM EC (IrO_2_) was used for the tests, which is unlikely to be the primary reason for degradation in OER catalytic activity.

Another recent work from Li et al.^181^ highlighted the huge impact on the AEM-WE performance due to the ionomer IEC and the ionomer content. With the same ionomer chemistry (trimethylammonium functionalized polystyrenes (TMA-x)) but different IEC (in the range 2.2 – 3.3 meq. g^–1^) and by tuning the ionomer content in the electrodes, a performance improvement of ∼ 400 mV at 0.4 A cm^–2^ was achieved in pure water operation (Figure 16.B). The same paper also showed how ionomer IEC and loading impact the performance more in pure water than with supporting electrolytes (0.1 M NaOH). (Figure 16.C). Confirming earlier observations, an increase of an ionomer content (up to 20 wt %) improves the performance of DI water operation. However, an excessive amount of the ionomer (27.3 wt %) resulted in a performance decrease, which could be related to a poor EC dispersion and, in a limited ionomer-EC interface, essential for OH^–^ transport in the absence of a supporting alkaline electrolyte.^181^

The work of López-Fernández et al.^292^ reported higher performance in a 1.0 M KOH-fed AEM-WE of an ionomer-free anode electrode in comparison with the same electrode prepared with ionomer added on the electrode surface and ionomer mixed with the EC. In this study, the electrodes were fabricated using magnetron sputtering, a physical vapor deposition process avoid the EC dispersion in a solvent before deposition onto the electrode. The results of this work confirmed that no AEI is needed (other than for binding purposes) when operating with a highly concentrated supporting electrolyte.

Chen et al.^293^ showed that the addition of a nonionic conducting binder within the anode CL helps to increase the durability of AEM-WE (0.1 M NaOH electrolyte), preventing EC detachment without compromising the initial performance. Butyl norbornene (BuNB), bromobutyl norbornene (BBNB), and *tert*-butyl ester norbornene (terpolymer 1), norbornene-2-propionic acid ethyl ester (terpolymer 2), or epoxyhexyl norbornene (terpolymer 3) were the initial monomers used. However, the insertion of covalent bonding sites for covalent chemicals, in this case bonding of bis­(phenyl)-A-diglycidyl ether within the ionomer itself, creates a “self-adhesive” ionomer, enabling it to obtain even better performance and durability (Figure 16.D). Achieving a good and durable adhesion of the anode EC onto the PTL is more challenging than for the cathode EC, because typically the anode compartment is fed with water or electrolyte solution, and therefore within the anode CL, there is the creation of additional tension forces due to the formation of O_2_ gas bubbles. On the other hand, the cathode is operated in dry conditions, allowing the H_2_ gas to evolve more easily.

Osmieri et al.^294^ addressed the issue of detachment of a PGM-free (LaSrCo oxide) CL from the PTL when exposed to pure water. AEM and AEI underwent the initial ion-exchange step in 1 M NaOH solution to exchange the HCO_3_^–^ ions present. This procedure was done to convert the AEI within the CL to hydroxide form before the test. No CL detachment was observed in 1 M NaOH, but as soon as the electrode was immersed in DI water, delamination of the CL layer occurred (Figure 16.E). This behavior was attributed to possible changes in AEI swelling properties at different pH and/or ionic strength. The authors mitigated this detachment issue by adding Nafion ionomer to the ink dispersion. Thanks to the superior binding properties of Nafion, the CL delamination when exposed to DI water did not occur. This resulted in better durability of the AEM-WE in DI water operation. The authors showed that a fine-tuning of the AEI-to-EC ratio and the AEI-to-Nafion ratio may improve performance. In fact, three conditions were examined: AEI/EC ratio of 0.2 and 0.4 and Binder/AEI ratio of 0.32 and 0.16. The conditions with low AEI/EC (0.2) and low Binder/AEI ratio (0.16) obtained the best performance in AEM-WE, a single polarization curve, but it did not retain its performance in short-term tests, showing a higher degradation rate, probably caused by the ionomer degradation. Attention should also be paid to the AEI-to-Nafion (AEI/Binder) ratio as Nafion operates as a binder to keep the EC in the catalytic layer, reduce the AEI and AEM swelling and avoid delamination. However, a reasonable amount of AEI is needed to favor hydroxide exchange. This aspect is particularly important for DI water operation, where OH^–^ ion conductivity is assured by the AEI within the CL. When operating with a supporting electrolyte solution, the impact on the performance of this CL composition optimization was found to be less impactful due to the excess of OH^–^ ions.^294^

#### 5.3.3 Role of Electrode and Active Layer Fabrication and Structure

Several variables in different electrode fabrication strategies may influence the MEA performance for electrochemical energy conversion devices like fuel cells and WEs.^295,296^ Many of these variables are directly related to the structure of the CL and interfaces of CL/membrane and CL/PTL.^297–300^

As it has already been discussed, ionomer plays an essential role as a binder and ion conductor in the EC layer of the device. The EC layer is deposited on the membrane (catalyst-coated membrane, CCM) or on the PTL (catalyst-coated substrate, CCS) using ink. The ink is comprised of the EC, solvent, and dispersed ionomer that acts as an EC binder and enables ion transport to the active EC surface.^301^ The challenging part of the ink preparation lies in a fine balance of the formulation – increased content of the ionomer-binder often leads to the EC-particle agglomeration and sedimentation of the solids in the ink. The quality of the ink and EC layer deposited can impact device performance to the same degree as the properties of the individual components.^56^ When the ink is deposited, the solvent evaporates and creates a porous layer of ionomer, EC, and void space for liquid/gas transport to/from the EC. An EC ink is a colloidal liquid with intrinsic interactions that control the electrode formation. Modeling efforts were made to describe the behavior of an EC dispersion; however, more studies must be performed to account for all the effects, such as rheology, dielectric constant and its influence on the aggregation within the ink.^302,303^ Additionally, the processing technique (Doctor blade coating, spraying, paint-brushing) has to be considered when discussing the ink’s influence on the final performance of the device.^304^ The lack of a thorough understanding of EC ink affects the reproducibility of AEM electrolysis studies. The interactions between materials in this region directly impact device performance and durability^56,285,305^ as the impedance of electric, ionic, and reactant/product transport to/from the reaction zone reduces performance.^306^

CCM and CCS fabrication methods are shown in Figure 17.A.^206^ In the CCM method, the EC ink is deposited on the membrane, enabling a more intimate contact between the membrane and CL. It has been reported in the broad literature on PEM devices that the CCM method improves interfacial electric contacts at the EC-membrane interface and maximizes the EC utilization,^307–309^ which could work similarly in the AEM system. In the CCS method, the EC ink is deposited onto the porous substrate (PTL), and the advantage of this method is that it is typically easier to deposit the ink on the PTL substrate than on the membrane.^310^ The reason for this is that AEMs have poor mechanical and/or physical properties (i.e., lower thermal stability, high water uptake and swelling), which make their handling in the CCM process more complicated compared to PFSA PEMs. In particular, polyarene-based AEIs typically used to fabricate AEMs have glass transition temperatures higher than 200 °C, which complicates the adhesion to a heated vacuum plate typically used for transferring spray-coated CL on the plate to the membrane.^299,311^ While employing the CCM method, the membrane wrinkles during spray coating and drying, or even transferring of the CL onto the membrane via a thermal decal process.^311,312^ To overcome these issues, Koch et al.^312^ recently proposed an alternative process for CCM fabrication using a bar coating deposition method on an AEM with a backing layer. The first deposited CL is covered with an adhesive backing foil, which prevents the membrane from wrinkling during the coating of the second CL. This method was demonstrated to create performing MEAs, and it is promising for the scale-up transition to roll-to-roll manufacturing highly employed in the industry (Figure 17.B). Particularly, the AEM-WE performed 1 A cm^–2^ at 1.8 V with ≥ 1.9 mg_Ir_ cm^–2^ anode loading that was comparable to spray-coated cells. A fully self-casted CCM with a thickness of 35 μm was able to achieve a performance of 1.4 A cm^–2^ at 1.8 V (0.1 M KOH) and 2.0 A cm^–2^ with 1 M KOH supporting electrolyte.

**Figure 17:**
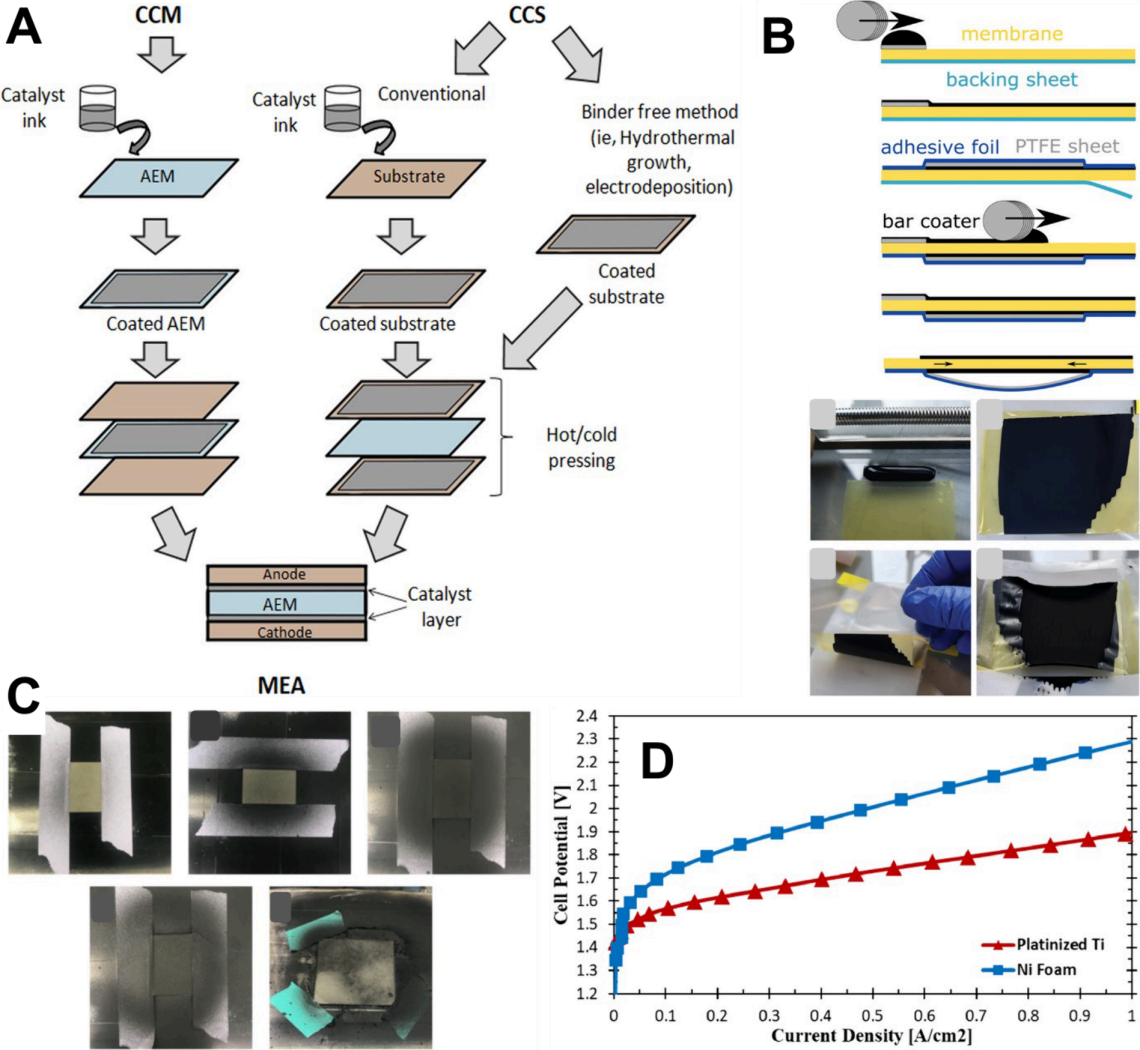
(A) Schematic representation of MEA fabrication via CCM and CCS methods. Reproduced with permission from ref 206. Copyright 2021, John Wiley & Sons Limited. (B) proposed process for direct coating of an AEM with the CCM method using a bar coater. Reproduced with permission from ref 312. Copyright 2022, Wiley-VCH GmbH. Licensed under CC BY 4.0. (C) progression of spray coating on stainless-steel PTLs showing that slow spraying ensures ink drying between layers, and that too quick coating generates nonuniform coating with ink seeping. Reproduced with permission from ref 56. Copyright 2021, The Royal Society of Chemistry. (D) polarization curves showing the effect of the anode PTL type (Ni foam vs platinized Ti) on the performance of an AEM-WE fed with 1 wt % K_2_CO_3_. Reproduced with permission from ref 295. Copyright 2022, Elsevier B.V.

The CCS method has been more commonly used in AEM-WE so far. Lindquist et al.^56^ highlighted that the quality of the EC ink deposition has a significant effect on the overall MEA performance in the CCS process. They pointed out the importance of the anode EC ink flow rate and drying time when spraying an IrO_2_ EC ink onto a stainless-steel mesh PTL material using a hand-held spray gun. If the coating procedure occurs too quickly, without allowing enough time for the ink to dry between subsequent layers, the PTL becomes too wet, pulling the ink into the bulk and leaving areas with uncoated surfaces. Even after continued spraying, these areas do not coat evenly (Figure 17.C).

More recently, Osmieri et al.^294^ used an automated spray-coating system to achieve a uniform coating of a PGM-free EC (LaSrCo oxide) onto a sintered titanium PTL, as the automated system enables precise and simultaneous control of the flow rate and nozzle path. On the other hand, when the ink deposition rate is not controlled (e.g., when the ink is deposited via hand painting) the high porosity and hydrophilicity of the PTL material cause the ink to massively seep within the PTL, resulting in a largely nonhomogeneous coating, and reducing the contact between CL and membrane during the cell assembly. Tricker et al.^175^ compared a hand airbrush and an ultrasonic spray coater using Co_3_O_4_ OER EC. The results of an AEM-WE fed with 1 M KOH showed better performance for the airbrush method, which enabled obtaining a more porous CL with fewer cracks, favoring EC accessibility. The ultrasonic spray coating method caused many EC particles to penetrate within the porous structure of the PTL, and higher HFR.^175^

As part of CCS or CCM, hot-pressing can be used to improve the CL-membrane interface. Here again, poor thermal stability and swelling properties of AEMs or AEIs make this process challenging. The impact of MEA hot pressing and cell torque on the AEM-WE performance was investigated by Lim et al.^313^ They showed that with a CCS fabrication method to deposit commercial IrO_2_ and Pt/C ECs onto titanium felt and carbon paper PTLs, respectively, the hot pressing was detrimental, and an optimum torque value should be used to avoid solution leaking and excessive structural deformation of the CL.

There are some reports comparing CCM and CCS methods. The work of Park et al.^310^ showed better AEM-WE performance in 1.0 M KOH using the CCM method compared to the CCS method. The CCS method with hot pressing showed similar HFR to the CCM method, but higher mass transport resistance. As a comparison, the CCS method without hot pressing showed much higher HFR. An opposite trend was reported in the work of Gupta et al.^314^ where better performance with the CCS method was obtained in an AEM-WE fed with 0.1 M KOH. These contrasting results in the KOH-fed system suggest that understanding and careful optimization of the EC layer deposition procedure are essential to improving the AEM-WE system. There is no study reported for direct comparison between CCM and CCS in pure water-fed systems, but key discoveries from the KOH system could help understand the pure water system. However, as hydroxide ion transport is limited to where ionomers are incorporated into the EC layer and EC utilization is expected to be different without supporting electrolytes, careful adoption from the literature of the KOH system to the pure water system will be required.

Other than the fabrication of electrodes from formulated ink dispersions, recently, approaches to obtain self-supported anode electrodes have been reported. Highly active Ni–Fe oxyhydroxides ECs were directly grown onto Ni foam using a galvanic-dissolved oxygen corrosion method from Xiao and co-workers,^315^ and a hydrothermal reaction from Wan and co-workers^316^ respectively. This approach potentially enables us to avoid some of the CL fabrication steps previously described, like ink formulation and deposition, and simplify the electrode fabrication steps. For operation in DI water-fed AEM-WE, the presence of ionomer is essential, and these two works propose ionomer incorporation by dip-coating^315^ and spray-coating.^316^ Kong et al. showed that Ni_3_Fe_1_ EC layers were deposited on membranes in MEA conditions where metal precursor and reducing agent, NaBH_4_, were flown through the cell. The in situ catalyst-coated membranes showed a cell voltage of 1.79 V at 1 A cm^–2^ with 1 M KOH feed and 1.91 V at 250 mA cm^–2^ with pure water feed.^325^ The anode EC loading plays an important role in the performance of AEM-WE cells. The general trend of increasing performance with higher EC loading when operating with a supporting electrolyte was demonstrated by Mayerhöfer et al.^290^ and Hassan et al.^295^ The relationship between EC loading and performance is currently missing for AEM-WE; however, an increasing trend can be expected up to a certain point, when a further increase in the loading will lead to overly high mass transport and charge transport resistance and will lower the cell performance.

The recent work of Tricker et al.^175^ devoted attention to aspects related to the cathode of the AEM-WEs, which has not been largely explored so far. The authors investigated three aspects of the cathode: EC loading (using a Pt/C HER EC), the addition of a microporous layer (MPL), and GDL hydrophobicity. The AEM-WE was operated with 1 M KOH. They found that significant performance improvement was obtained when increasing the Pt/C EC loading from 0.15 to 0.3 mg_Pt_ cm^–2^, but no significant difference was measured when the loading was further increased up to 0.45 mg_Pt_ cm^–2^.

The microstructure and density of the EC layer also affect the performance in AEM-WE cells. For example, in the work of Park et al.,^317^ despite the same EC loading, improved performance was obtained from creating a highly macroporous structure of the anode CL directly grown on the PTL, in comparison to a conventional ink-based CL prepared using the CCM method. The improvement was due to an increase in the accessible EC surface area, and it was obtained for two different ECs (IrO_2_ and NiFe alloy) in an AEM-WE operated with 1.0 M KOH. Wang et al.^318^ highlighted the importance of having a highly dense anode CL in close contact with the PTL to enhance performance. They used a CuCoO_*x*_ OER EC deposited onto Ni foam PTLs with different thicknesses. With a 10% K_2_CO_3_ solution as a supporting electrolyte, decreasing the thickness of the PTL material and the EC loading at the same time enabled the achievement of similar AEM-WE performance due to the similar compactness of the CL.

It is of primary importance to emphasize the essential role played by the EC-ionomer interface in dictating the performance of the AEM-WE operated with pure water. This interface is created within the EC layer during the electrode fabrication process, which was analyzed in detail in this section. It is imperative to stress once again that in the case of pure water operation, the only path for OH^–^ ions conduction is through the AEI within the CL. Therefore, in this case (as evidenced in Figure 16.A), the only electrocatalyst-electrolyte interface is represented by the interface between the EC and the AEI. In addition, a good interface between the CL and the AEM is needed to enable continuity of the ionomer phase (and thus the OH^–^ ions) between the CL and the AEM. This is particularly important for the anode, where the OH^–^ ions represent the reactant species for the OER.

It becomes thus clear that for increasing the performance in the case of pure water operation, the thickness of the CL should be minimized to reduce the length and the tortuosity of the ionomer path for OH^–^ ions conduction, consequently reducing the OH^–^ transport resistance within the CL. However, for CLs fabricated using an EC in powder form, the CL thickness is directly proportional to the EC loading, and thus to the amount of (theoretically) available active sites. Excessively reducing the EC loading, aiming to decrease the thickness of the CL, will inevitably result in AEM-WE performance decrease, as recently demonstrated in the work of Kreider et al.^319^ In this work, it was found that higher anode EC loadings are beneficial for highly active OER ECs with high electronic conductivity and uniform CLs, while for less performing ECs with lower conductivity and/or less uniform CLs, the EC loading has a minimal impact on performance.

However, typical well-established electrode materials used for conventional A-WE operating in highly concentrated KOH solutions may represent an “easy” and straightforward path toward a transition to AEM-WE systems. These electrodes do not require the deposition of a CL using an ink where the EC is in powder form. Conversely, they can be represented as “self-supported” catalytic systems, or “catalyzed PTLs”, where the whole surface of the diffusion media (typically constituted by a foam, grid, mesh, or other porous structures) exposed to the electrolyte is catalytically active for the OER or the HER. The advantage of such a configuration is that it simplifies the electrode manufacturing process, without involving the CCM and CCS processes described earlier in this section. Several examples have been reported in the literature, also very recently.

In A-WE operating with supporting electrolyte at high concentration, the problem of the OH^–^ ions conductivity does not exist. However, when the electrolyte concentration is lower, up to the extreme case of pure water operation, this configuration presents important difficulties to implement, since the catalytic material covers the entire surface of the porous electrode, and in these conditions, it is challenging to create a uniform AEI layer with low tortuosity from the AEM to the EC active sites.

Recently, an AEM-WE system operating with a supporting electrolyte (1 M KOH) in which both the anode and cathode were fabricated by direct hydrothermal deposition of PGM-free ECs (NiFe-LDH and Mo, respectively) on Raney nickel substrate has been reported. The electrodes were completely AEI-free since no ionomer is needed to conduct OH^–^ ions with highly concentrated electrolyte.^320^ Another recent work by Oh et al.^321^ reported an improved performance and durability of an AEM-WE operating in 1 M KOH with both self-supported anode and cathode electrodes, different compared to the conventional CL electrodes.

Self-supported electrode approaches for both the anode and the cathode were also reported to be successful in achieving good performance in AEM-WE operated with pure water. The AEI, essential for operation in pure water, was incorporated into the electrode via spray coating. Two AEM-WEs prepared with self-supported and powdery anode ECs showed comparable performance when operated in pure water, although the electrode prepared with the powdery EC performed slightly better at high current densities, as expected in view of the previous considerations.^322^

A recent study showed that when operating the AEM-WE with supporting electrolyte feed, the type of anode PTL plays a crucial role. The authors investigated the use of different PTL materials with and without the addition of a CL. Depending on the PTL material used, the AEM-WE performance varied considerably. With a stainless steel PTL (containing Fe) without an EC, the performance was even better than the case where an Ir EC was used, confirming the much more straightforward anode configuration for operation in the presence of concentrated supporting electrolyte.^323^ Another recent work showed the superior performance of an AEM-WE with an anode consisting only of a stainless steel PTL (without any additional EC) compared to traditional CL-based electrodes.^324^

An interesting in situ formation of a CL without the use of AEI directly on the Ni fiber anode PTL with the MEA already assembled in the cell hardware was also reported, showing promising performance in 1 M KOH and also ultrapure water operation.^325^

Generally, it has been repeatedly observed that AEM-WE operated with pure water feed shows poor durability due to the rapid oxidation of the AEI in contact with the OER EC within the anode CL. An interesting attempt to mitigate this issue has been recently reported in the work of Kwak et al. The authors proposed the insertion of a thin layer (2–3 nm) of Hf oxide, which is electronically insulating but OH^–^ ions conductive, on the surface of the anode EC. This resulted in a considerable improvement in durability with only a negligible performance penalty.^326^

### 5.4 Bipolar Plates and Porous Transport Layers

Besides the MEA, which is the core of a membrane-based WE, other components such as the porous transport layers (PTLs) and the bipolar plates (BPs) also play an important role in determining the overall performance. The basic architecture of AEM-WEs was presented in the Introduction section and showed that to achieve high productivity, many MEAs must be interconnected among them at a stack level. There are two ways in which the interconnection between the individual cells can be realized. The first is the simplest and is the so-called monopolar design. In such a configuration, each cell is connected in parallel to form a stack. Despite its simplicity and ease of fabrication, the monopolar configuration has the major disadvantage of having high ohmic losses. To avoid the limitations associated with ohmic losses, the configuration based on BPs was introduced. With BPs, the cells are connected in series, with the voltage being equal to the sum of the voltage at each single cell and the current that is equal to the current that passes from each single cell.^327^ A schematic of the two configurations is reported in Figure 18.

**Figure 18:**
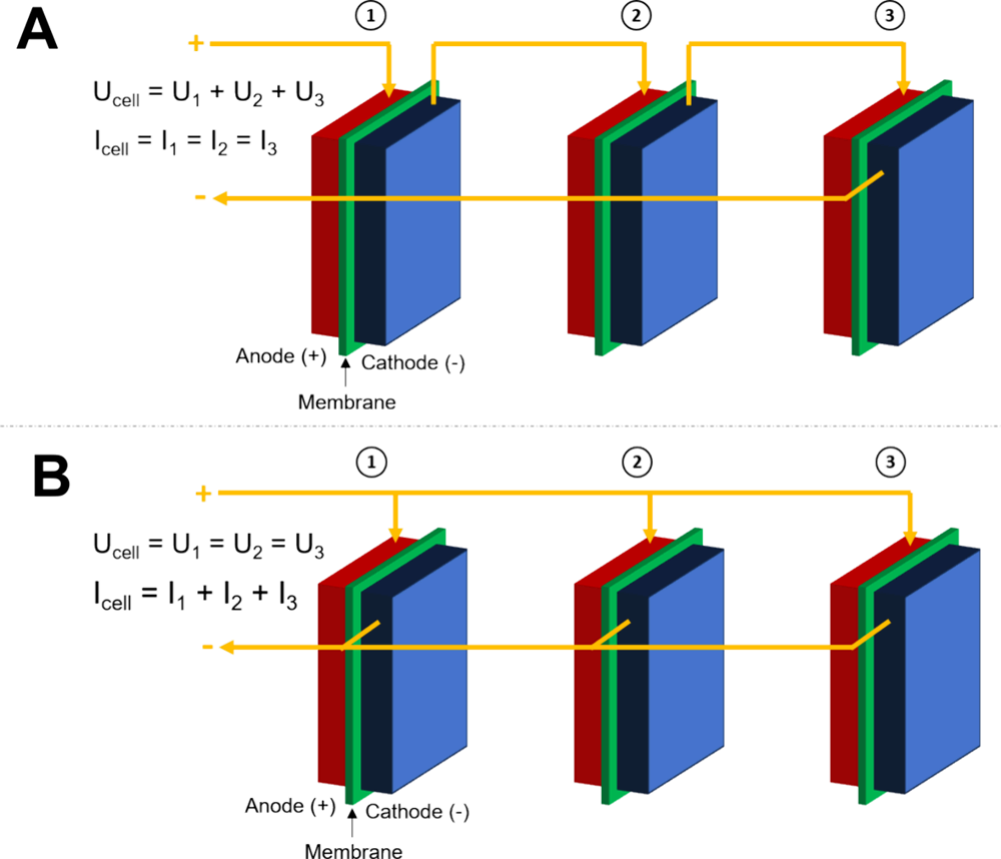
Water electrolyzer stack assembled in an (A) monopolar configuration and (B) bipolar configuration.

The current PEM-WE and AEM-WE technologies employ a bipolar configuration of the cell. In PEM-WE and AEM-WE, besides connecting the cells by having isolated cathodes and anode sides, the function of the BP is to allow the transport of the electrolyte and the product gases to and from the electrodes, respectively, via carefully engineered flow fields at the surface of the BPs. The flow field can have different shapes, such as single serpentine, double serpentine, triple serpentine, parallel^328^ and pin type.^329^

BPs must also allow proper thermal management of the cell via thermal conduction to guarantee a heat removal rate sufficient to avoid overheating.^330,331^ We remark that, so far, the BPs are the most expensive components of AEM-WEs, even more than the costly PGMs base ECs of PEM-WEs.^16^ The stack usually accounts for roughly 45% of the whole electrolysis system cost and in PEM-WE, the BPs account for more than 50% of the cost of the stack.^16^ In PEM-WE, the highly acidic environment together with the high potentials experienced at the anode require the use of bulk titanium, which is expensive and classified as CRM the EU.^332^ Moreover, to prevent the material corrosion, proper coatings can be envisaged. In this regard, alkaline technologies are less demanding, as the number of materials, also relatively inexpensive, that can resist corrosion even at more than 2 V is much larger.^282^ Typically, stainless steel and graphite were employed, cutting the cost compared to the use of titanium-based materials or complex-coated architectures.^310^ However, corrosion can still be an issue. Indeed, stainless steels with high Ni and Cr content may undergo severe passivation at the anode. This might not compromise the integrity of the device but may add ohmic drops due to the high resistance of the passivation layer, with an overall increase in the area-specific resistance of the device, as already shown in solid polymer fuel cells also in ref 333. On the other hand, the use of graphite is not suitable. That is mostly because of the high anode potential and the presence of OH^–^ that can promote a nucleophilic attack on carbon.^16,19^ So far, the literature on BP materials and configuration for AEM-WE is still limited, and, basically, no studies dedicated to AEM-WE operating with pure water have been reported yet. However, it can be argued that the material selection should follow the same criteria used for AEM-WEs fed with alkali-containing electrolytes. This hypothesis can be considered a conservative one.

The PTLs are located between the BP surface and the anode and cathode sides of the MEAs. They are complex components responsible for a variety of functions. First of all, PTL provides a proper connection between the MEA and the BP to create interfaces with a low contact resistance. Additionally, one of the primary functions of PTL is an even feed distribution while allowing rapid removal of the hydrogen and oxygen gases via the flow against the supplied electrolyte. Finally, the PTLs must be thermally conductive to allow safe thermal management of the MEA.^334,335^ The PTL is a critical component in determining WE performance and its structure should be optimized.^336^ Previously, PTL was extensively studied in PEM-WEs.^334,335,337,338^

A recent comprehensive review analyzed the PTLs in PEM-WEs and the effects of porosity and pore size distribution, pore gradient, thickness, and pretreatment on ohmic, mass transport, activation overpotential, and PTL modeling.^339^ Higher porosity enhanced the mass transport but lowered the charge transport. Conversely, low porosity decreases mass transport but promotes charge transport. Optimal porosity for the PTL needs to be found case by case; however, porosity in the range between 30% and 50% is suggested.^339^ Optimal porosity gradients are the ones with low porosity close to the CL and larger close to the flow field since they improve charge transfer and mass transport.^339^ Thickness is also a critical aspect to be considered: lower thickness increases the mass and charge transport; however, it reduces the stability of the PTL. The interface between the PTL and CL is also critical to lowering the contact resistance and enhancing performance.^339^ PTLs undergo passivation, lowering their electrical conductivity and enhancing ohmic losses. Therefore, a protective coating layer is usually applied to enhance the stability and durability of the PTLs. Finally, PTL modeling can enhance the understanding of the two-phase mass transport within the porous media.^339^

In the case of AEM-WEs, the architecture containing a microporous layer (MPL) was less explored despite much knowledge that can be inherited from the wider experience in PEM-WE. Further research efforts in this direction could lead to a significant improvement in the performance of AEM-WE.^336^ PTLs can also be used as a support for the active phases. It was shown that an AEM-WE with an EC deposited on a titanium nonwoven fabric modified by anodization is an excellent support for Pd-based ECs.^340^ Current densities of up to 2 A cm^–2^ were demonstrated in the study. Interestingly, the use of modified PTLs as electrodes could enable the fabrication of electrodes with embedded nanostructures that are easy to handle and limit the potential release of nanoforms at any stage of the device life cycle.^70,341,342^ The deposition of the active phases on the PTL can be performed using binder-free and solvent-free methods. Razmjooei et al. showed stainless steel PTL coated with NiAl at the anode and NiAlMo at the cathode using atmospheric plasma spray.^336^ It was shown that this approach is effective in obtaining three-dimensional electrodes with highly productive and cost-effective processes. However, the absence of alkali in the feed solution limits the conductivity and leads to resistance problems in extended electrode architectures, where active sites can be located far from the membrane. Obtaining appropriate surface conductivity either by surface functionalization of the materials and/or by the addition of ionomer might mitigate this issue and further research is required to confirm this hypothesis. In terms of materials, in alkaline environments, nickel-based PTL (e.g., nickel foam or felt) can be used at the anode.^337^ The Pourbaix diagram shows that nickel undergoes passivation under anodic conditions at high pH.^338,343^ The use of Ti at the anode is not recommended because titanium can dissolve at high anodic potentials and high pH, leading to stability problems. Stainless steel is also very promising and its use at both the anode and cathode was reported.^337^ The importance of selecting an appropriate anode PTL for AEM-WE operation (with 0.3 M KOH or 1 wt % K_2_CO_3_ electrolyte) was highlighted in the work of Hassan et al.,^295^ where two different PTLs, Ni foam and platinized Ti, were used. A thorough design-of-experiment study with full statistical analysis was made investigating the effect of four different variables: EC loading, EC type, PTL type, and addition of carbon to the electrode. The PTL was found to be the factor having by far the largest impact on the AEM-WE performance, although the other variables all had a statistically significant effect.

The type of PTL, namely a Ni foam vs a sintered Ti, was found to be very impactful on the AEM-WE performance in DI water operation, with ∼ 150 mV gap at 1 A cm^–2^ in favor of the sintered Ti PTL. When operating with a supporting electrolyte solution (1% K_2_CO_3_ or 0.1 M KOH) instead, both PTLs performed similarly, with a 10–20 mV gap at 1 A cm^–2^ in favor of the Ni foam. This reverse trend was explained by the partial contribution of the Ni foam PTL itself to the OER activity in the presence of supporting electrolytes.^295^

More recently, Hassan et al.^298^ investigated the impact of 14 different anode PTL materials from different manufacturers. The PTLs were classified in terms of material (Ni alloy and stainless steel) and fabrication method (sintered, fiber felt paper, sintered gradient). IrO_2_ EC was deposited onto the PTL via hand airbrushing, and the tests were done in an AEM-WE operated with 0.3 M KOH. The results demonstrated that Ni-based PTL showed ∼ 100 mV lower voltage than stainless steel counterparts in the same cell fabrication and testing conditions. Little impact was instead provided by the PTL thickness and by the fibers vs sinter PTL structure. A PTL porosity in the range between 35 and 40% provided the best performance.

Li et al.^344^ showed that using an innovative type of PTL based on titanium thin foil with ordered and controlled porosity, fabricated via lithography and mask-patterned chemical wet etching, enabled considerably improved AEM-WE performance compared to a commercial platinumized Ti foam. The innovative PTL was electroplated with gold. In that study, 1 A cm^–2^ at 1.8 V in a deionized water-fed AEM-WE was achieved, which is one of the highest performances recorded in operations in pure water. These outstanding results are attributed to the enhanced interfacial contacts and improved mass transport due to the well-tunable morphological features. These features include straight-through pore structure, even pore distribution, and precise pore size to achieve highly efficient permeation.^344^

It is evident that to improve the performance of AEM-WEs fed with pure water, more research should focus on MEA and electrode fabrication, since the CL structure, as far as the interface between PTL, ECs, AEI, and AEM, has a much higher impact on the performance than when the AEM-WE is operated with a supporting electrolyte solution. Similarly to BPs, not much research was done on PTLs specifically designed for AEM-WEs and even less literature is available for PTLs used in AEM-WE operating with pure water. However, it can be assumed that the operating conditions are reasonably similar or milder compared to systems fed with the alkaline solution as an electrolyte, justifying the use of the same materials. Conversely, A-WE operates with electrodes fully immersed in a liquid electrolyte, while AEM-WE best systems operate with a “dry-cathode”; therefore, the water management issues are different and need to be specifically investigated for this system. Much more effort has to be put in place into understanding the mass and charge transport within the PTL and the eventual use of an MPL identifying the effect of porosity, pore size distribution and related gradient, pore gradient, thickness, etc., on ohmic, mass transport, activation overpotential.

## 6 Effect of Water Composition on AEM-WE Performance

An important aspect of hydrogen production with pure-water electrolyzers is the water quality. Several sources of water can be used for electrolytic processes. The most common being groundwater, seawater, municipal tap water, and treated wastewater, or surface water. Currently, commercial WEs and most bench-scale pure-water electrolysis experiments use ultrapure water (UPW). Several standards for water purification can be found, but perhaps the most common is the American Society for Testing and Materials (ASTM) standard for reagent water (D1193), which specifies four levels of purification and their species contents (Table 3).^345^ From Table 3, there are four main subcategories to assess the water quality:

- Ions concentration
- Total organic content (TOC)
- Silica
- Bacteria

**Table 3:** Classification of Water Type and Maximum Contaminant Levels According to the ASTM Regulation^345^

	Type I	Type II	Type III	Type IV
Electrical resistivity, MΩ·cm, min (conductivity, μS cm^–1^)	18 (0.056)	1.0 (1.0)	4.0 (0.25)	5.0 (0.2)
TOC, max, μg L^–1^	50	50	200	no limit
Sodium, max, μg L^–1^	1	5	10	50
Chlorides, max, μg L^–1^	1	5	10	50
Total silica, max, μg L^–1^	3	3	500	no limit

The primary metric used to classify water purity for water electrolysis is electrical conductivity (or resistivity), which reports on the total ion concentration in the water. While A-WEs can handle UPW with a conductivity of <5 μS cm^–1^, AEM-WEs and PEM-WEs usually require higher purified water with less than 0.1 μS cm^–1^. To date, there is no established water purity standard for WEs. Despite significant research on the effects of impurities in AEM-WEs, no specific studies have focused on how varying water purity levels influence their performance and durability. This may be due to the broad range of AEMs and ECs used in AEM-WEs, unlike the more limited options available for PEM-WEs. Therefore, although it may be feasible to use less pure water with AEM-WEs, the prevailing assumption is to maintain the same purity level as in PEM-WEs due to the possible sensitivity of the membranes.

As a rule of thumb, a 1 MW electrolyzer requires about 200 L h^–1^ of UPW.^83^ Depending on the water source, the efficiency of purification is between 30% to 70%^346^ therefore, 280–660 L h^–1^ of raw water is required per 1 MW of water electrolyzer. In addition, more water is required for cooling, usually twice that of the UPW (about 400 L h^–1^).^346^ Hence, the relation between the WE capacity, the available water sources, and the contamination level should be carefully considered when choosing the facility location and the purification process.

In recent years, there has been an ongoing debate regarding the necessity of UPW for alkaline electrolyzers, which is expressed in an emerging field of EC development for direct seawater electrolysis.^64,66,83,94,347,348^ The main arguments in favor of direct seawater electrolysis are: 1) eliminating the step of water purification which reduces the facility footprint and the overall energy demands; 2) reducing the cost related to the water desalination; and 3) advantage of on-site hydrogen production from seawater, for example, coupled with off-shore renewable energy such as wind farms.

Electrolysis of seawater is a very challenging task, suffering from intrinsic limitations of low activity and durability.^349^ The pH of seawater is near neutral (around 8–8.5), which means that the concentration of H_3_O^+^ and OH^–^ is quite low, resulting in significantly lower electrocatalytic performance and higher resistivity, which causes an additional voltage drop in comparison to strong acidic or alkaline solutions.^350^ In addition, the buffering capacity of seawater is very poor. Hydroxide ions generation in the cathode and their consumption in the anode cause to increase in the pH in the former and a decrease in the latter. Over time, this difference is building up and pH shifting by 5 to 9 units has been reported.^94,351,352^ This causes severe degradation of the ECs and membrane, in addition to lower electrocatalytic activity. In most of the published works the electrolyte was not just seawater, but rather an electrolyte simulating seawater (usually 0.5 M NaCl which has the same salt percentage as seawater ∼ 3.5% wt. and the addition of some other salt for buffering),^353–355^ seawater with added salt for buffering (usually phosphate or borate)^356–358^ or seawater with the addition of KOH or NaOH.^359–364^ In the few cases where only seawater was tested, the ECs performance for either HER or OER was much lower than the one obtained with KOH solutions.^363,365,366^

Adding KOH to seawater helps in the stabilization of the pH and shows good electrocatalytic results, but at the same time, creates another severe problem, which is the precipitation of a hard scale on the electrodes and on the membrane.^367,368^ Considering the solubility product constant of many hydroxide complexes (Table 4), the maximum solubility of many cations that can be found in the raw water is extremely low. Assuming an overpotential of 100 mV at the cathode, the maximum solubility of selected metals was calculated based on their standard reduction potential and the Nernst equation. As can be seen, the maximum solubility is several orders of magnitude lower than their concentration in seawater. Those metals can be deposited on the cathode, causing blockage of the electrocatalytic sites and causing side reactions that result in lower activity and durability.

**Table 4:** Common Ion Concentrations in Seawater, Groundwater, and City Water and Their Maximum Solubility under Alkaline Conditions (pH 13)^a^

Ion	Seawater (μg L^–1^)	Groundwater (μg L^–1^)	City Water (μg L^–1^)	K_sp_ ^a^	Standard potential (vs SHE)	Max. solubility @pH13 (μg L^–1^)^b^
Mg	1.3·10^6^	5.1·10^3^	6.3·10^3^	5.61·10^–12^		2.31·10^–5^
Ca	4·10^5^	1.2·10^5^	2.6·10^4^	5.02·10^–12^		1.25·10^–5^
Ba	30	240	2000	2.55·10^–4^		1.86·10^2^
Mn	3.4	3.5	2.5	1.9·10^–13^		3.46·10^–7^
Zn	10	4.1	62	3·10^–17^	–0.76	4.58·10^–11^
Cd	0.11	0.08	5		–0.4	1.62·10^–16^
Cu	3	2	100		–0.34	1.0·10^–41^
Fe	10	92	20		–0.44	4.99·10^–15^
Ag	0.3	0.27			0.79	5.7·10^–29^
Ni	5.4	4.7	1.6		–0.25	1.8·10^–21^
Co	0.27	0.5			–0.28	1.11·10^–20^

a The values of the ion concentration in different water sources were taken from refs 369–371 . The values of the standard reduction potential and the K_sp_ were taken from refs 246 and 372. The maximum solubility for each ion at pH 13 was calculated.

b At 25 °C.

c At 100 mV overpotential at the cathode. The maximal solubility was calculated by rearranging the Nernst equation - $\left[X\right]=10^{\eta \cdot n/0.059}$ where n is the electron number, η is the overpotential, and [X] is the ion concentration in M.

Figure 19 shows the maximal solubility of different cations at pH 13 and the average seawater concentration (logarithmic scale). Those values were calculated by cross-referencing with the Pourbaix diagram of those elements to find the most stable phase under the given conditions. For example, the max solubility of manganese is 3.46 × 10^–7^ μg L^–1,^ whereas a typical concentration in seawater is 3.4 μg L^–1^, a 7 orders of magnitude difference.

**Figure 19:**
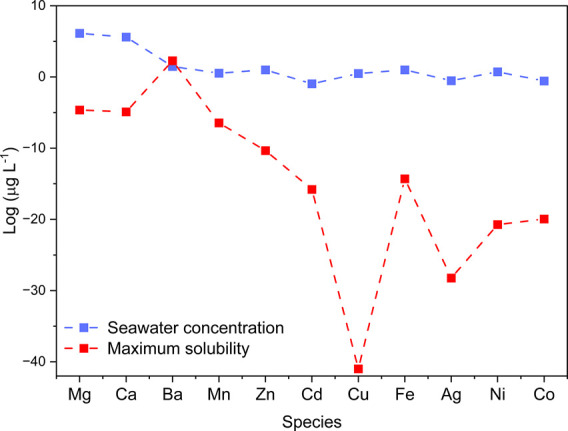
Logarithmic values of average concentration in seawater (blue symbols) and the maximal solubility at pH 13 (red symbols) for different cationic species.

Yu et al. demonstrated a synthesized 3D core–shell NiMoN@NiFeN tested for OER.^363^ After electrolysis in a mixture of seawater and 1 M KOH, a clear white deposition of insoluble salt on the electrode appears (Figure 20). The buildup of undesirable salt precipitate is critical in a cell with a constant flow of electrolytes.^86^ Additionally, the presence of metal ions such as copper, iron, zinc, cadmium, and more can result in the deposition of metallic particles on the cathode.^86^ This part will be discussed deeply later on in the review (section 6.3.1).

**Figure 20:**
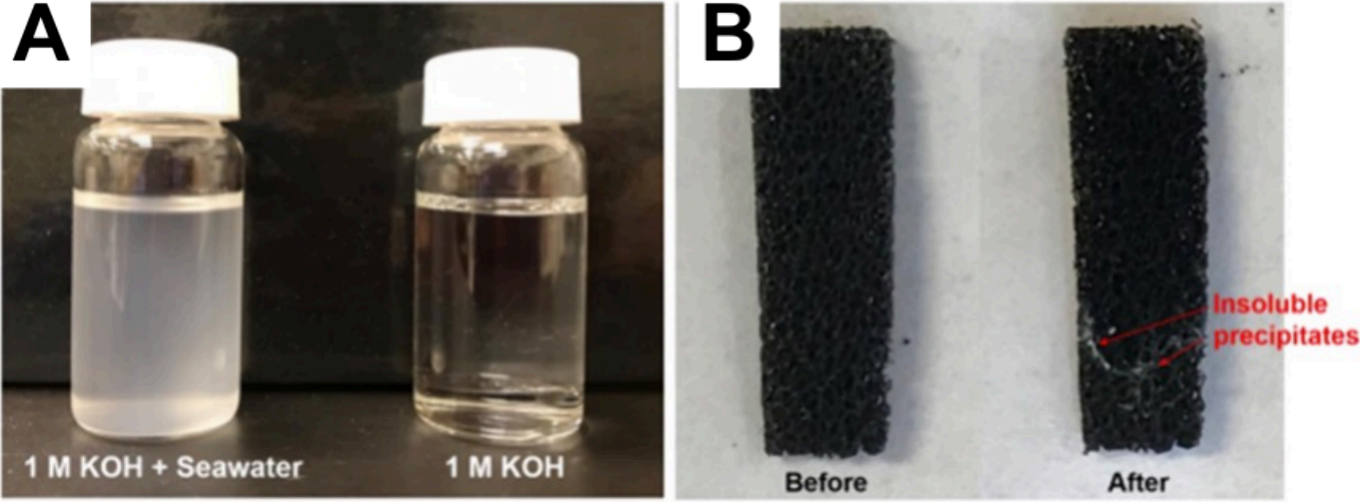
Optical images of (A) the two electrolytes and (B) the NiMoN@NiFeN sample before and after seawater electrolysis. Figures reproduced from ref 363. Copyright 2019. Springer Nature. Licensed under CC-BY 4.0.

At the same time, the most significant side reaction at the anode is the chloride oxidation reaction (COR), which can compete with the OER. An overall assessment of the Cl^–^ oxidation products in an AEM-WE should also consider the formation of OCl_2_^–^ and OCl_3_^–^.^67^ However, a mixture of ClO^–^, OCl_2_^–^ and OCl_3_^–^ is envisioned; therefore, the Poubaix of the three species is reported in Figure 21. Below are reported the Nernstian expressions (V vs SHE, ignoring activity effects) for the anode reactions involving ClO^–^ (eq 13a), OCl_2_^–^ (eq 14) and OCl_3_^–^ (eq 15).

14 $$E_{OCl^{-}/Cl^{-}}=1.72-0.059\times pH+0.059\log\left[OCl^{-}\right]$$

15 $$E_{Cl{O_{2}}^{-}/Cl^{-}}=1.59-0.059\times pH+0.059\log\left[Cl{O_{2}}^{-}\right]$$

16 $$E_{Cl{O_{3}}^{-}/Cl^{-}}=1.45-0.059\times pH+0.059\log\left[Cl{O_{3}}^{-}\right]$$

**Figure 21:**
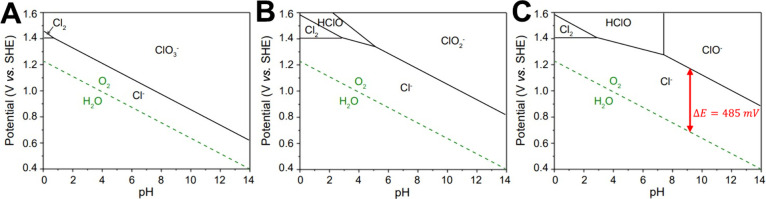
Pourbaix diagrams of H_2_O and (A) OCl_3_^–^, (B) OCl_2_^–^, and (C) OCl^–^ in 0.5 M Cl^–^ at 25 °C. Reproduced with permission from ref 67. Copyright 2020, Elsevier Inc.

OER shows the same pH dependency and the constant difference between the two is 485 mV at 25 °C at pHs above 7.5 (Figure 21C). At elevated temperatures (60 °C), this difference is further increased by 21.5 mV. Commercial A-WEs are usually working in the current density range of 0.2 to 0.8 A cm^–2^ and up to 2 A cm^–2^ with AEM-WE, which translates to an overpotential of about 500 mV and higher in the anode (based on the HER/OER exchange current density ratio). Being mechanistically simpler and kinetically faster than OER (2e^–^ vs 4e^–^), COR is a strong competitive reaction that should be avoided by engineering ECs that can suppress COR or by purifying the water.

Overall, direct seawater electrolysis is not studied enough to be considered for commercial application and additional in-depth research is needed to mitigate EC degradation, ion participation, ion poisoning, and side reactions such as COR. Significant attention is devoted to the EC degradation mitigation strategy, but deleterious effects can also occur on the AEM.^68^ Importantly, to allow a true assessment of direct seawater electrolysis and its potential, the tests should be conducted under the realistic condition of constant electrolyte circulation.^373^ The effect of impurities on the AEM-WE will be discussed deeply in the following sections.

### 6.1 Cost Analysis of Water Purification

The cost of water purification to UPW can have a significant impact on both CAPEX and OPEX. CAPEX for producing UPW may include the costs of purchasing and installing specialized equipment for water purification, such as reverse osmosis (RO) systems, deionizing (DI) systems, and other filtration methods.^374^ In terms of OPEX, the ongoing costs of operating and maintaining the water purification equipment must also be considered. OPEX is dominated by electricity costs and includes replacement parts, labor, and water usage.^346^ However, the benefits of using UPW in a water electrolyzer can be substantial, as it can help improve the efficiency and longevity of the electrolyzer cells, leading to increased production and reduced maintenance costs in the long run. Therefore, while producing UPW can have a significant financial impact, the benefits of improved efficiency and reduced maintenance costs may outweigh the initial expenditures over time.

In the literature, many techno-economic analyses show that the energy saving using direct seawater electrolysis is negligible, while the disadvantages are enormous and can lead to a reduced lifetime of the electrolyzer and to overall more complicated and costly operation.^16,94^ The actual cost of UPW production ($ m^–3^) is heavily dependent on the electricity (rates, renewable vs grid), location (water source, lands availability), purification specification (water quality), production capacity and as a result, a range of price estimations can be found.^346,375–377^ The most common procedure for producing UPW is pretreatment-RO-polishing, with RO being by far the most energy-intensive^346^ as shown in Figure 22.

**Figure 22:**
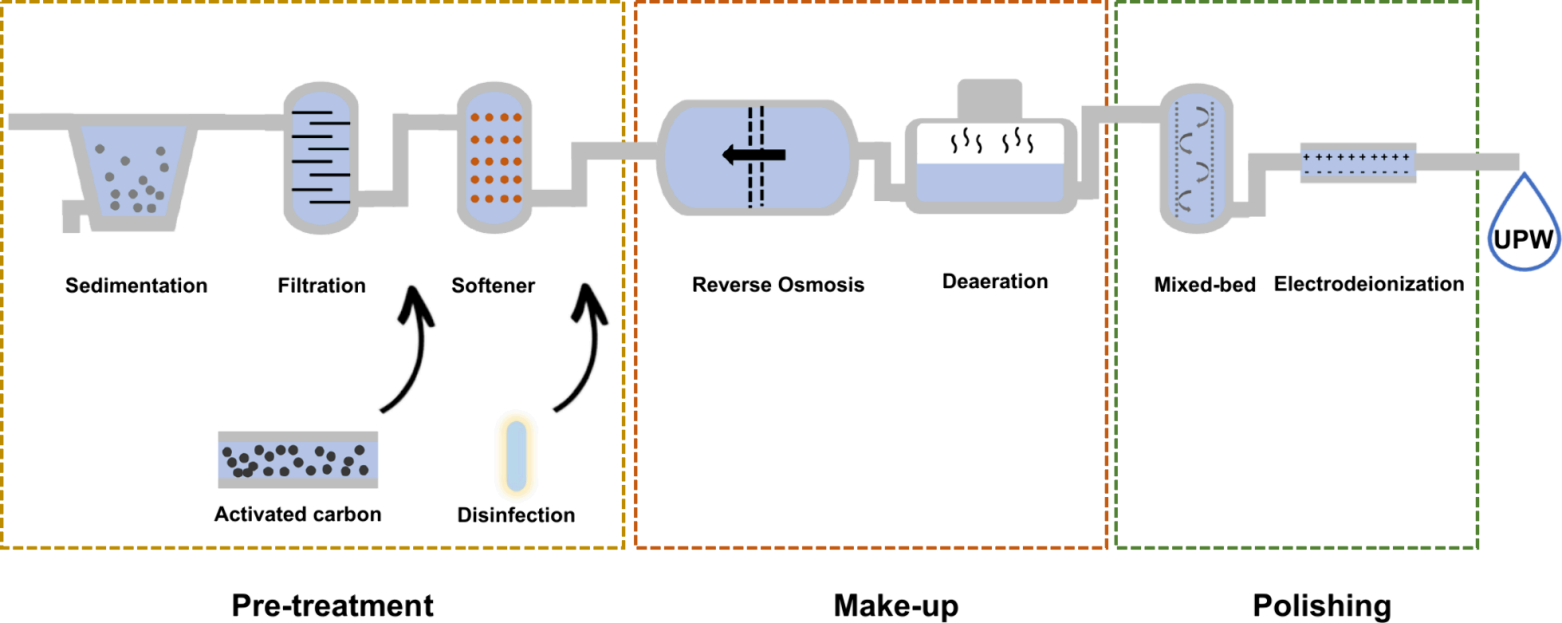
Schematic of a seawater desalination plant considering pretreatment, reverse osmosis and polishing.

RO removes the vast majority of the contaminations (>98%) and the energy demand of the polishing step (using electro-deionization (EDI) or capacitive deionization (CDI)) is about 5 to 10 times lower than the previous steps.^378,379^ Therefore, the overall energy demand and related cost of producing UPW are comparable to that of desalination (including the pretreatment).^16^ Hence, it is convenient and more realistic to consider the actual levelized cost of water (LCOW) of plans around the world for the electrolysis step. The last 10 years reported LCOW using RO ranges between 0.46 to 1.2 US$ m^–3^ and this cost is variable in function of the location, the water characteristics and the cost of electricity.^376,380–382^ Taking the average price for the total UPW to be ∼ 0.9 $ m^–3^ and the conversion ratio of 9 kg-UPW/kg-H_2_, the water purification process is at least 2 orders of magnitude less than the target price of 1$/kg-H_2_.

Accounting for thermodynamics and assuming 100% electrolyzer efficiency, the minimum energy requirement for electrolysis of 1 kg of pure water is 3.66 kWh. Even when considering the most demanding water source - seawater, the energy consumption for producing 1 kg of pure water is about 520 times less than the electrolysis process (∼0.007 kWh).^383^ In case the source water is groundwater, the energy consumption is less than a third of seawater.^374^

Realistic WEs are working with much less efficiency - about 65% (alkaline) to 80% (PEM). Taking the average efficiency for AEM-WE to be 73%, the energy requirement difference between the electrolysis and purification process is at least 3 orders of magnitude. In addition, water purification technologies are getting better over time, which will be expressed in lower energy demands and overall lower LCOW. For small-scale WEs, where the water uptake is relatively low, the desalination process can have a more significant impact on the overall cost. Small-scale WEs are usually located close to the consumer (factory, for example) for on-site hydrogen production. In this case, the water source can come from municipal water, which can minimize the energy requirement during the RO step (in comparison to seawater) or even eliminate it, relying only on EDI or CDI.

### 6.2 Effect of Electrolyte Composition and Pure Water Feed on AEM-WE Performance

The composition of the circulating electrolyte is yet another essential factor in optimizing the performance of the WE. Supporting liquid electrolytes, often comprising hydroxide or carbonate/bicarbonate salts of alkali metals, are employed to uplift the performance. Supporting liquid electrolytes being capable of facilitating the distribution of reactant hydroxide across the CL helps in plummeting the ohmic resistances and enables the EC/electrolyte interface, together with an improved ECSA.^384^ AEM-WE requires relatively less concentrated alkaline liquid electrolyte to rationalize the kinetic performance, whereas the usage of pure water to simplify the BoP remains challenging.^385^ There is literature showing cell performance in both pure water and KOH-supporting electrolytes,^57,255,386–388^ but there are not many studies systematically comparing the effects of pure water vs KOH-supporting electrolytes, and it is hard to summarize in simple metrics. Presumably, the EC layer design, EC-ionomer interface, ionomer/membrane, and other cell conditions have different impacts on the cell performance and durability with pure water and KOH as a feed; thus, optimizing these parameters would focus on different directions. Here, some examples of AEM-WE cell operation with a supporting electrolyte as opposed to pure water feed are introduced.

As already discussed, AEM-WE are usually fed through anolyte (solution fed at the anode) alone while keeping the cathode dried during the process, and thus water is supplied to the cathode via diffusion from the anodic side for the execution of HER and to avoid ionomer dehydration.^289^ Since the feed of pure water badly affects the performance due to high overpotentials and limited ionomer durability,^389^ the usage of secondary supporting electrolytes becomes inevitable. Recently, Kiessling et al. launched a detailed investigation to analyze the role of supporting anolyte feed on the overall performance as previously conceptualized in Figure 9.^170^ Using Tokuyama AEMs, the authors first ran the electrolyzer with 1 M KOH anolyte, which demonstrated the optimum performance; however, a successive circulation of Milli-Q water (DIW) as an anolyte irreversibly damaged the polarization curves with significantly higher HFR and polarization resistance. Moreover, while examining the type of cations present in the anolyte, Kiessling et al. experienced Li^+^ and Na^+^ causing voltage decay and degradation due to the OH^–^ stabilization effect.^170^ Cations tend to stabilize the OH^–^ ions by making noncovalent interactions, which restrict their mobility within the interfacial-double layer and hence OER activity gets worse.^390^ As the stabilization energy is governed by the charge density of the alkali metal cations in the order of Li ^+^> Na^+^ > K^+^, electrolytes containing K^+^ cations are considered more suitable.^170^ The cell potential and the HFR do not depend uniquely on the anolyte conductivity. In fact, when CO_3_^2–^ was used instead of OH^–^ (e.g., K_2_CO_3_ vs KOH), the kinetics of the reaction were worse due to an increased cation-to-OH^–^ ratio.^170^ Based on the results of their work, the authors recommended the use of pure (deionized water, DIW) and concentrated KOH solutions as the best-supporting electrolyte for AEM-WE operation, due to the lack of side reactions or other factors related to intermittent operation. If the use of an electrolyte with a reduced pH is needed, CO_3_^2–^ ions are recommended in case of operation at a steady state and high current density. This conclusion was drawn from comparing the HFR of AEM-WE operated with KOH and K_2_CO_3_ solutions with the same ionic conductivity (0.5 M KOH and 0.82 M K_2_CO_3_). It was found that at low current density (0.1 A cm^–2^) the HFR with K_2_CO_3_ was much higher than with KOH (175 vs 75 mΩ cm^2^), while at high current density (1 A cm^–2^) the HFR values were the same. This was explained by the so-called “self-purging effect” occurring at high current densities: CO_3_^2–^ anions are removed from the membrane by the high flux of OH^–^ ions flowing from the cathode to the anode. CO_3_^2–^ anions are then removed at the anode by the formation of CO_2_ according to the following reactions.^170^

17 $${{\mathrm{CO}}_{3}}^{2-}+H_{2}O\to {{\mathrm{HCO}}_{3}}^{-}+{\mathrm{OH}}^{-}$$

18 $${{\mathrm{HCO}}_{3}}^{-}+H_{2}O\to H_{2}{\mathrm{CO}}_{3}+\mathrm{OH}$$

While comparing different types of conventional AEMs, Pushkareva et al. reported favorable performance at various KOH concentrations, whereas the utilization of pure water led to a stark increase in membrane resistance and hence declared it insufficient for efficient electrolysis.^71^ With the circulation of 1 M KOH at 60 °C through both anodic and cathodic counterparts, all the analyzed AEMs, including Sustanion, Aemion and A201 exhibited noticeable current densities of 2 A cm^–2,^ nearly at 2.13 V, 2.26 V and 2.21 V, respectively. No doubt KOH could be expensive when compared to NaOH, but its higher conductivity, lower viscosity, and considerably greater solubility of potassium carbonate than Na_2_CO_3_ make it a suitable candidate for electrolysis.^14,19^

The ionic mobility to complete the redox reaction is determined by the solution conductivity, where pH expresses the alkalinity of the solution. Therefore, Ghoshal et al. observed the impact of the pH of the supporting electrolytes on the integrated membrane/ionomers and found a significant loss in the MEA performance with a subsequent decrease in pH, probably due to a slump in conductivity.^391^ As the pH drops, the concentration of hydroxide ions also decreases, which affects the kinetics of OER taking place at the anode.^392^ The mechanism behind the improvement of AEM-WE performance was further explored by Liu et al. by combining the experimental study with mathematical modeling.^252^ The cell voltage measured at 0.4 A cm^–2^ was ∼ 1.6, 1.7, and 1.9 V in 1.0 M KOH, 0.1 M KOH, and DI water, respectively. They witnessed a 5-fold expansion in ECSA with 1 M KOH compared to DI water, which utilizes just ∼ 16.7% of the EC surface. Figure 23.A-B signifies the dependence of cell performance on pH/concentration of KOH electrolyte, where the overall activity in both regions of kinetics and mass transportation boosts as the concentration increases, while HFR gets doubled when the electrolyte is switched to DI water from 1 M KOH. As already illustrated in Figure 16A, when the KOH electrolyte circulates, additional conducting pathways (indicated by red arrows) get established, increasing the ECSA at liquid electrolyte/EC interfaces with minimization of kinetic losses, which is unlikely with DI.^252^ Such results further underlined how creating an optimal CL structure via proper formulation, maximizing the ionomer-EC interface, can enhance performance in AEM-WE operating with pure water feed.

**Figure 23:**
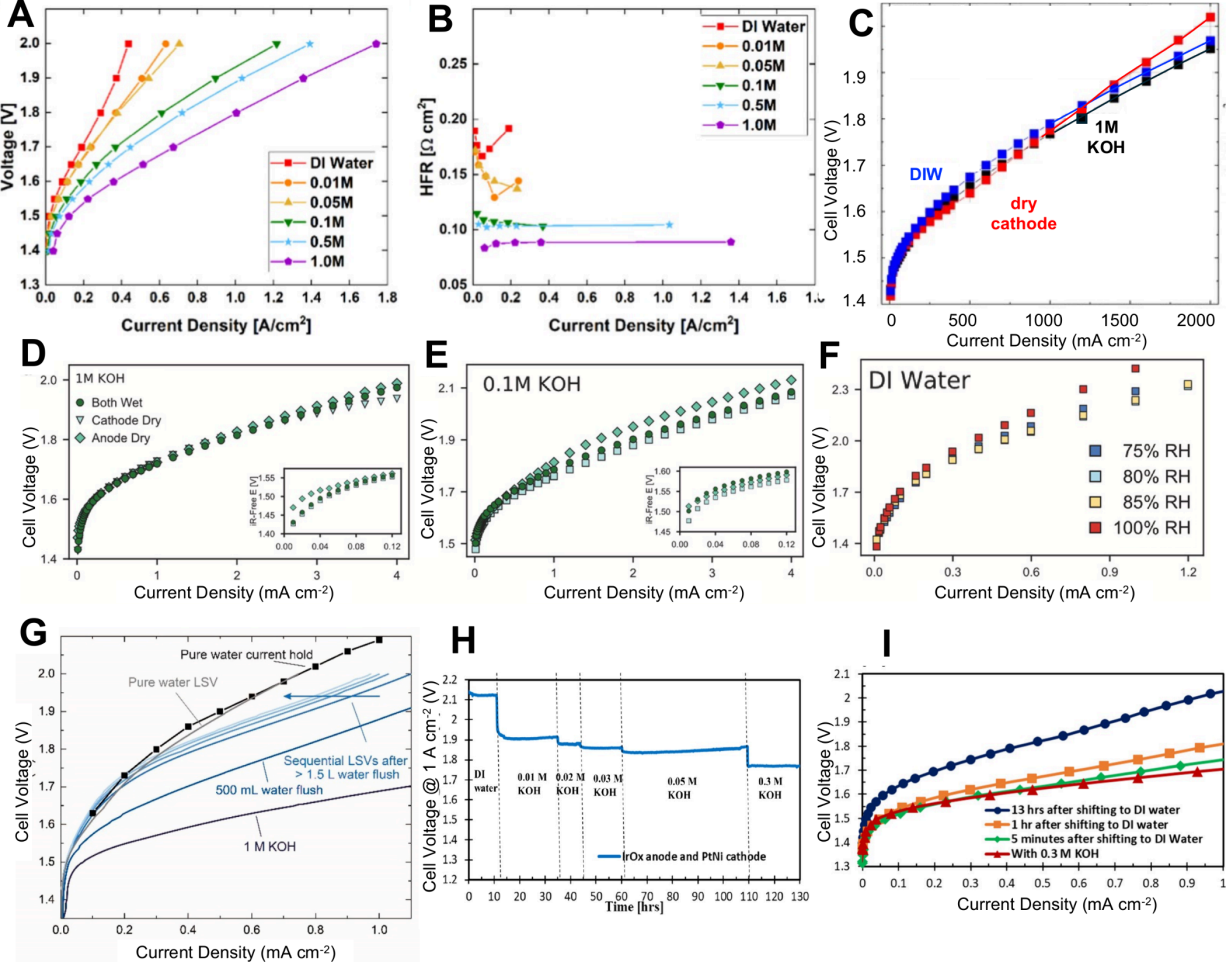
Polarization curves when AEM-WE fed with anolyte while keeping the cathodic counterpart dry (A) and HFR (B) as a function of KOH concentration. In the study, cathode and anode ECs were PtRu/C and IrO_2_, respectively, where HTMA-DAPP was employed as a membrane. (A-B) reproduced from ref 252. Copyright 2021, IOP. Licensed under CC BY-NC-ND 4.0. (C) Polarization curves (kinetic zone in inset) of AEM-WE operated with 1 M KOH anolyte and with different catholyte conditions: DI water, 1 M KOH, and dry with no catholyte flow. (C) reproduced from ref 394. Copyright 2022, IOP. Licensed under CC BY-NC-ND 4.0. AEM-WE polarizations comparing the different feeding conditions containing (D) 1 M KOH, (E) 0.1 M KOH, (F) pure water. (D–F) reproduced from ref 175. Copyright 2023, Elsevier B.V. Licensed under CC-BY 4.0. Comparison of polarization curves at various points when conditioning in 1 M KOH (G). Reproduced with permission from ref 56. Copyright 2021, American Chemical Society. AEM-WE operating at 1.0 A cm^–2^ and 60 °C, where the initial feed of pure DIW was incrementally switched to KOH (H). The cell was configured with IrOx OER and PtNi HER ECs having GT72–10 AEM. Polarization curves were obtained as the cell operation started with 0.3 M KOH and then at different times after switching the system to DIW feed (I). (H–I) are reproduced with permission from ref 398. Copyright 2022, IOP. Licensed under CC BY-NC-ND 4.0.

In addition to hydroxide-containing supporting liquid electrolytes, carbonate-based electrolytes with relatively milder alkalinity are sometimes also used to favor the stability of MEA and operational safety.^19,72,384,393^ A comparison of operation with DI water and with different concentrations of K_2_CO_3_ (between 0.1 and 10 wt %) in the solution fed to the anode was reported by Ito et al.^72^ As expected, much better performance was achieved with higher electrolyte concentrations. The voltage gap measured at 0.1 A cm^–2^ between DI water and the lower K_2_CO_3_ concentration (0.1 wt %) was as high as 0.6 V. The cell resistance measured in these two conditions was ∼ 1.5 and ∼ 0.2 Ω cm^2^, for water and LE, respectively, explaining the large performance gap observed.^72^ On the other hand, Vincent et al. observed comparable results for 1% K_2_CO_3_ and 1 M KOH, while the least potential was noticed particularly with 1 M KOH electrolyte, but its corrosive nature could affect the integrity of the membrane over an extended period of time.^392^ Furthermore, Pavel et al. compared the AEM stability with K_2_CO_3_/KHCO_3_ and K_2_CO_3_ and found appreciable durability with a minimal difference between them.^393^ In fact, the K_2_CO_3_ electrolyte initially had a slightly higher conductivity and pH and thus performed slightly better in the beginning, but soon it underwent carbonation and started behaving identically to K_2_CO_3_/KHCO_3_. With a similar thought, Hnát et al. systematically studied the influence of KOH substitution with Na_2_CO_3_ and NaHCO_3_ on performance and stability.^385^ As predicted, KOH clearly outperformed the other two candidates due to the reduced conductivity of both the liquid and the polymeric electrolyte, together with the diminished kinetics of the anode reaction. With the help of mathematical modeling, Stanislaw et al. attempted to elucidate the reason behind the decline in the performance upon using carbonate electrolytes.^73^ Their study moved the notion that conductivity loss due to the replacement of hydroxide with carbonate in the membrane has a minor impact on the overall device performance, but perhaps due to the shifting of carbonate ions with the OH^–^ generated by the cathode. Conversely, reduced hydroxide content in the anodic ionomer gives rise to a penalty in the Nernstian voltage. However, during operation, the carbonates adversely pile up across the PTL/CL, leading to the insufficient utilization of EC and constraining the OER reaction closer to the membrane edge.

Owing to a strong electro-osmotic drag in alkaline conditions, water molecules also travel toward the anode from the cathode.^72^ In such a scenario, HER may undergo diffusion limitations and there is also a chance of cathodic ionomer drying out,^170^ while catholyte (solution fed to cathode) feed may help in overcoming these issues. Cho et al. systematically investigated the effect of the catholyte feed method on the overall performance of the electrolyzer while keeping the anolyte always 0.5 M KOH.^389^ Their study concluded with an assertion that alkaline catholyte feed is distinctively better than DI water feed, owing to enhanced utilization of EC surfaces of HER, together with improved ionic conductivity. However, higher OH^–^ content delivered by KOH prefeeding recovers the electrode kinetics and, in due course, improves the AEM-WE performance. Eventually, with optimal content of anode binder, operation under dry cathodic conditions after preliminary solution feed brought about the water splitting current density of 1.07 A cm^–2^ at 1.8 V. In another study, Kiessling et al.^394^ fully investigated the effect of the catholyte when the anolyte used was 1.0 M KOH. The results showed that feeding DI water or KOH solution at the cathode was beneficial at high current densities to avoid membrane dehydration and consequent HFR increase. At low current densities, instead, the presence of ions decreased HER kinetics, and better performance was obtained with dry cathode or DI water flow (Figure 23.C). Using a carbonate-containing catholyte negatively affects the performance due to anion poisoning of the HER EC. Similarly to the previous anolyte study, also in this case at low current density with the 0.82 M K_2_CO_3_, the HFR value was much higher than with KOH (even at a much lower concentration of just 18 mM KOH), but at high current density, the HFR decreased due to the self-purging effect. However, poor HER kinetics in the presence of a carbonate-containing electrolyte compared to OH^–^ containing ones explained the lower performance at all current densities observed for the K_2_CO_3_-fed AEM-WE.

Tricker et al.^175^ compared the AEM-WE operation with 1 and 0.1 M KOH with a dry cathode (feed at the anode only), double-feed and dry anode (feed at the cathode only), and better performance was obtained for the dry cathode operation (Figure 23.D-E). These results suggest that water back-diffusion from anode to cathode is sufficient to ensure enough reactant for the HER at the cathode, even at current densities as high as 4 A cm^–2^ and with a 60 μm thick membrane. The same authors also investigated an AEM-WE feed with DI water at the anode where the cathode was fed with an H_2_ gas flow at different levels of humidification, showing that better performance was obtained with a relative humidity (RH) value of 80% than with 100% (Figure 23.F). This was explained by the existence of a higher water gradient from the anode to the cathode, favoring the water back-diffusion. At a lower RH value of 75%, the performance decreased due to membrane dry-out. The RH impact was higher at higher current density.

Oliveira et al.^395^ justified the better performance typically obtained with feeding only the anode by suggesting that AEM contamination from the formation of CO_3_^2–^ and HCO_3_^–^ ions due to dissolved CO_2_ occurs, lowering the ionic conductivity. Since water can diffuse easily through the AEM, it can be fed as an anolyte. In this way, all the anions are concentrated on the anode side, where CO_3_^2–^ and HCO_3_^–^ ions can be easily self-purged as described before, without having to flow through the AEM, so explaining the superior anode-feed AEM-WE performance.

The ultimate goal of the AEM-WE technology is to be operated viably with pure water for competitive hydrogen generation. However, this concept is still in its early phase and requires significant scientific findings to overcome the technical drawbacks of the system. In addition to the aforementioned problems associated with DI water, i.e., high resistances and low ionic conductivity leading to kinetic losses, there are several other factors yet to be addressed. According to the perspective communicated by Li et al.,^282^ when AEM-WE operates with DI water alone without supplementary electrolyte, the ambient is less corrosive and the durability aspects are not dictated by the stability of MEA but, linked to a high operational voltage and current density of the cell causing ionomer detachment and poisoning. On the other hand, Mayerhöfer et al.^290^ analyzed the operational dissolution of the state-of-the-art CuCoO_*x*_-based OER EC after integrating it with the Aemion membrane and binder. Their study suggested that Aemion is not enough to develop a satisfactory alkaline environment to thermodynamically stabilize the EC in a neutral electrolyte since both Co and Cu dissolve during OER and, therefore, a feed of alkaline nature becomes vital to safeguard the thermodynamic stability of such kind of PGM-free ECs. Being aware of this issue, Xiao and co-workers fabricated fluoride-incorporated, nickel–iron oxyhydroxide (Fe_*x*_Ni_*y*_OOH-nF) based self-supported OER electrode, which was capable of robustly holding ionomer with high IEC without getting severely washed out during the operation.^315^ With pure water feed, they were able to achieve a remarkable current density of 1.02 A cm^–2^ at 1.8 V with subsequent prevention of EC detachment over 160 h of nonstop run at 0.2 A cm^–2^. Not long ago, Xu et al. studied the application of (oxy)­hydroxide/oxides of first-row TMs ECs for OER applications in AEM electrolysis.^249^ Despite having the best performance in an alkaline environment, NiFeO_*x*_H_*y*_ turned out to be the least-performing EC when tested with AEM under pure water feeding. NiFeO_*x*_H_*y*_ exhibited a large overpotential (above 2.5 V) to obtain the current density of 0.2 A cm^–2,^ whereas NiCoFeO_*x*_ even outperformed the IrO_*x*_ by delivering the 1 A cm^–2^ current density at ∼ 2.5 V. In fact, nickel (oxy) hydroxides-based ECs with electrolytic permeability are good conductors only in oxide form.^221^ However, in pure water, the EC present only at the ionomer interface can undergo oxidation, where the bulk EC stays unoxidized and is thus unproductive to drive OER due to loss of electrical conductivity.^249^ Another problem associated with DI water circulating AEM-WE is the ingression of CO_2_ into the system, which severely damages performance.^396^ Owing to limited mobility, their intrusion into the system can swiftly affect the ionic conductivity of both binders and membranes.^397^

In the pursuit of pure water-fed AEM-WE, where a situation of uncertainty prevails due to afore-discussed problems as well as different techniques used for the fabrication of cell components, the lack of a testing protocol also constitutes an additional bottleneck. Sometimes various electrolytic pretreatments are employed, even if the electrolyzer truly has to be operated with pure water.^56,57,181,313,398^ The procedures used to switch the system from KOH to DI water are generally obscure and the effects of residual salts on the overall electrolysis remain unclear. Lindquist et al. first noticed that and thoughtfully argued that the residual electrolyte may influence the performance of the final electrolysis, also leading to uncertain and complex degradation mechanisms involved in the ionomer.^56^ They compared the AEM-WE performance measured initially with DI water and then with 1.0 M KOH. Following what was done in other works reporting high AEM-WE performance in DI water, the authors measured the performance again after flushing the cell with 0.5 L of DI water, showing that it was significantly better than the initial one in DI water. The performance kept decreasing after flushing the cell with higher volumes of DI water, up to more than 2.5 L as shown in Figure 23.G. The authors also measured the cell effluent conductivity during the flushing, observing a decrease over time. Therefore, the enhanced performance measured in DI water after the test with 1.0 M KOH should be attributed to the residual electrolyte not being completely removed from the MEA.^56^ Very recently, Hassan et al.^398^ revealed that to truly switch from alkaline electrolyte to DI water feed, a large volume of water has to be circulated over an extended time to get rid of the residual salt and achieve the true performance associated with the use of DI water. To prevent misinterpretation of any artifical improvements in the performance due to trapped KOH, Hassan et al.^398^ adopted a reverse strategy where the device was fed with DI water at the start and then replaced with KOH feed with successive increments in the concentration as depicted in Figure 23.H. With each increment in the KOH concentration, the cell performance was improved, however, by the final feed of 0.3 M KOH, the operating voltage of ∼ 1.75 V was achieved which was 350 mV lower than that of DI water. To further probe the impression of residual KOH, the cell was initially operated with 0.3 M KOH and then switched to DI water. The succeeding polarization curve remained nearly identical to that initially taken with 0.3 M KOH and even after 1 h of continuous DI water flushing, the kinetic region came out to be somewhat similar to the previous polarizations (see Figure 23.I). It is noteworthy that it took 13 hours of continuous DI water purging to achieve the polarization originally attributed to it. Osmieri et al.^294^ confirmed this issue, measuring the AEM-WE performance of two different MEAs in DI water at the beginning of the test and after 20 h of constant current hold, showing significant degradation. Subsequently, the performance was measured in the presence of supporting electrolytes (in sequence, 1% K_2_CO_3_ and 0.1 M KOH), showing the expected performance improvement. Then, the cell was purged with 2 L of DI water and the performance was better than at the beginning of the test, indicating residual presence of supporting electrolyte.

All these works suggest that in order to enable a fair comparison of results obtained in different laboratories, it is of paramount importance to establish a protocol for AEM-WE cell conditioning and performance measurement, in case DI water and different supporting electrolyte solutions are used in sequence when testing the same MEA. Table 5 summarizes the electrolytic feed in the different AEM-WEs.

**Table 5:** Overview of Varying Electrolytic Feeds

Anode EC	Cathode EC	Binder/Ionomer	AnodePTL	Cathode PTL	Memb.	Temp.(°C)	Electrolyte	Cell Volt. (V)	Current Density (A cm^–2^)	ref.
etched copper–cobaltoxide	Pt/C	PTFE	Ni foam	carbon cloth	QPC-TMA	70	1 M KOH	1.9	4.2	^400^
spinel CuCo_2_O_2_	Pt/CHISPEC 4000	No	Ni foam	carbon cloth	XC37-50 grade T	45	1 M KOH	1.9	1.4	^240^
IrO_2_	Pt/C	PTFE	-	Ti paper	A201Tokuyama	50	0.5 M KOH	1.8	1.07	^389^
non-Pt-containing lead ruthenate pyrochlore	Pt Black	PSF–TMA^+^	-	Carbon paper	PSF–TMA^+^	50	Ultrapure DIW	1.8	0.4	^396^
CuCoO_*x*_	Pt/CHiSPEC9100	Aemion	Ni felt	carbon PTL	Aemion AP1-HNN8-50	70	0.1 M KOH	1.8	∼1 (not specified)	^290^
Copper cobalt oxide NPs	Pt/C HiSPEC4000	Nafion	Ni foam	carbon cloth	X37-50 grade T	50	1 M KOH	1.8	1.54	^401^
NiFe	PtRu/C	quaternized ammonium polystyrene	-	platinized titanium or SGL 29 BC	HTMA-DAPP	85	Pure Water	1.8	2.7	^181^
NiCoOx:Fe	Pt Black	FAA-3	Pt-coated sintered titanium frit	Carbon paper	FAA-3	50	Pure Water	2.47	1	^249^
FexNiyOOH-nF	Pt/C	PAP-TP-85	self-supported on Ni foam	Carbon paper	PAP	90	Pure Water	1.8	1.02	^315^
Ce_0.2_MnFe_1.8_O_4_	Ni powder	AAEM	(not specified)	(not specified)	Fumasep FAA-3-PK-130	25	DIW	1.8	0.3	^402^
CuCoOx(Acta 3030)	Acta 4030	I2	Ni foam	CarbonPaper	A201Tokuyama	60	1% K_2_CO_3_	1.95	0.5	^392^
Ni_0.75_Fe_2.25_O_4_	Pt/C	Nafion	-	Ni foam	X37-50 grade T	50	1 M KOH	1.9	2	^232^
IrO_2_	Pt/C	FAA-3–50	-	C-PTL &Ti-PTL	FAA-3-Br	70	1 M KOH	1.9	1.5	^310^
Ni_0.6_Co_0.2_Fe_0.2_	Ni–MoO_2_	Nafion	-	Au-coated Ti felt and carbon paper	Fumion	50	1 M KOH	2	1.15	^403^
IrO_2_	Pt/C	PFTP-8/PFBP-14	-	Carbon paper &Ti felt	PFTP-13	80	1 M KOH	2	7.68	^404^
NiCo_2_O_4_	Pt/C	QPPO	-	Ti fiber felt and C PTL	QPPO	40	0.1 NaOH	2	0.814	^405^
Ni-IrO_*x*_-400	Pt/C	10 wt % FAA-3	-	Ni foam	FAA-3-50	50	1 M KOH	1.8	1.125	^406^
NiFeV LDH	Pt/C	-	-	Ni foam	X37-50 grade T	50	1 M KOH	1.8	2.1	^407^

The problem mainly lies in the sequence of the AEM-WE performance tests carried out using different supporting electrolyte solutions (mainly a KOH solution) and pure water. By deeply reviewing the available literature, it can be noticed that this aspect is most of the time overlooked. For example, in many papers featuring pure water operation, experimental details that would be essential to properly determine if the claimed “pure water operation” is indeed true are omitted. In our vision, this omission is so common because the main focus of the majority of the research groups publishing papers in the AEM-WE field has so far been the development of one of the core components of the MEA (i.e., OER or HER ECs, AEMs, AEIs), not the testing protocol or the assessment of AEM-WE performance (especially in pure water) in a comparative way. In our opinion, the essential experimental details to be reported to enable a fair assessment are: (*i*) the exact sequence order in which tests are conducted when the same AEM-WE (i.e., the same MEA) is fed with a supporting electrolyte and with pure water; (*ii*) some indication that the cell has been purged with a sufficient amount of pure water (i.e., any residual electrolyte has been rinsed off from the MEA) before starting the “pure water operation” test in case the AEM-WE was previously fed with a supporting electrolyte solution (Figure 24). In our opinion, point (*ii*) above can be addressed by providing in the experimental section of the paper the value of the electrolyte conductivity at the outlet of the electrolyzer cell, or the exact volume (flow rate, time) of pure water flushed through the cell to wash out the residual supporting electrolyte. To avoid this problem and eliminate any concerns regarding the validity of the reported “pure water operation” performance, we recommend carrying out all the “pure water operation” experiments before flowing any electrolyte into the cell, as done, for example, in references^399^ and.^56^

**Figure 24:**
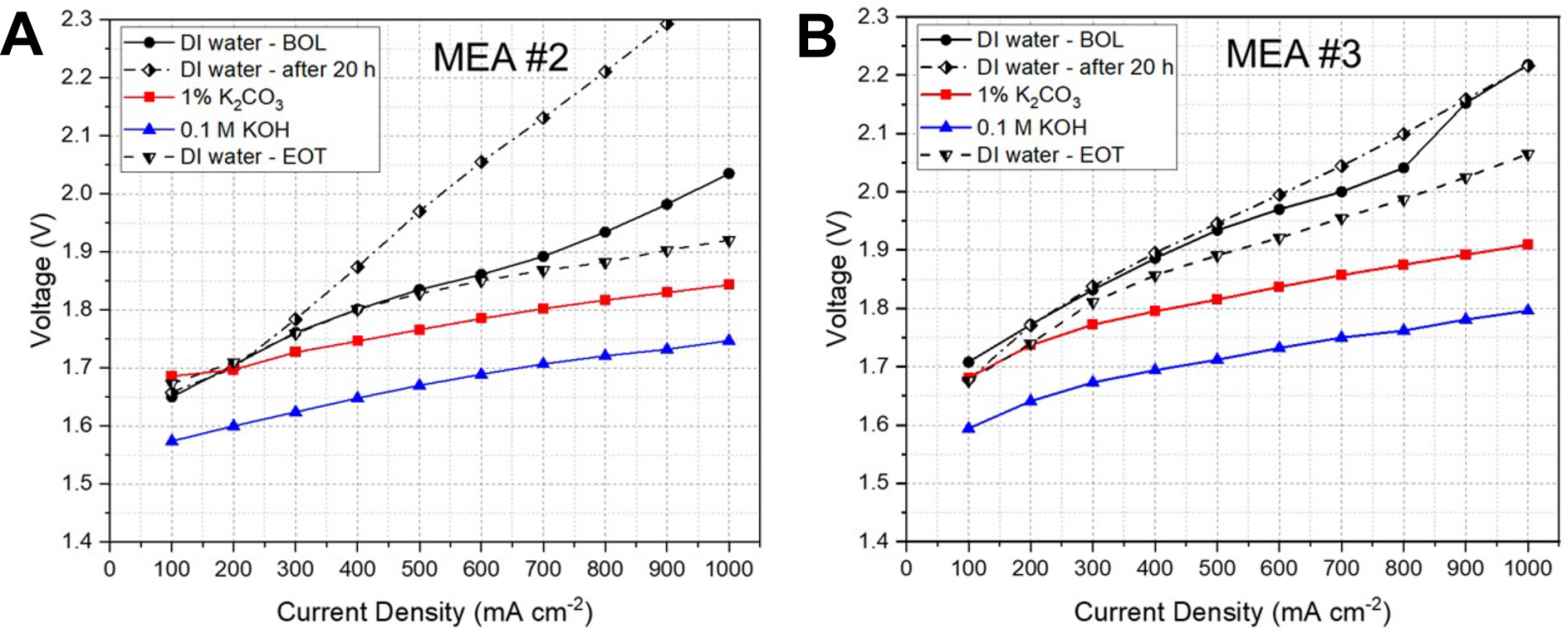
Polarization curves recorded in sequence in this order with different feeds: pure water at the beginning of life (BOL) and after 20 h durability test, 1% K_2_CO_3_, 0.1 M KOH, and pure water again at the end of the test (EOT) after flushing more than 2 L of pure water. Reproduced with permission from ref 294. Copyright 2023, Elsevier.

### 6.3 Effect of Impurities

The effect of impurities on ECs in the context of pure-water-fed AEMWE operation is a seriously understudied area of research likely stemming from a poor understanding of the chemical components of water feeds in operating electrolyzers.^86^ As a result, it is not clear to what degree the removal of impurities enhances or negatively affects EC performance. On one hand, there are very low amounts of dissolved species in the ASTM Type II water used in WEs: a maximum of 5 μg L^–1^ Na^+^, 5 μg L^–1^ Cl^–^, 3 μg L^–1^ silica and 50 μg L^–1^ TOC and whatever ions are remaining which constitute the 1 μS cm^–1^ maximum conductivity.^86^ This significantly reduces poisoning and fouling compared to lower-quality water sources. However, it is precisely this lack of ionic content that makes ultrapure water sources corrosive to components of the WE plant and may permit the leaching of species into the water stream. Pourbaix diagrams, which delineate regions of thermodynamic stability of an element according to potential, pH, and concentrations of soluble species, show that corrosion processes become more spontaneous as the concentration of the soluble species decreases.^408^ This may explain the corrosion of even stainless steel in ultrapure water.^409^ Indeed, for systems where the water flow is continuously purified, a scenario where the solution is never saturated with respect to the given corrosion product is likely and could lead to continuous leaching of impurities.

Impurities are not necessarily damaging to EC performance. For example, many common PGM-free ECs for OER display increased activity^223,410–412^ and stability^217^ only when Fe impurities are in solution. There may be other scenarios where impurities can inadvertently provide advantages to ECs. How different metallic and nonmetallic species interact with ECs is discussed below. The lack of information about such processes in pure water necessitates that the discussion focuses on research in alkaline electrolytes. However, this still should provide ideas about how to translate that research to the pure water context. Also, the discussion below will focus on inorganic impurities, while the effect of organic contamination is beyond the scope of this review.

#### 6.3.1 Metallic Impurities

Extensive research has been conducted on metal oxides with perovskite and spinel structures as well as layered structures such as M­(OH)_2_/MOOH (M = Ni, Fe, Co, and Cu), due to their structural stability and outstanding performance as ECs for OER. Perhaps the most studied PGM-free ECs for OER are nickel-based, mainly as LDH. Under high pH and potential, the nickel, at least at its surface, transforms into the oxide of oxy­(hydroxide) form, which is known to be highly active toward OER. As a consequence, in recent years, the development of nickel-based ECs has focused mainly on the improvement of the highly active Ni­(OH)_2_/NiOOH form, which shows record-high catalytic performance. In rigorously Fe-free conditions. However, a dramatic increase in activity is always observed upon the incorporation of iron.^413^ The first observations were made for nickel metal hydride batteries, where the presence of iron impurities caused a decrease in the battery capacitance, which was attributed to a decrease in the onset potential for OER at the anode.^414,415^ Trotochaud et al. showed unambiguously that the high catalytic activity of Ni­(OH)_2_/NiOOH is due to the incorporation of iron impurities from the KOH solution and that the increase over time of the electrocatalytic activity (aging) is not due to phase transformation from γ-NiOOH to β-NiOOH.^223,416^ Even though an increase of more than 30-fold in conductivity was observed with iron-containing ECs, the much better electrocatalytic activity could not be fully accounted for on the basis of conductivity improvement, but to changes in the electronic structure of nickel oxide by inducing partial-charge transfer. Ni_*z*_Fe_z–1_O_*x*_H_*y*_-type ECs show 3 orders of magnitude higher activity in comparison to NiO_*x*_H_*y*_ with reported overpotential as low as 280 mV @ 10 mA cm^–2^. The electrocatalytic performance of iron-incorporated nickel ECs is strongly dependent on the iron content, synthesis method, electrolyte composition, and various catalytic activity parameters can be found, ranging from ∼ 200 mV to more than 300 mV at the current density of 10 mA cm^–2^.^417–419^ Activity of Ni and Co oxyhydroxides increases up to around 25–30% Fe loading, after which a reduction in both activity and durability occurs. The optimal range in 0.1 M KOH was found to be between 24% to 40% Fe atoms.^420,421^ Outside this range, the enhancement of activity in the nickel sites due to the presence of iron atoms is counterbalanced by the decreasing quantity of nickel sites (Figure 25). It was shown that Fe also phase-segregates above 25% as recently shown in ref 422.

**Figure 25:**
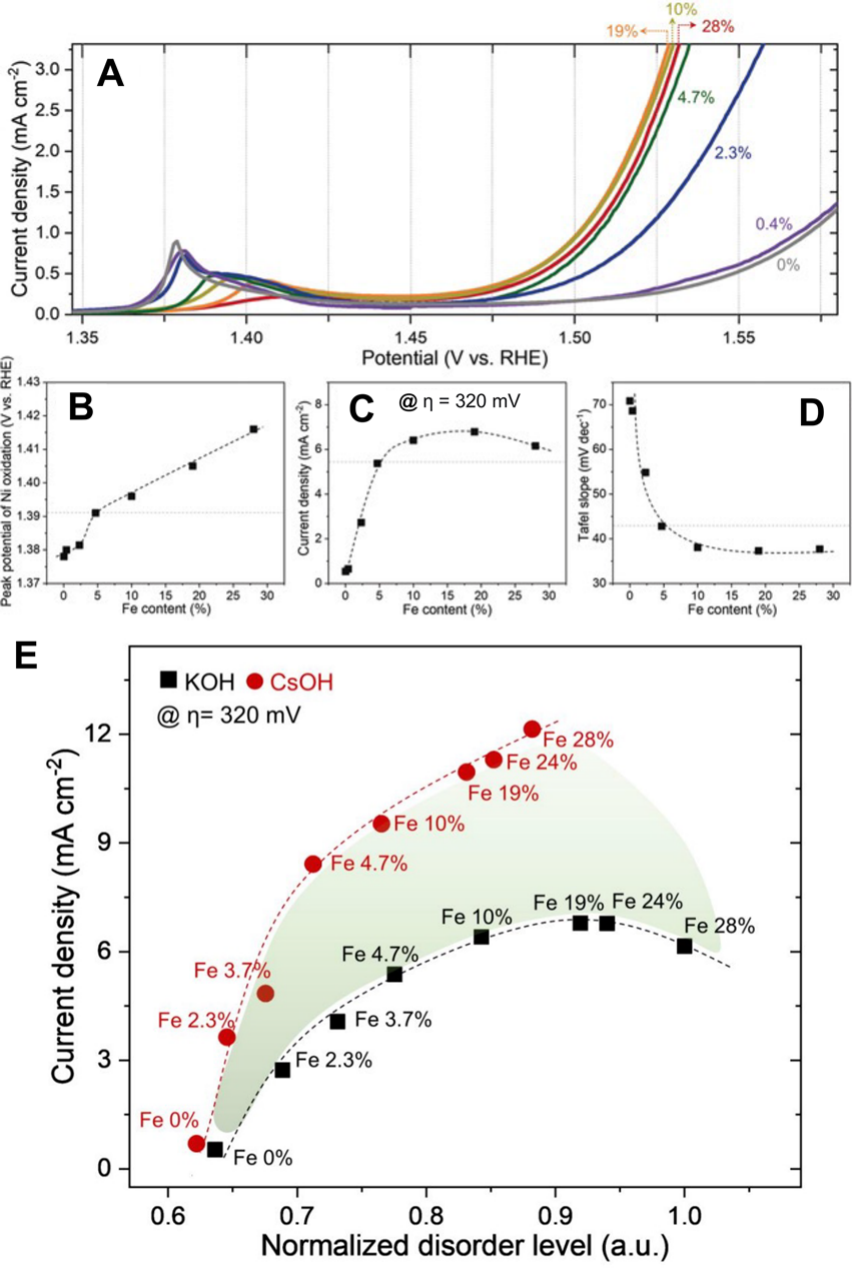
(A) Linear-sweep voltammograms of NiFe LDH samples with various Fe contents recorded at a scan rate of 1 mVs^–1^ in Fe-free 0.1 M KOH. (B–D) Comparison of (B) anodic peak potentials of Ni^2+^ to Ni^3+^, (C) current densities at an overpotential of 320 mV, and (D) Tafel slope. (E) Correlation of current densities acquired at η = 320 mV to Fe content. Adapted with permission from ref 421. Copyright 2020, Wiley-VCH Verlag GmbH.

Iron also plays a crucial role in influencing the stability of nickel oxy­(hydroxide) ECs. Ni_*z*_Fe_z–1_O_*x*_H_*y*_ ECs were reported to have better stability than their Fe-free equivalents. On the other hand, FeO_*x*_H_*y*_ was shown to be less stable than NiO_*x*_H_*y*_.^421^ Since at high loading of iron new phases such as α-FeOOH and possibly γ-FeOOH start to emerge, the stability of Ni_*z*_Fe_z–1_O_*x*_H_*y*_ ECs is expected to decrease at this iron loading range.

Like NiO_*x*_H_*y*_, other transition metal-based LDH ECs show high electrocatalytic activity toward OER. Markovic et al. conducted a study to explore the active sites of various MO_*x*_H_*y*_ ECs (M = Ni, Co, Fe) in Fe-free and Fe-containing electrolytes.^217^ The investigation revealed that in Fe-free 0.1 M KOH, the electrocatalytic activity followed the sequence of FeO_*x*_H_*y*_ > CoO_*x*_H_*y*_ > NiO_*x*_H_*y*_, while the stability exhibited the opposite pattern with FeO_*x*_H_*y*_ < CoO_*x*_H_*y*_ < NiO_*x*_H_*y*_. However, when 0.1 ppm of Fe was introduced, causing Fe deposition on the EC surface, the activity trend reversed. Despite this reversal, the stability of the Fe-containing ECs remained consistent with the non-Fe-containing solution: Fe-FeO_*x*_H_*y*_ < Fe-CoO_*x*_H_*y*_ < Fe-NiO_*x*_H_*y*_.

The influence of iron stands out among other TMs, and numerous studies have aimed to understand its effect by coprecipitating NiO_*x*_H_*y*_ with various metals such as Co, Mn, Ti, La, Cd, Zn, Y, Ce, Cr, Cu, Pb, Ag, and Mg.^423,424^ Within these studies, iron and cerium were observed to significantly reduce the overpotential by more than 140 mV, although Ce-NiO_*x*_H_*y*_ showed faster degradation compared to Fe-NiO_*x*_H_*y*_. Regarding the role of lanthanum, the findings have been contradictory, ranging from no effect to an improvement of 62 mV. These discrepancies can be attributed to variations in testing conditions. On the other hand, the inclusion of Pb, Cd, Zn, and Cr was shown to have a detrimental effect on the EC, leading to higher overpotential compared to the undoped EC. Interestingly, Lyu et al. discovered that the addition of zinc as a third metal alongside iron (NiFeZn LDH) resulted in improved catalytic activity, surpassing that of NiFe LDH.^424^ At 100 mA cm^–2^, NiFeZn LDH shows 30 mV lower overpotential in comparison to NiFe LDH. Similar to NiFeZn LDH, other ternary ECs (*M*_n_M_m_O_*x*_H_*y*_) were investigated, where NiFeCoO_*x*_H_*y*_ is the most studied and promising. Incorporating Co into NiFeO_*x*_H_*y*_ ECs leads to an enhancement in electrical conductivity, which, in turn, results in higher electrocatalytic activity.^425,426^ On the other hand, the presence of cobalt was found to have minimal impact on intrinsic activity.

Overall, up to a certain extent, cobalt and iron impurities were shown to have a positive effect on NiO_*x*_H_*y*_ ECs for OER^427^ while metals such as Cd or Pb can poison the EC, causing a fast decline in performance. The utilization of ultrapure water with AEM-WEs needs to account for the influence of those materials. If KOH is added, its presence cannot be overlooked. Since almost all studies on the effect of impurities on the ECs were conducted in half-cell, the exact role and influence of those impurities under real operating conditions need to be determined. In any case, even when using ultrapure water, some impurities are present due to corrosion of the pipeline or an insufficient purification process.

PGM-free ECs for HER are usually nickel and nickel alloys. Nickel is considered a robust EC for HER, although its activity is lower than that of Pt/C in alkaline solution. For example, in 1 M KOH and 10 mA cm^–2^, Pt/C exhibits around 100 mV less overpotential than Ni/C.^428^ Nickel as a single metal also shows a decline in performance over time, which is attributed to hydride formation.^429,430^ In contrast, alloying of nickel has shown much higher activity and durability, sometimes exceeding that of PGM ECs.^431–433^ Under reductive conditions at the cathode, metal impurities like iron, copper, and zinc can be deposited on the EC. The deposition of a metal layer on the EC leads to the immediate blockage of catalytic sites.^434^ If the deposited metal is less active than the EC, a decline in performance is anticipated. Divisek et al. showed that under A-WE realistic working conditions, iron is deposited on the cathode, causing poisoning of the EC and an increase in the overpotential.^435^ Under highly alkaline conditions, Fe ions are in the form of dihypoferrite and the reaction taking place at the cathode can be written as

$$HFe{O_{2}}^{-}+3H^{+}+2e^{-}⇌Fe+2H_{2}O{,E}^{0}=0.493Vv\mathrm{s.}SHE$$ 19

Conversely, several studies demonstrated that the deposition of iron can enhance the activity and durability of nickel ECs.^436,437^ For instance, Mauer et al. conducted a long-term experiment lasting 45 h at 100 mA cm^–2^ and observed that the uncoated nickel cathode experienced a significant increase of 380 mV in overpotential, whereas the iron-coated cathode displayed minimal signs of deactivation. This behavior was attributed to the prevention of hydride formation facilitated by the presence of iron. This phenomenon was also observed with other transition-metal-based ECs by the formation of binary and ternary alloys of Ni with different elements such as Co, Fe, Mo, Ce, Zn, and Cu.^438,439^ While there is a wealth of literature available on the impact of metallic impurities on ECs for the OER, there is a limited number of systematic studies investigating the relationship between impurities and ECs for HER.^440^ Even more, there are contradictory reports, and no baseline was created to enable a fair comparison. Same as with nickel LDH ECs, it is expected that the effect of iron, for example, on hydride prevention is loading-dependent and above a certain amount, the opposite effect will take place.

Alkaline-earth metal ions impurities can affect both the anode and cathode by the formation of insoluble deposition products on the electrode, blocking the catalytic sites. Calcium and magnesium are particularly problematic under high pH conditions due to their low K_sp_ and high presence in unpurified water. On the other side, it is relatively simple to remove them from the electrolyzer inlet water.^86^

#### 6.3.2 Nonmetallic Impurities

AEM-WE is expected to have a higher tolerance against impurities than PEM-WE, which has PGM-based electrodes that are susceptible to poisoning and to the presence of anions.^86^ In addition, the use of neutral to basic media in AEM-WEs thermodynamically disfavors the oxidation of chloride ions to Cl_2_/HOCl/OCl^–^ species at the anode, which would otherwise lead to the accelerated deterioration of cell components.^441^ Moreover, chlorine and/or bromine evolution is thermodynamically more favorable under acidic conditions and causes the blocking of active sites or the dissolution of noble metal ECs.^442,443^ Similarly, Zhang et al. reviewed the rational design of OER ECs for seawater electrolysis, including: (i) the design of 3D hierarchical porous structures, (ii) the employment of protective layers, and (iii) the engineering of the surface wettability.^444^

The potentials and products of the COR depend upon the Cl^–^ ion concentration and the pH of the solution were previously discussed (Figure 21). In acidic conditions, chloride can be directly oxidized to chlorine gas (COR). Though OER is thermodynamically favored compared to COR, the potential difference between the two reactions is small.^357^ In practice, the COR dominates in acid because it is a two-electron reaction with much faster kinetics. In neutral to alkaline media, COR produces hypochlorite, but at a much higher potential (480 mV) than OER. Thus, alkaline pH suppresses COR if the overpotential at the anode remains below ∼ 1.71 V vs SHE.^357^ In general, seawater oxidation under alkaline conditions is more appropriate to ensure maximum hydrogen/oxygen selectivity.

Like many other metals, nickel and nickel-based ECs are known to exhibit increased corrosion rates in contact with highly saline water where chloride levels are substantial.^445,446^ Several mechanisms are suggested for the corrosion of metal ECs in the presence of chloride ions.^446,447^ One suggested mechanism is metal chloride-hydroxide formation.^448^

20 $$M+Cl^{-}⇌MCl_{ads}+e^{-}$$

21 $$MCl_{ads}+Cl^{-}⇌MC{l_{x}}^{-}$$

22 $$MCl_{ads}+OH^{-}⇌M{\left(OH\right)}_{x}+Cl^{-}$$

Li et al. tested NiCoFe phosphide (NiCo-FeP) nanoarrays grown on the Ni foam in 1 M NaOH solution and 1 M NaOH+NaCl (sat.) for HER and OER.^367^ In the electrolyte containing 1 M NaOH, OER maintained a high Faradaic Efficiency (FE) of approximately 100% throughout the 1h test. This observation indicated that there was no chloride oxidation occurring in the presence of such a high concentration of hydroxide ions. However, when the electrolyte was changed to 1 M NaOH + NaCl (sat.), the FE dropped to 92% within the first hour. Furthermore, with extended electrolysis time, the FE declined even further, ranging from 46% to 65%. The decrease in FE suggested that a notable portion of the current density was being consumed for electrode corrosion, facilitated by the presence of chloride ions. Similar findings were reported for other ECs.^446,449^ Different strategies, such as electronic-structure modification, exposure of more electrocatalytic sites, better EC adhesion, etc., were suggested for mitigating OER ECs corrosion in the chloride-rich environment.^357,450^

Dionigi and co-workers^357^ identified and tested a design criterion of keeping the anode voltage <480 mV above the thermodynamic OER potential during the electrolysis of near-neutral (pH 9.2) borate buffered electrolyte and 0.1 M KOH with 0.5 M NaCl (Figure 26.A). They showed that the OER electrocatalyst stability in the lower pH electrolyte (using borate buffer alone) was generally worse, probably due to the EC degradation caused by the gradual support corrosion and the parallel electrocatalyst dissolution that in turn, reduced the interfacial pH as a consequence of the limited borate buffer capacity.^357^ The introduction of NaCl to the borate buffer enhanced the corrosion issues.^357^ Dresp et al. endeavored to overcome the use of the results from the group’s three-electrode measurements described above to rationalize building an asymmetrically fed electrolyzer as exemplified in Figure 26.B.^451^ This configuration involved feeding seawater to the cathode chamber and 0.5 M KOH to the anode. NiFe-LDH, being an anode EC, demonstrated outstanding OER selective activity without Cl^–^ oxidation, delivering high power efficiencies. As can be seen in Figure 26.C, during the potentiostatic test over 12 h at the cell voltage of 1.7 V the typical symmetric feeding of 0.5 M KOH realized the best performance, whereas the asymmetric configuration with dissimilar feed to anode and cathode demonstrated better performance than the configuration containing mixed feed symmetrically and when the anode or cathode was kept dry.

**Figure 26:**
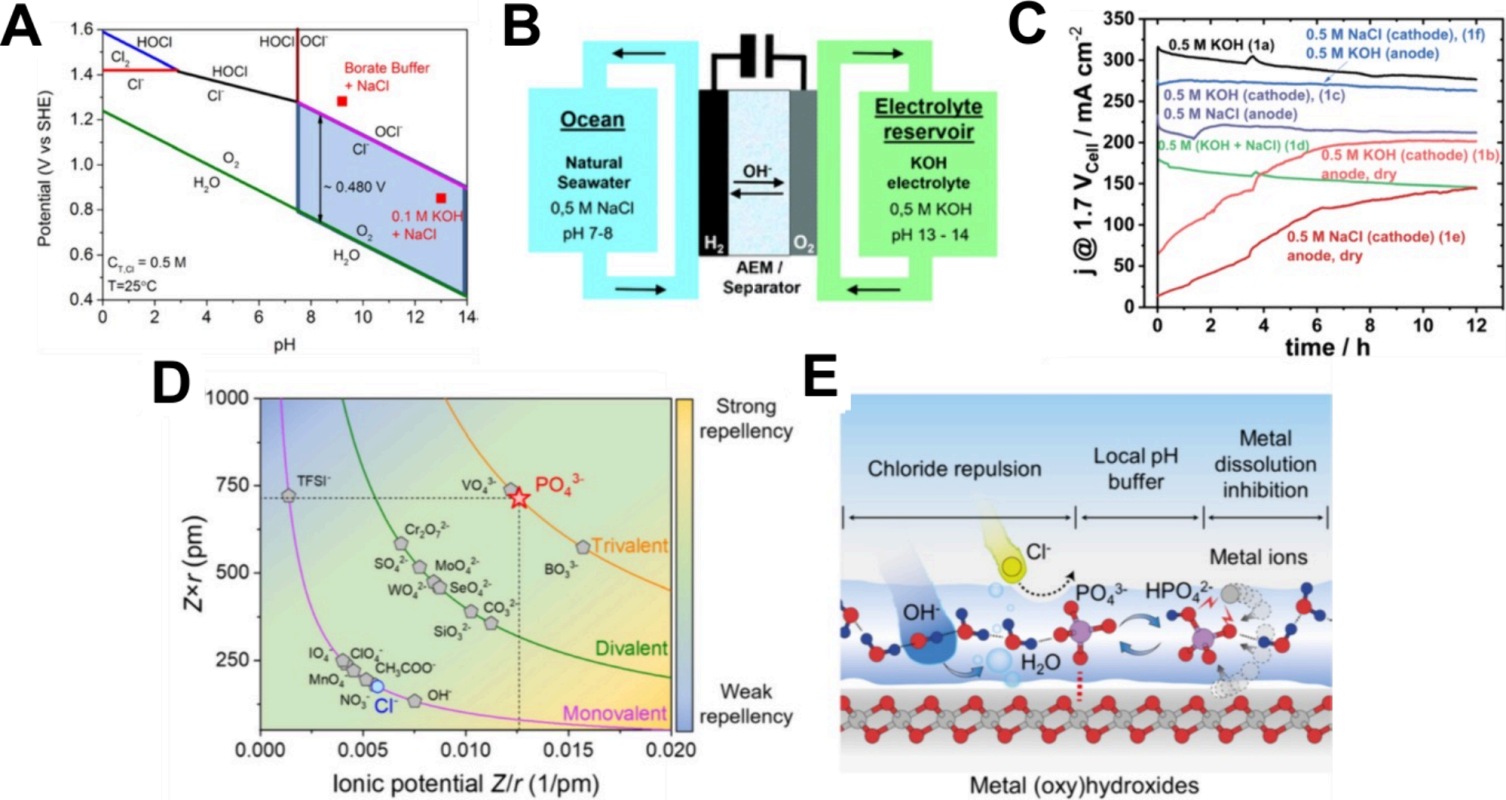
Pourbaix diagram for an artificial model of seawater (A). Reproduced from ref 357. Copyright 2016, Wiley-VCH Verlag GmbH. Schematic illustration of an asymmetric configuration of AEM-WE with seawater feed to the cathode while circulating KOH on the anode (B). Stability trends electrolyzers with different electrolytic feeding conditions at a fixed cell potential of 1.7 V over 12 h (C). (B–C) Reproduced from ref 451. Copyright 2020, The Royal Society of Chemistry. Licensed under CC-BY 3.0. (D) Volcano trend of repulsion between different anions and chloride ions as a function of ionic potential and charge number. (E) Schematic illustration of corrosion-avoiding mechanism due to surface adsorption. (D-E) reproduced with permission from ref 452. Copyright 2022, Elsevier B.V.

One way of mitigating the degradation of anode EC by the products of chloride impurity oxidation is to screen the electrode using anionic additives,^452^ which have been shown, in some cases, to adsorb to metal oxyhydroxides.^453^ Recently, Yu and co-workers established an anion-induced performance-enhancing approach through an inclusive screening of electrolytic additives by relating the chloride repulsion with anionic properties.^452^ The volcano curve presented in Figure 26.D implies the repulsion between chloride and other typical anions as a function of ionic potential and the multiplication product of charge number (z) and radius (r). Anions having a greater ionic potential and *Z* × *r* factor tend to electrostatically repel the Cl^–^ ions, whereas *PO*_4_^3–^ demonstrate optimum electrochemical durability along with a robust interaction with water molecules via hydrogen-bonding, approving the adsorption of *PO*_4_^3–^ to build a fragile “semi-permeable film” with superficial water molecules that deter the flow of Cl^–^ ions without inhibiting the diffusion of OH^–,^ as schematically illustrated in Figure 26. E. In the interim, the transition between *PO*_4_^3–^ and *HPO*_4_^2–^ may create a local buffering to recompensate the pH drops at higher current densities and therefore enhance the performance and stability of the electrode.

Kuang et al. synthesized a multilayer anode consisting of a nickel–iron hydroxide (NiFe) EC layer uniformly coated on a nickel sulfide (NiS_*x*_) layer formed on porous Ni foam (NiFe/NiS_*x*_/Ni), and tested its stability in 1 M KOH+real seawater (25 °C), 1 M KOH + 1.5 M NaCl (25 °C), and 6 M KOH electrolyte (80 °C).^364^ Negligible performance decline was observed at 400 mA cm^–2^ after 1000 h of operation time. What is common to all ECs that are chloride-corrosion resistant is the requirement of high pH levels (>14). Under lower pH, those ECs show significant corrosion and fast performance decline. Despite these ongoing efforts, further research is needed to fully understand the mechanisms of chloride-induced corrosion and develop robust mitigation strategies. The optimization of electrolyte compositions, the development of corrosion-resistant materials, and the implementation of effective monitoring and maintenance practices will be crucial to minimize anode corrosion and enhance the durability and performance of AEM-WEs in the presence of chloride ions.

While extensive research has been dedicated to studying the corrosion-enhancing effect of chloride ions, there is a relative scarcity of literature discussing the impact of other commonly occurring anions, such as bromide (Br^–^), sulfate (SO_4_^2–^), bicarbonate (HCO_3_^–^), and carbonate (CO_3_^2–^), on their role in facilitating or inhibiting corrosion. Such studies would contribute to a more comprehensive understanding of corrosion mechanisms and aid in the development of effective corrosion control strategies in various aqueous environments. Ma et al. studied nickel foam (NF) and Fe-LDH/NF as ECs for OER in 1 M NaOH with various concentrations of NaCl and sulfate to elucidate the role of the latter as an additive for corrosion inhibition (Figure 27).^454^ They demonstrated that the presence of sulfate ions in the electrolyte effectively mitigates chloride-induced corrosion on the anode in alkaline seawater electrolysis, leading to significantly improved stability of the anode during operation. This phenomenon was attributed to the selective adsorption of sulfate ions on the surface of the anode, which repelled chloride ions present in the bulk solution through electrostatic repulsion. Thanks to the repulsive effect of sulfate ions, the active NiFe-LDH nanoarrays/Ni foam anode exhibited stable performance for 500–1000 h in both simulated and real seawater environments. Once again, this highlights the significance of comprehending the role of all the species present in seawater to facilitate the precise development of active and durable ECs for seawater electrolysis.

**Figure 27:**
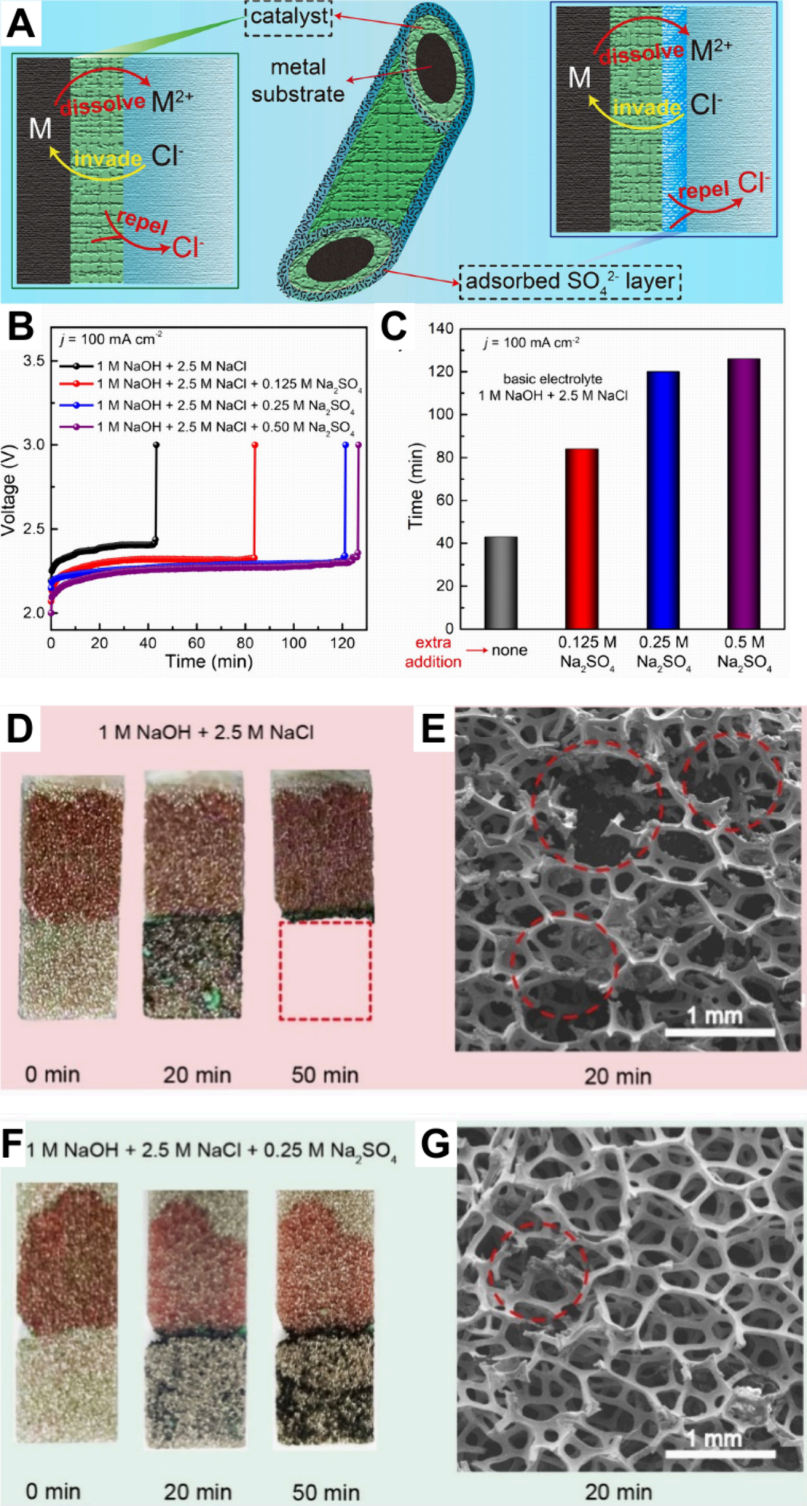
Left- (A) ECs optimization (left) and electrolyte optimization (right) to protect the metal substrate from Cl^–^ corrosion; (B) stability tests recorded at a constant current of 100 mAcm^–2^ for pure NF in electrolytes with different proportions of Na_2_SO_4_; (C) the durability of NF in different electrolytes. (D) and (F) optical images and (E) and (G) SEM images of NF working in two different electrolytes after 0, 20, and 50 min, the dashed circles indicate the skeleton collapse by electrolyte corrosion. SEM images of the skeletons of untreated NF. Adapted with permission from ref 454. Copyright 2021, Wiley-VCH GmbH.

## 7 Cell and Stack Degradation

The long-term stability of electrolyzer cells depends on the durability of each individual component, which in turn is a function of the operating conditions. To achieve high energy efficiency and improved operation capability, the polymer electrolyte membranes must inhibit gas crossover and sustain high currents with minimal ohmic losses.^274^ In AEM-WEs, H_2_ diffusion coefficients through the membrane are lower than in PEM ones, but can practically be more challenging to handle due to the degradation and the mechanical thinning of the ion-exchange membrane.^282^ The morphology of the EC layer in an AEM-WE system has a significant influence on its performance, including electrochemical activity, mass transport, stability and durability, water management, and EC utilization.^54^

Flow-field patterns, bubble evolution, and transport affect the overall performance and durability of AEM-WE, but due to the relative newness of the technology, it remains an understudied area of research that previously was touched upon in discussions of PEM electrolysis.^455^ It is important to look at the AEM-WE system as a whole, considering interactions between components. As stated, there are two primary feed conditions used in stability tests for AEM-WEs: electrolyte feed (typically 0.1 to 1.0 M KOH) and pure-water feed. In the case of electrolytes, additional hydroxide ions are introduced to the system, exposing its components to higher alkaline concentrations, possibly accelerating caustic degradation of system components in addition to the membrane and ionomer from the known chemical mechanisms.^456^ Moreover, ionomer failure caused by the alkaline oxidative conditions in the EC layer at the anode could be concealed by the hydroxide- or carbonate-based electrolytes. When it comes to the outer level of the cell, the attention of researchers is focused on the development of cheaper and more resistant alternatives to BPs commonly used in hydroxide-rich devices. Using carbon-coated stainless-steel BPs proved to be an efficient approach to slow down the anodic film growth in AEM-FC that causes an increased contact resistance and subsequently ohmic losses during the operation.^330^ However, this topic remains underdeveloped for AEM-WE in comparison to PEM-based devices, where variation of stainless-steel coatings is frequently explored to achieve a better long-term performance of the device.^457^ With a pure-water feed supply, which is the ideal operational scenario for manufacturing and scaling if it is efficient and durable, hydroxide ions are only generated at the cathode and transported via AEM without supplementary anions. In this section, we focus on the pure-water operation of the AEM-WE, discussing limitations in the durability and stability of these systems, including the analysis of future research directions aimed at improving the above-named characteristics.

Initial research on AEM-WE devices in electrolyte-free (nominally pure) water as a feed electrolyte reported limited stability and relatively poor operational performance. The first implementation of AEM in pure-water electrolysis operation was reported by Xiao et al., recording 8 h of operation with 400 mA cm^–2^ at 1.8–1.85 V at 70 °C with Ni–Fe anode and Ni–Mo cathode.^458^ Within the same time frame, the next report of the AEM-WE operation without an additional alkaline electrolyte by Leng et al. had a value of 399 mA cm^–2^ at 1.80 V at 50 °C using IrO_*x*_ as the anode EC and Pt black as the cathode EC.^459^ Analyzing HFR over time as an indicator for increased membrane resistance contribution and possible delamination in the system, a minimal decay over the 200 h of performance, varying the water feed configuration and the ionomer binder in the process of the cell optimization was reported. The authors suggest using a more durable ionomer, as well as the configurational optimization of the water-fed cell to improve the stability of the system. These findings provided the basic strategy incorporated by different research groups around the world to increase the overall durability of AEM electrolyzers with a pure water feed. Despite initial promising results, according to a 2020 review by Miller et al., initial reports on the pure water operations generally showed an average of 95 mA cm^–2^ at 1.8 V and limited durability of the cell.^19^ Early publications on pure water electrolysis lacked a tailored approach, often mimicking alkaline electrolyte-fed systems with unstandardized electrodes and polymers, leading to poorly comparable data.^282^ Efforts toward standardization were made,^175,460^ including publications with commercially available materials (Figure 28.A).^56^

**Figure 28:**
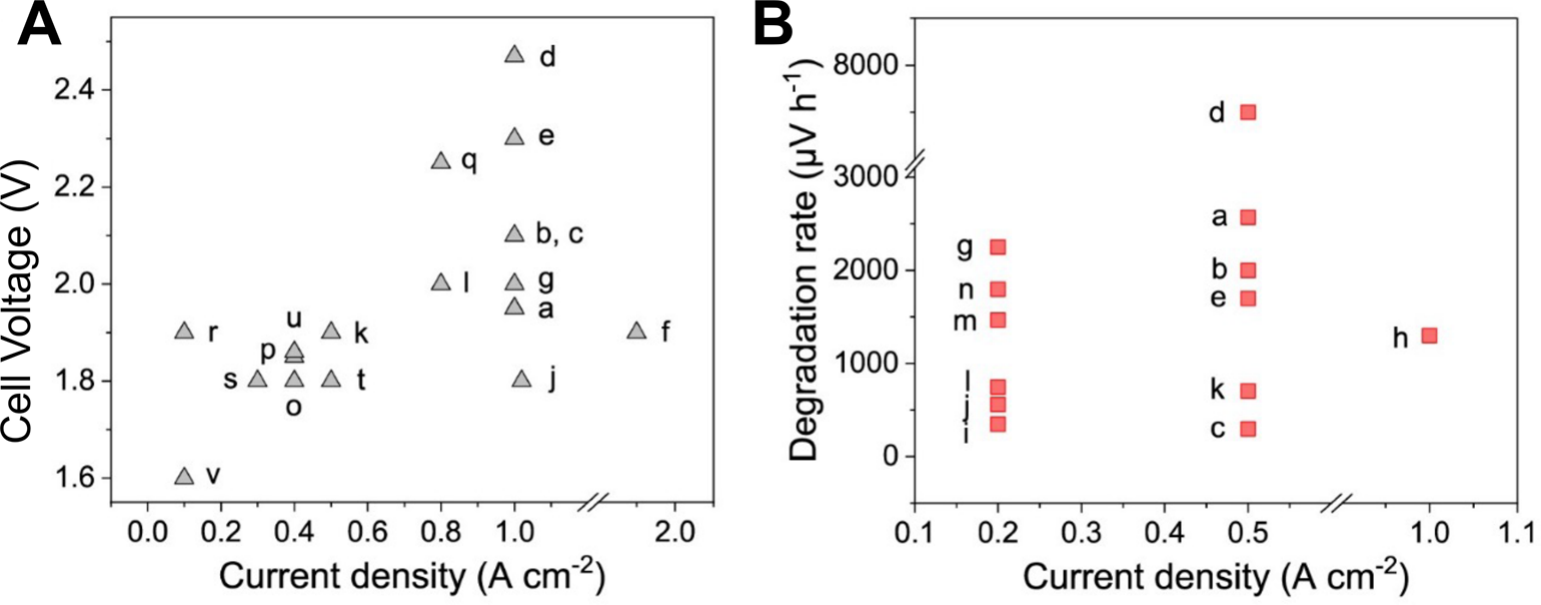
(A) Maximum performance of pure water-fed AEM-WEs. References and operational temperatures are labeled on the plot and shown in Table 6. (B) Degradation rate during long-term testing of pure water fed AEM-WE. References and operational temperatures are labeled on the plot and shown in Table 6.

Recent work demonstrated a more competitive performance of pure water-fed AEM-WE devices reaching 1 A cm^–2^ at potentials below 2 V, which is twice the current density of a modern industrial alkaline water electrolysis standard at the same voltage.^461^ Recently achieved maximum performances are 2-fold higher than results from just five years ago. High ionic concentration quaternized polystyrene ionomers with various IECs were used by Li et al. to support the key need for a local high pH environment in the EC binder for efficient HER and OER.^282^ By removing phenyl groups from the polymer backbone, the acidic formation of phenol is prevented, ensuring the maintenance of a local alkaline pH. The system operated in pure water reached a value of 2.7 A cm^–2^ at 1.8 V. However, as was discussed in later publications,^282^ the authors were not successful in removing all the remaining alkali from the system, which affected the reported performance and durability of the electrolyzer. At the same time, the durability of the single-cell in this work remained an issue and at 60 °C and 200 mA cm^–2,^ only 170 h of low-rate decay performance could be observed. These studies often use individually tailored polymer membranes and ionomer binders, as well as specialized GDLs, for example, implementing fluoride-incorporated nickel–iron oxyhydroxide nanosheet arrays on a compressed Ni foam to achieve a competitive performance and reach over 160 h of a continuous operation at 200 mA cm^–2^ with a 0.56 mV h^–1^ decay rate at 70 °C. The durability of this cell at an increased current density of 500 mA cm^–2^ had a higher potential and 1.81 mV h^–1^ decay rate over 70 h.^315^ While it is one of the driving forces for innovation, customization of the AEM electrolyzer components to achieve a specific performance of a single cell affects measured performance metrics and complicates the durability evaluation. Standardization of evaluation criteria is needed for a better understanding of achieved performance and durability (Figure 28.B).

**Table 6:** Summary of the Data Plotted in Figure 28

		Performance		**Durability**			
**Label**	T (°C)	**Cell Voltage**(V)	**Max current density** **(A cm^–2^)**	**Current density** **(A cm^–2^)**	**Degradation rate** **(μVh^–1^)**	**Time**(h)	**ref.**
a	69	1.95	1	0.5	2571	175	^44^
b	55	2.1	1	0.5	2000	250	^427^
c	55	2.1	1	0.5	300	100	^462^
d	50	2.47	1	0.5	7500	20	^249^
e	50	2.3	1	0.5	1700	20	^463^
f	60	1.9	1.9	-	-	-	^316^
g	80	1.95	1	0.2	2250	120	^57^
h	60	2	1	1	1300	85	^398^
i	50		-	0.2	350	2000	^464^
j	80	1.8	1.02	0.2	560	150	^315^
k	60	1.9	0.5	0.5	705	170	^336^
l	50	2	0.8	0.2	747	535	^459^
m	-	-	-	0.2	1470	170	^181^
n	25	-	-	0.2	1800	100	^465^
o	50	1.8	0.4	-	-	-	^396^
p	70	1.85	0.4	-	-	-	^458^
q	50	2.25	0.8	-	-	-	^249^
r	50	1.9	0.1	-	-	-	^459^
s	25	1.8	0.3	-	-	-	^402^
t	50	1.8	0.5	-	-	-	^466^
u	60	1.86	0.4	-	-	-	^467^
v	22	1.6	0.1	-	-	-	^468^

Stability and degradation testing in pure water allows for researching the fundamental properties of AEM-WE components and target system development toward the ideal electrolyte-free operation. However, in this condition, the impact of the water additives can be detrimental to the system; hence, the study on the sensitivity to the water quality and specific impurity contaminants of the AEM-WE would be useful. This part has already been treated above.

Recently, stack-level measurements on AEM-WE translated performance to a commercial magnitude. Park et al. demonstrated a 5-cell stack system that operated for 150 h at 440 mA cm^–2^ per cell (28 A total current).^469^ This system, fed by 1 M KOH, degraded at a rate of 2 mV_stack_ h^–1^, bringing the initial efficiency of the cell 69% to the final 59% at the end of the stability test. To be considered a commercial-level AEM-WE stack, the device must display a degradation rate below 1 mV 1000h^–1^ and maintain over 90% of the initial performance for 20 years of use.^470^ Performance and durability of the stack were influenced by the electrolyte behavior, in particular, the effect of turbulent and laminar flow was discussed. As the number of stack components increases, the behavior of the electrolyte becomes significantly different than that in a single cell. A fluid-mechanical analysis is required for a proper stack design evaluation to consider all the parameters influencing the final performance of the device. Although the stack structure itself, as well as electrolyte feed flow, poses certain limitations to the performance to be achieved, this research opens a door to the future with large-scale stack-level explorations.

### 7.1 Effect of Ionomer

As stated above, there are chiefly two types of AEI chemistry: (i) polymer containing side chains attached to the backbone of the polymer bearing fixed positively charged groups, and (ii) ionenes, charged polymers with cationic groups attached along the backbone of the polymer. The latter is considered an example of an ionomer with enhanced durability due to the advanced structure, which eliminates some of the alkaline degradation mechanisms.^271,275^

At the cell level, AEIs historically limited the performance and durability of AEM electrolyzer devices, especially in pure water.^288,289,460,471,472^ Common cation exchange ionomers are PFSA-type materials with high chemical stability.^282^ Most anion exchange ionomers are hydrocarbon-based (e.g., polybenzimidazoles, polyethers, polyphenylenes, etc.), which are more susceptible to chemical degradation by OH^–^ in strongly alkaline conditions. Efforts at improving the stability of AEM polymers have focused on alkaline chemical stability, including adding protecting groups near electrophilic sites,^404,473^ partial fluorination^474^ and tuning side chain length^475^ or cation identity.^476,477^ *Ex-situ* chemical stability, however, is not necessarily reflective of device conditions nor indicative of how a polymer will perform in an MEA, in particular during operation with electrolyte-free water feed. Multiple chemical and electrochemical degradation pathways were identified for a variety of alkaline ionomers in pure water. Alkaline ionomers are susceptible to degradation via nucleophilic substitution, elimination, phenyl oxidation, and methyl and/or proton-rearrangement reactions, depending on polymer structure.^266,364,475,478,479^ Water-transport limitations during device operation may enhance degradation due to locally increased OH^–^ concentration.^480–482^ These pathways break down the membrane structure, leading to a reduced ionic conductivity due to the loss of charged end groups, either directly by degrading cations or indirectly through backbone or side chain degradation, resulting in soluble ionomer fragments.

At the anode, the ionomer is held at a strongly oxidizing potential and exposed to possible oxygen radical species produced during OER. The study that proves the presence of stable radicals, records and identifies all the short-lived radical species under alkaline conditions was first conducted for the AEM-FC application.^483^ Superoxide and hydroxyl radicals could be spontaneously created within the polymer electrolyte material in the presence of oxygen and hydroxide ions. This work suggests the formation of stable radicals as a result of the short-lived oxygen radicals on the membrane backbone.^483,484^

Organic materials are disreputably difficult to stabilize under alkaline OER conditions, as has been shown for carbon blacks.^96,485^ It was proven that the presence of a highly active OER EC prevents carbon from oxidation in alkaline conditions due to the enhanced reaction kinetics, which reveals a closer look into carbon support behavior during this electrochemical reaction. This statement agrees with observed carbon corrosion in the presence of Pt EC, known for its sluggish kinetics in the hydroxide-rich environment.

Fenton oxidation processes (4 ppm FeSO_4_ in 3% H_2_O_2_), forming radical oxygen species, can also degrade the ionomer and membrane.^486^ Adopted from PEM-FC technology research, acidic Fenton processes could be a source of degradation in AEM systems with active nonprecious-metal OER ECs, which all contain Fe but have not been studied extensively in this context due to the high alkalinity of the system, tempering with a Fenton test chemistry. The oxidative and radical stability of anion exchange polymers was investigated to various extents,^479,487,488^ but few studies have been conducted under device-relevant operating configurations and conditions. Future research on oxidative stability might be performed under relevant alkaline conditions, for example, using alkaline peroxide solutions like those used in the RCA semiconductor cleaning procedures for organics,^489^ with a standard metal oxide EC nanopowder dispersion, for example, using commercial CoO_*x*_.^490^

The ionomer binder in contact with the EC layer oxidizes under operating potentials at the anode.^56,485^ The mechanism by which this oxidation occurs is poorly understood.^56,254,282^ Oxidation could occur directly at the EC/ionomer surface or chemically through reactivity with OER intermediates or other reactive oxygen species formed, for example, through radical reactivity.^491,492^ AEM ionomers in a pure water operation will oxidize at sufficiently high voltage regardless of EC/electrode surface,^254^ thus degradation may only depend on the device operating voltage and feed. Others have suggested that degradation rates correlate with ionomer/EC interaction strength,^493^ and the adsorption energies of ionomer functional groups to the EC surface are expected to vary for different oxide ECs.

One key research direction is to distinguish between degradation mechanisms of the bulk membrane, membrane interfaces, ionomer binders, and the ECs themselves in the presence of pure water, alkaline-electrolyte, and impure-water feeds. Li et al. probed the performance difference observed when varying the chemical structure of the binder polymer using the same membrane.^181^ They showed that the voltage performance and, in the long term, durability depended on the ionomer structure and loading. This was attributed to interfacial pH control. Phenyl groups in the polymer backbone can oxidize to form phenol, which was hypothesized to decrease surface pH and neutralize hydroxide associated with the cationic ionomer groups.^255^ Fully sp^3^-carbon polymers showed more stable performance^398,465^ and resistance to oxidation in *ex-situ* Fenton testing and during fuel cell operation,^493^ but still suffer from oxidative damage at the high operating voltage of AEM-WE (Figure 29).^462,494^

**Figure 29:**
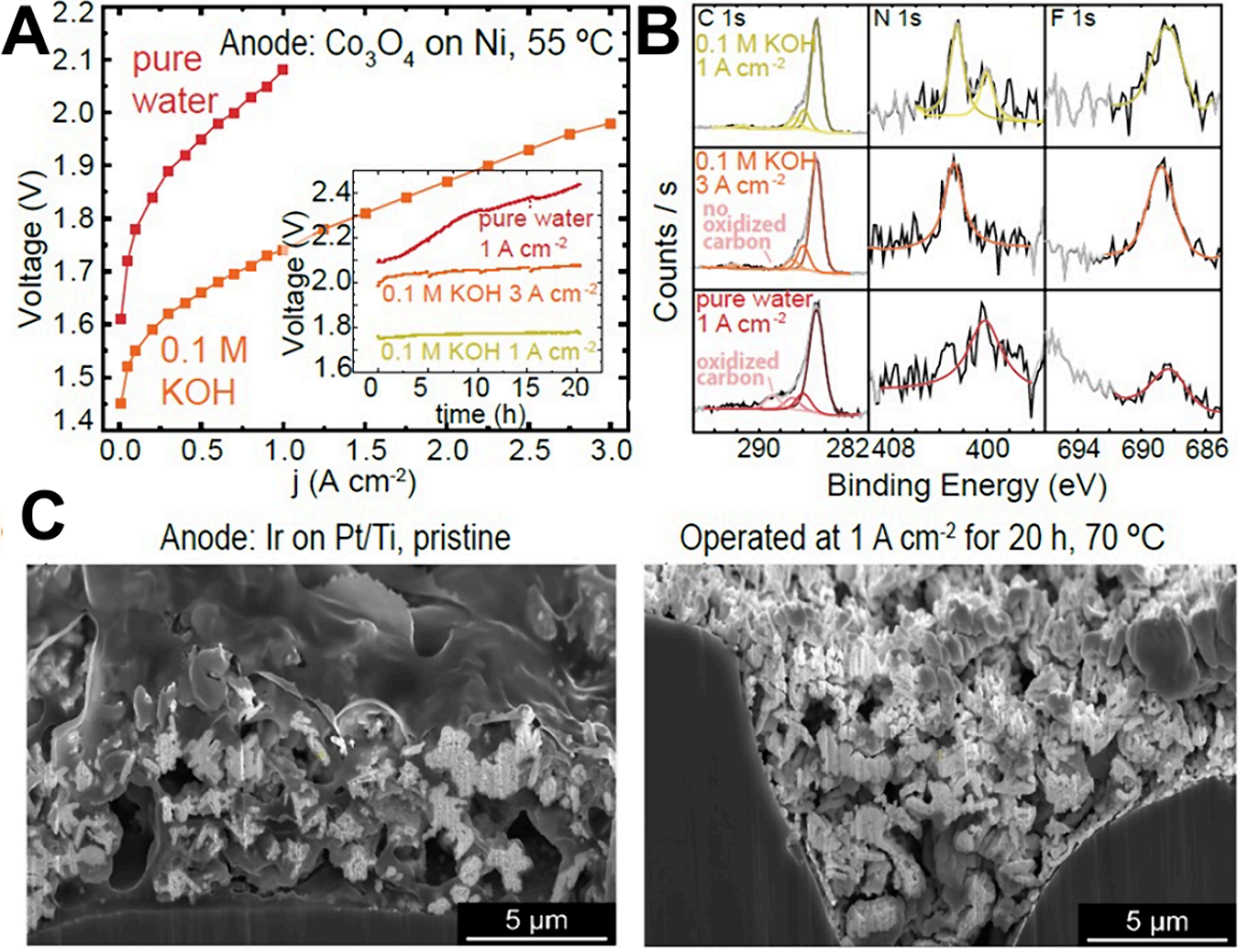
(A) AEM-WE operation in different feed conditions demonstrates ionomer oxidation suppression in the presence of 0.1 M KOH. (B) XPS data show the presence of oxidized carbon after AEM-WE pure water operation, indicating ionomer oxidation. (C) Cross-sectional images of an IrOx/TP-85 anode PTE before and after operation show oxidative degradation of ionomer, causing its loss. Reproduced with permission from ref 462. Copyright 2023, The Royal Chemical Society

Mustain and co-workers showed how the water uptake by the AEI can affect device stability, and found that low water uptake at the anode improves stability despite the performance losses from low ionic conductivity.^288,398^ This hypothesis was supported by Chen et al. where a reduction in ionomer swelling via a controlled benzylation (cross-linking) of polybenzimidazole-type polymer resulted in four times prolonged operation of a pure-water operated AEM-WE system. Additionally, the above-mentioned work reports that the impact of the ionomer swelling within an EC layer is more detrimental to the stability of the device than the dimensional stability of a polymer electrolyte membrane incorporated in it.^495^ Water uptake will also affect how chemical OH^–^ and radical oxygen species access and interact with the polymer, and thus, polymers with lower water uptake may not degrade as rapidly. Expansion of the polymer chain network, followed by the water penetrating the bulk of the material, allows better distribution of the degradation-inducing species. At the same time, narrow channels of a less swollen ionomer could reduce the amount of hydroxides and radicals entering the polymer. PTFE-containing (polytetrafluoroethylene) anodes are resistant to oxidative damage. This type of fluorinated additive is a possible route for increasing the AEM-WE pure water operation, but not a viable commercialization pathway alone due to the high ionic transport resistance leading to low voltage efficiencies.^462^ While PTFE on its own shows poor voltage performance, Alford and co-workers reported a performance of ∼ 700 mA cm^–2^ at 2 V using a PTFE-based hydroxide conductive membrane that degraded at a rate below 40 μV h^–1^ over 120 h.^468^

The problem of ionomer oxidation is much more prominent for pure-water operation. Using X-ray photoelectron spectroscopy (XPS) analysis, Lindquist et al. showed stable cell operation in 0.1 M KOH at 3 A cm^–2^ with no substantial evidence of oxidative damage after 20 h.^462^ However, it is worth noting the limitations of this method, such as surficial analysis without consideration of surface roughness/uniformity, as well as ionomer presence on the spectrum have to be analyzed carefully since low polymer loading will result in a lack of signal-to-noise ratio. Additionally, signals from carbonate/bicarbonate could interfere by appearing in the binding-energy range where oxidized carbon species are found, so all samples must be carefully exchanged to chloride form prior to the evaluation. An identical cell operating in pure water at 1 A cm^–2^ (the equivalent starting voltage of the KOH cell) degraded rapidly. This difference was hypothesized to be due to changes in the local double-layer structure. Under alkaline conditions, metal-oxide surfaces are likely negatively charged due to deprotonation, which promotes the adsorption of cationic species or polymer backbone groups from the ionomer—a phenomenon previously suggested for other reasons.^291,476,496–500^ Without supporting electrolytes, ionomer, therefore, must play a fundamental role in the formation of the double layer, requiring it to be in close vicinity to the polarized EC. The presence of mobile, soluble ions in the supporting electrolyte likely leads to displacement of the ionomer from direct involvement in the double layer, and this may be responsible for substantially reducing the degradation rate compared to pure water. It was shown that the structure of the double layer at the anode EC is critical in controlling the rate of deleterious ionomer oxidation during OER.^462^ IrO_2_ and Co_3_O_4_, the best anode ECs in pure-water AEM-WEs,^427^ are acidic or neutral oxides with a pH of zero charge of <7. In base, they thus have a negative surface charge. In the KOH electrolyte, mobile K^+^ will compensate for this charge such that the ionomer resides outside of the double layer where deleterious oxidation occurs. In electrolyte-free devices, the ionomer is absorbed electrostatically on the OER EC and thus susceptible to deleterious oxidation. This work demonstrates that controlling the reactive EC/ionomer interface enables one to slow the oxidation, e.g., by surface-charge modification with absorbed Mg^2+^ or Ca^2+^. Alternative strategies to exclude ionomers from the double-layer region might also be expected to reduce oxidation rates.

### 7.2 Ionomer Poisoning

One of the major factors that limit the durability of AEM-WEs operated with pure water is the electrochemical oxidation of the adsorbed phenyl group present in the ionomer, which occurs at the potentials required for oxygen evolution. One of the possible approaches for mitigating the impacts of phenyl group electrochemical oxidation is to use polymers with a lower affinity for these groups. For example, quaternized polyolefins, which have lower adsorption energy than quaternized polyaromatics, could be a viable option. Additionally, using nonrotatable phenyl groups like fluorene or carbazole, which have lower adsorption energy than rotatable phenyl groups like biphenyl, could help to reduce the impact of this degradation mechanism.^57,282,472,473^

Water transport occurs from the anode to the cathode side, especially in a “dry cathode” configuration. Design AEMs with higher water diffusivity,^404^ using cathode ionomers with high IEC, and increasing the operating temperature are approaches that would help in providing more reactants to the cathode side and prevent the chemical degradation of the ionomeric materials.

Phenyl oxidation of the ionomer occurs during the operation of AEM-WE. Minimizing the adsorption of the phenyl group on oxygen evolution ECs is achievable by employing PGM-free perovskite oxides. In addition, developing new ionomers with fewer phenyl-adsorbing traits could be a potential direction for making durable AEM electrolyzers.

The development of AEMs with high hydroxide conductivity and IEC is critical while ensuring robust mechanical and chemical stability. Ionomer with high IEC and water uptake, especially at the cathode electrode, to prevent the dehydration of the ionomer.

### 7.3 Effect of Electrocatalyst

In alkaline electrolytes, Ni–Fe oxyhydroxides have the lowest overpotential for OER and the highest per-cation turnover frequency in three-electrode studies.^223,411,425^ However, most AEM-WE testing is conducted with expensive IrO_2_ OER EC,^56,57,249^ as the performance and durability of Ni–Fe oxyhydroxides have generally been poor in pure-water MEA configurations. Indeed, PGM-free ECs have shown promising performance and durability in MEA configurations, mainly with supporting electrolyte^212,501,502^ and or complex electrode and/or EC design and preparation,^181,315,460,503–505^ to overcome ionic and electronic conductivity issues. We expect that these observations are related to the complicated *in situ* behavior of PGM-free ECs.

For one, PGM-free transition metal oxide OER ECs are known to structurally evolve under operating conditions.^411,425^ Initially, crystalline NiO amorphized during the OER.^216^ *Operando* surface X-ray diffraction showed a reversible formation of a CoOOH “skin layer” at OER potentials on Co_3_O_4._ ^506^ Perovskite oxides have been reported to show a dazzling variety of dynamic behavior.^507^ Direct evidence of CoOOH formation via leaching of surface-segregated Sr-rich areas has been reported for La_0.2_Sr_0.8_CoO_3._ ^508^ Such A-site leaching has also been shown to be concurrent with surface oxygen vacancy formation in related materials.^412,509,510^ LaNiO_3_ exchanges surface oxygen with oxygen in the electrolyte^511^ and displays NiOOH-like redox behavior when doped with strontium.^512^

Restructuring could be especially detrimental in the pure-water-fed mode. Significant reconstruction may disrupt the interface between EC and ionomer/membrane, which is the only means of ionic transport to the anode. Importantly, these ionic conductivity losses should be recoverable upon the introduction of supporting electrolytes, consistent with the literature. It may even be the case that EC detachment from the ionomer in the EC layer induces oxidative degradation because the current demand cannot be effectively met by driving OER alone, but also by oxidizing the ionomer. Further, restructuring may form an insulating material such as Ni­(OH)_2,_ which must be made conductive by driving the Ni^2+^ to Ni^3+^ reaction in the presence of hydroxide.

In general, investigation of the extent and effect of these EC structural transformations in relevant MEA configurations is in its infancy^209,401,513^ and complicated by membrane and ionomer instability that causes degradation independent of EC identity. Lei and co-workers reported a decrease in MEA performance for a system with amorphous NiCoFe oxide particles only under pure water-fed mode, thus concluding that the decrease was driven by EC reconstruction.^514^ Krivina et al. tested various PGM-free OER ECs in traditional EC-coated-electrode MEA configurations in pure water. NiFe_2_O_4_ nanoparticles failed rapidly during electrolyzer testing, but Co_3_O_4_ nanopowders showed comparable performance and stability to high-surface-area commercial IrO_*x*_.^427^ The enhanced performance was attributed to differences in EC electronic conductivity and changes to ionomer EC interactions during operation. Co_3_O_4_ was found to have the highest electronic conductivity of all PGM-free ECs tested and showed resistance to structural rearrangement during operation. All Ni-based and mixed metal ECs showed substantial surface rearrangement during operation, likely disrupting the EC/ionomer interface. Notably, others have shown Ni–Fe oxide ECs perform well in AEM devices when fed with KOH^404,515^ or in pure water when using a thin layer of NiFe EC on a conductive supporting substrate, likely to compensate for the poor electronic conductivity of the material when not fully converted to the oxyhydroxide form.^315,516^

It has also been shown in liquid alkaline electrolytes that PGM-free OER ECs have only high activity when Fe is present.^425,517,518^ Fe catalytic sites are not static, but dynamic. They can form by adsorption from electrolyte^223^ and actively dissolve and redeposit during OER.^217,519^ If there is no soluble Fe available for redeposition, the EC becomes much less active.^520^ Maximizing Fe adsorption is not necessarily a universal strategy for high activity; the performance of an AEM with a NiFe_2_O_4_ particle-based anode improved as Fe leached out.^521^ These ECs may also uptake additional ions present in trace amounts in the electrolyte.^522^ Thus, for Ni and Co-based ECs, management of ion adsorption/desorption processes, especially of Fe, is another understudied challenge that must be translated from three-electrode to the MEA levels.

Observed differences in ion dynamics between a three-electrode and MEA system will likely arise from the presence of the ion-selective membrane and directed electrolyte flow in the latter. The flux of OH^–^ from cathode to anode, combined with the membrane permselectivity, drives anions to the anode. This may lead to regions of higher pH. If this led to deactivation, the addition of Fe into the electrolyte stream could be attempted to recover activity. Maximizing activity must be undertaken simultaneously with preventing the movement of dissolved metals and poisoning of the cathode. The AEM configuration may be an interesting platform because the flow of cationic impurities (e.g., M^+^ ions leached from the OER EC) from the anode to the cathode should be retarded by the membrane permselectivity (i.e., the selective conduction of anions over cations). The flow of OH^–^ from cathode to anode would also reduce the extent of anion exchange and crossover of anionic metal oxo anionic species. Post-mortem analysis, such as measuring elemental composition and concentrations of leached species in the outgoing electrolyte during the operation of MEAs, would be useful in understanding element speciation during operation and its relationship to durability.

### 7.4 Advanced Active Layer Design

While substantial gains have been made in advancing alkaline ionomer stability,^56,57,315^ polymer oxidation at the anode is proving a substantial barrier that may not be solved by polymer design alone. Advanced EC layer designs that incorporate stable additives are an interesting new direction that may enable stable pure water operation. The most stable electrodes appear to be prepared with ground ionomer resin particles as opposed to the conventional dissolved/dispersed ionomer solution in the ink (Figure 30.A). The observation was first made by the Varcoe group in 2014 for the AEM fuel cell technology, where the addition of insoluble ETFE-based (ethylene tetrafluoroethylene) polymer particles to the EC layers of the systems instead of the conventionally dispersed ionomer binder improves the performance of the cell.^523^ Continuing this research thread, in 2018, Mustain and team showed how the addition of fluorinated insoluble polymer resin improves the water management of the AEM-based fuel cell system, which is considered one of the technology limitations.^524^ In order to prepare inks with incorporated insoluble ion-exchange polymer powders, an anion exchange ionomer with a particle size of 20–30 μm and an EC were ground with a mortar and pestle, followed by the addition of a solvent until a low-viscosity mixture was achieved. Deposited by ink spraying, the PTL EC layers formed have a rough structure with ionomer solids that are significantly larger than EC particles. A composite structure of the layer contributes to better water distribution within the fuel cell’s electrodes. The concept was tested using different ECs and directly comparing dispersed vs ground ionomer binder incorporation.^525^ Observed differences in ionomer processing display a trend in EC/ionomer interactions and are critical for high cell performance. The same type of system was tested in PEMFC by Holdcroft and team, incorporating nonconformal hydrocarbon-based ionomer powders into the electrocatalytic layer of the device.^526^ Improved power density performance at high current densities of the fuel cell suggests a different ionic conductivity pathway through the composite layer of the EC with insoluble polymer particles that provides a substantial ionic resistance reduction and mass activity increase. Combined with a substantial porosity of the electrodes caused by the size of insoluble ionomer particle addition, this finding led to a much improved PEMFC performance in the air in comparison to PFAS-based membrane electrode assemblies.^526^ Following the trend, these observations could be translated into the AEM-WE system to further improve it. It presents an interesting question as to the effect of EC layer geometry and morphology on ionomer degradation and the role of nonion-conducting additives as stabilizers in the EC layer. Lindquist et al. showed stable pure water AEM electrolyzer operation for 100 h when using PTFE binder at the anode in the replacement of a conventional ionomer.^462^ While cell voltage was high for commercial applications, advanced electrode designs that use stable binders or additives, but maintain ionic conductivity in the EC layer, may be a viable solution for pure-water AEM operation. Such designs have been deployed as EC protective layers in PEM, enabling stable operation of earth-abundant anode ECs by using a TiO_2_ protective layer to prevent EC corrosion.^527^

**Figure 30:**
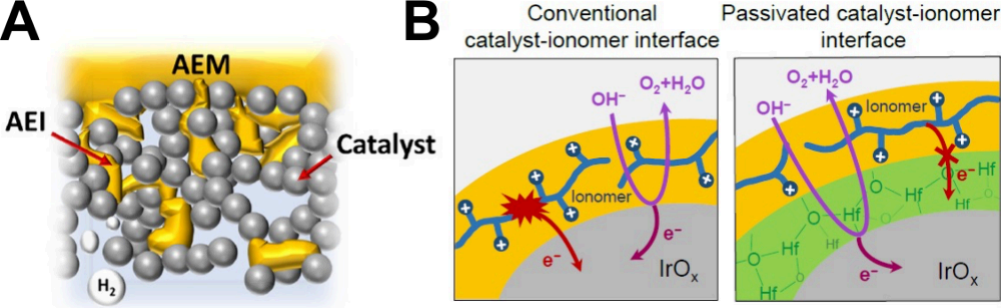
Scheme of EC (EC) layer design approaches: (A) incorporation of solid ionomer particles to facilitate ionic transport within the layer; reproduced from ref 299, copyright 2023, American Chemical Society and (B) incorporation of a passivation layer at the EC-ionomer interface; reproduced with permission from ref 326, copyright 2023, American Chemical Society.

One strategy to stabilize EC layers and associated ionomers may be to engineer the EC surface with passivating inorganic films designed to confine EC dissolution products and prevent deleterious oxidative side reactions due to direct contact between EC and ionomer. To achieve the desired function, the engineered interface passivation layers must be thin and porous enough to allow for the transport of hydroxide ions, reactant and product, while thick enough to retard the direct oxidation of ionomer (Figure 30.B).^326^ If the porosity in such passivation layers can be tuned, it may also be possible for them to allow the transport of small ions, like OH^–^, but prevent the transport of larger ions associated with hydrated dissolved EC cations. Most materials and electrode concepts related to these strategies have only been tested in half cells and not in the AEM-WE context. MEA-level testing with industrial-relevant current density will be required to demonstrate their applicability in the AEM-WE system. For acidic OER, few-nm-thick atomic layer deposition (ALD) TiO_*x*_ coatings on IrO_2_ and RuO_2_ electrodes improved the OER activity and maintained their performance longer than uncoated IrO_2_ or RuO_2_ electrodes.^528^ Co_3_O_4_ anode EC coated with 4.4 nm ALD TiO_2_ showed an increase in OER stability >75 h, compared to uncoated Co_3_O_4_ (25 h) at 10 mA cm^–2^ in 1 M H_2_SO_4_.^527^ It was noted in both works, as well as the recent review^529^ that the thickness of protective coatings is key to understanding the trade-off between the activity and stability of the electrodes. Pt nanoparticles dispersed on carbon fibers were coated with ALD SnO_2_ protective layers to prevent Pt dissolution and detachment and improve the electrode lifetime, which can be used as the cathode for PEMFC.^530^ SiO_*x*_ overlayers have been investigated by Esposito and co-workers, where permselective SiO_*x*_ overlayers on Pt electrodes prevented the undesirable chlorine evolution reaction (CER) while maintaining the desired OER,^531^ as well as the density of SiOxCy overlayers, controlled H^+^ transport.^532^ OH^–^ transport through the thin oxide film should be similar to H^+^ transport, opening up the possibility of the advanced AEM-WE electrode design. The CeO_*x*_ overlayers on Pd and Pt ECs prevented metal dissolution during the hydrogen oxidation reaction (HOR), as assessed by ICP-MS, while the CeO_*x*_ layer appeared to facilitate hydroxide transport, perhaps explaining higher catalytic activity for CeO_*x*_-coated Pd electrodes.^533^ NiFeO_*x*_ ECs for alkaline OER also exhibited improved durability with a 100–200 nm CeO_*x*_ protective layer.^534^ This was explained by the permselectivity of CeO_x_, enabling OH^–^ transport toward NiFeO_*x*_ EC but preventing the diffusion of dissolved Fe species from NiFeO_*x*_ toward electrolyte, maintaining active sites of NiFeO_*x*_. Deploying this concept in AEM-WE might be promising because dynamic Fe dissolution and redeposition throughout the electrolyzer system are thought to be detrimental to the durability of electrolyzers.^427^ Other than focusing on EC degradation, ALD HfO_2_ coatings were used as protective layers at the anodes of AEM-WE to suppress ionomer degradation.^326^ Nanoscale HfO_2_ thin films were shown as OH^–^ conducting but electron-insulating layers at the interface of PiperION ionomer and Ir or CoO_*x*_ anode EC, so that less electron flow from ionomer and EC occurred, which is expected to cause electrochemical oxidation of ionomer.

To design durable AEM-WE electrodes using protective coatings, understanding and controlling the roles of the coatings (i.e., prevention of EC dissolution or detachment, ionomer oxidation, and undesirable side reactions) is required to design the coating chemistry and nanostructure/morphology. Coating materials properties, including ion conductivities (ideally selective for OH^–^), electrical conductivity, and porosity/permeability to electrolyte, are all important. Alkaline and oxidative stability of coatings is needed for stable, insoluble materials during operation. Optimizing the coating parameters of thickness, porosity, and crystallinity may enable improved AEM-WE durability.

### 7.5 Cell Preparation, Assembly, and Operation

Although individual components play a significant role in determining cell performance, preparation and assembly techniques are also crucial. Different coating techniques (e.g., airbrush versus hot-press and coated membranes versus coated electrode) result in different interactions between the EC, ionomer binder, and membrane at the triple-phase boundary, which is the point of simultaneous activity of all the above-mentioned components defining the performance of the electrochemical reaction.^535^ By careful construction of the triple-phase interface, the efficiency and selectivity of the reaction can be significantly improved. Therefore, large performance differences can be observed across MEAs of nominally identical materials.^536^ Using quartz-crystal microbalance (QCM) techniques supported by modeling and isothermal-titration-calorimetry experiments, Weber’s group was able to identify key aspects to describe the relation between ionomer and EC in electrode formation.^537^ The results revealed that water-rich solvents promote ionomer adsorption to the platinum catalytic sites. How the cell is operated before analysis can also affect performance. Cell conditioning is a well-established practice for PEM electrolyzers and fuel cells,^538,539^ during which the cell is operated and performance gradually improves.^540^ Although the detailed processes that cause this improvement are not fully understood, it is hypothesized that cell conditioning further activates EC surfaces, more fully hydrates the membrane and ionomer and better establishes ion pathways through the polymer via chain rearrangement.^538,541,542^

Sometimes different electrolyte feeds are used during conditioning, even if testing in pure water,^57,181,313^ which can also affect measured electrolyzer performance and durability. Conditioning with supporting electrolytes is done due to some advantages over pure-water conditioning. Conditioning in pure water can induce degradation before the initial measurement and therefore yield results with worse performance than the true initial state. The lower operating voltage and consistent supply of OH^–^ to the system during conditioning in supporting hydroxide electrolytes, prevents any cell degradation during conditioning, so the measured performance in pure water is fully pristine. However, intentional care must be taken to ensure all electrolyte is flushed from the system before the pure water measurement.^56,398^

Some observed performance losses in pure water AEM electrolyzer systems are reversible, which some have attributed to CO_2_ gas poisoning of the system.^543^ This is a well-known effect in AEM fuel cells.^477,543^ However, the operating modes of an electrolyzer vs a fuel cell present distinctly different modes in which HCO_3_^–^/CO_3_^2–^ ions enter and interact with the system. In both systems, anions are conducted from the cathode to the anode. Any anions other than OH^–^ present at the cathode will compete with OH^–^ transport in the AEM, decreasing membrane conductivity,^268^ reducing available OH^–^ reactants at the anode,^73^ and increasing local pH at the anode/AEM interface.^73,170^ In AEM fuel cells, CO_2_ and O_2_ enter the cathode, where CO_2_ dissolves in water to form HCO_3_^–^ (in equilibrium with CO_3_^2–^) anions. Impurity effects from dissolved CO_2_ can result in performance losses up to hundreds of mV.^477^ Via modeling, with the increasing CO_2_ concentration at the same operational parameters, a significant reduction in current density was observed by Stanislaw et al.^73^ As the current increases, more HCO_3_^–^/CO_3_^2–^ are introduced and compete with hydroxide. Carbonate ion induces a pH gradient that is responsible for a Nernstian (concentration overpotential) voltage loss.^170^ However, AEM-WEs are ideally operated with liquid water feed to the anode and a dry cathode. In this configuration, any HCO_3_^–^/CO_3_^2–^ present at the anode is blocked from entering the membrane by the direction of the applied potential and strong flux of OH^–^ toward the anode. There is an additional purging effect with increased current density operation, with more OH^–^ produced. At the cathode, any gaseous CO_2_ present is now competing with the kinetically favored HER, and therefore, CO_2_ reduction is likely negligible. Additionally, CO_2_ reduction reaction kinetics are slow at HER potentials. Both systems may still suffer from Nernstian voltage losses due to HCO_3_^–^/CO_3_^2–^ accumulation at the anode/membrane interface due to a pH gradient, but in electrolyzers, this effect is probably negligible relative to losses from ionomer oxidation at the anode. The effect of carbonation is difficult to study at the relevant device operating current in pure water without first addressing the performance-limiting issue of ionomer oxidation.

### 7.6 Accelerated Stability Tests

Stability tests for AEM-WEs are vital to assess the long-term performance of these devices. At the same time, AEM-WE’s durability tests refer to the ability of the device to withstand various operating conditions inflicted by the accelerated stress tests (ASTs). These evaluation techniques provide insights into possible degradation mechanisms and enable the optimization of the system’s components and overall cell design.

To date, there are no established and widely recognized procedures for the accelerated stability testing of AEM-WEs. Due to the relative newness of the technology, a lot of protocols could be translated from more mature technologies of PEM-WE or A-WE, where most test specifications are agreed upon and follow a common goal of improving water electrolysis technology for industrial demands.^544^

The rapid development of new components of electrolyzers with a prospective commercialization plan calls for a reliable performance that can ensure at least 50,000 h of operational time to be an industry-viable product. Therefore, there is a need for accelerated stress protocol development that could be well understood and translated into an extended longevity performance. ASTs for water electrolyzers typically focus on the chemical, mechanical, and thermal degradation aspects. Most of these could be addressed via in situ applied high current density, dynamic operation of the cell and shutdown models.^545^ For an AST, it is crucial to understand the degradation mechanism of materials and components used in the electrolyzer. Understanding the performance under those conditions allows the investigation of the accelerated aging process as well as the operation of the system after a failure during a real-life operation caused by external factors (ex., power supply shutdown). However, the limitation caused by the inability to perfectly mimic the operating conditions of the device suggests that tests performed ex-situ are not always translatable to the real-life system. It is of utmost importance for the AST to reflect relevant AEM-WE operating conditions, for example, performing the testing on the MEA configuration in the enclosed cell vs aqueous model system (three electrode setup) experiment, where some of the factors cannot be accounted for.^68^

*Ex-situ* AST testing protocols and descriptions could be found in previous chapters of the manuscript; however, it is important to discuss findings following the in situ exposure to accelerated degradation factors. These factors should mostly focus on the actual operating strategy of a commercial device and adjust to the timeframes of such, for example, considering the surplus of renewable energy.^546^ Since AST must guarantee meeting the expectations of durability for the commercial systems, the development of such a protocol should include information on performance and efficiency. Developed in 2020 by Aßmann et al. AST protocols for commercial PEM water electrolyzers involve a four-step procedure described further in the paragraph (Figure 31).^545^ The testing should include a nonintentionally accelerated condition at a nominal current density that still would induce some degradation over time. The next component of such testing is a high current density operation or overload, the impact of which will influence the ionomer, membrane, and anode EC layer, as well as PTL if exposed. Further load cycling, which applies to hydrogen production from fluctuating renewable sources, will affect the cathode EC layer, causing its premature degradation. As a part of sudden power loss testing, open circuit operation or a complete shut-down process will mostly affect the cathode components. Similar to this development, the AST protocol for AEM-WE must be standardized, including in-between CV, EIS and steady-state polarization curves to monitor the state of health of the device.

**Figure 31:**
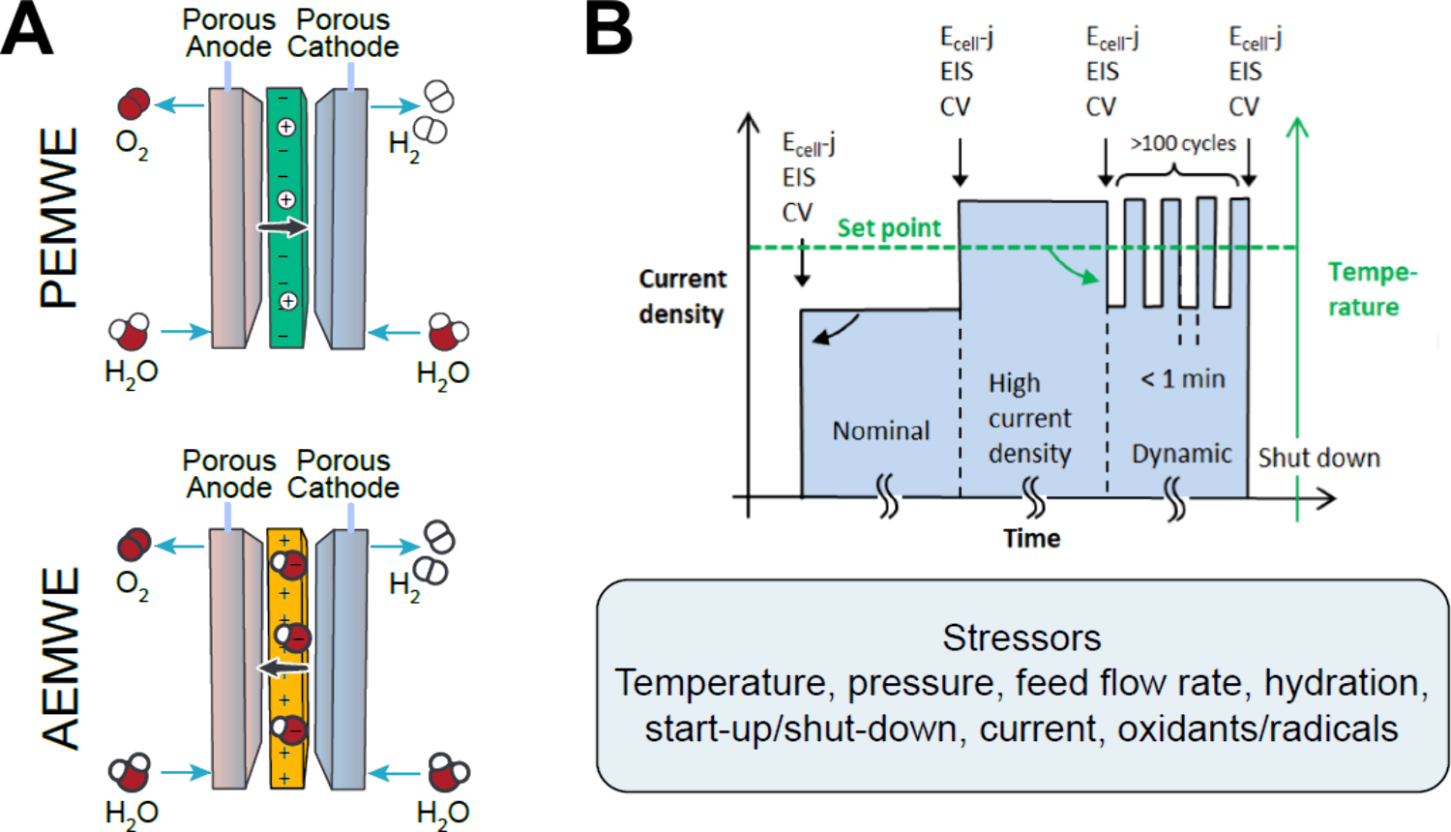
Suggested harmonized protocol for accelerated stability testing of water electrolyzers. (A) MEA schematic of PEM-WE and AEM-WE with corresponding stressors of systems. (B) Testing protocol scheme reproduced with permission from ref 545. Copyright 2020, Elsevier B.V.

The high chemical and mechanical durability of the AEM-WE’s components under a dynamic start–stop operation is a direct pathway to reliable electrochemical energy conversion devices that function complementary with intermittent renewable power sources. An extended single-cell durability study performed by Narayanaru et al. (2023) in 1 M KOH feed at 80 °C evaluates 1000 start–stop voltage cycles between 0.1 and 2.0 V.^547^ In this work, the discussion is focused on the durability of MEA, which is affected by the ionomer-binder degradation issue described in previous chapters, as well as the mechanical strength of AEM in an alkaline environment challenged by the rapid bubble formation, causing nonlinear stress and shutdown. The results suggest no obvious deterioration; however, a nominal increase in the cell voltage shows an additional 9 mV requirement to reach the same current density after 1000 cycles. Impedance spectra collected after the test indicate no changes to the resistance of the polymer electrolyte membrane and, as well as no solid EC leached out of the system. Despite these findings, when speaking about the AEM-WE with alkaline electrolyte-feed, one needs to consider a nuisance typically observed in alkaline water electrolyzers concerning EC decay caused by frequently occurring potential change (emergency shutdown/unstable supply of renewable energy) associated with reverse polarization.^548^ The generation of degrading reverse current in liquid alkaline systems is attributed to a significant difference between the discharge capacity of the electrodes on the same BP. Therefore, if a cathode EC on one side of a BP has low redox durability, the anode discharge capacity must be controlled to limit the generation and the effect of reverse current. One of the approaches is to manage the surface area of the electrode and work on the various metal cations EC with suitable activity to improve the design of the anode.

## 8 Conclusions

The push for sustainable energy solutions and low-emission hydrogen as an energy vector has intensified research on electrolysis and, lately, on AEM-WE to increase supply chain resilience by avoiding the usage of critical resources. So far, most of the AEM-WE investigations have been devoted to KOH-containing electrolytes. A significant interest exists in transitioning to KOH-containing electrolytes to pure water feeds to cut down the cost and limit the degradation of the electrolyzer component. Given that pure water is limited in nature, its use for AEM-WE raises environmental and logistical issues. Only 3% of earth’s water is freshwater, supporting various human activities like agriculture and industry, and this supply is under growing human pressure.

Historically, doubts existed about the viability of a hydrogen economy, primarily due to concerns about water use. Yet, progress over recent years has enhanced water electrolysis efficiency, paving the way for eco-friendly hydrogen production with minimal environmental burden. Data indicates that although a hydrogen-based economy would need vast amounts of water, its actual consumption and ensuing environmental footprint are relatively low, especially when compared to sectors like agriculture and energy production from fossil fuels.

A significant breakthrough would be adjusting AEM-WE to work with less refined water or even seawater. Such an approach would diversify water sources and decrease freshwater demand. The evolution of electrolysis technologies has allowed for the utilization of impure water sources, such as sea or tap water, to economically produce hydrogen, with a significant focus on AEM-WE. PEM-WE systems face distinct challenges when operating with these impure water sources. The primary issue stems from potential contaminants that can degrade the electrolyzer’s critical components and increase maintenance costs.

A notable challenge in this context is the chloride ions present in impure water, which can cause extensive damage to cell components. When the environment becomes acidic, these ions participate in reactions that produce harmful byproducts. However, AEM-WE systems, due to their operation at a higher pH, present a certain degree of resistance against these negative effects of chloride ions. Despite this advantage, challenges persist. The degradation of ECs by elements in impure water can significantly reduce the efficiency of the electrolysis process.

Recent technological advancements offer potential solutions. One such innovation is the asymmetric configuration of electrolyzers, which shows promise in mitigating some of these challenges. However, the issue of chloride-induced corrosion remains a concern. To address this, specific anionic additives have been explored, which serve to protect against such corrosion, enhancing the lifespan of system components.

Further research has also focused on methods to deter harmful ions, effectively using anions to improve electrode stability. A notable method integrates seawater electrolysis with hydrazine degradation. This approach not only achieves more energy-efficient hydrogen production but also reduces the corrosive impacts typically associated with chloride ions. Such advancements highlight the significance of combining chemical knowledge with engineering solutions to progress toward sustainable energy outcomes and a reduction in carbon emissions. However, the use of highly impure water sources remains a major challenge with no current short-term solutions. Accordingly, Water purification for AEM-WE remains vital.

Current methods to purify water may include thermal treatments, membrane distillation, and RO. Among these, RO stands out due to its energy and cost efficiency. There’s also a trend toward combining multiple purification techniques to boost efficiency and reduce expenses. Remarkably, the current energy cost for the RO purification of water per kilogram of hydrogen is negligible (0.3 kWh kg^–1^) and somewhat acceptable for final technologies. Indeed, RO may allow the use of seawater that is extremely abundant, avoiding an impact on freshwater.

Transitioning to systems using pure water in AEM-WE is challenging because of the high resistance of the medium. Efficient ion transport between the anode and cathode is crucial for the electrocatalysis of hydrogen and oxygen. This is seamlessly handled in polymer electrolyte membrane electrolyzers using ultrapure water, thanks to membranes that facilitate efficient H^+^ transport. Yet, replicating this in AEM-WE is tough because OH^–^ ions inherently move three times slower than protons.

This reveals that the ion-conducting components—membranes and ionomers—are vital. Growing interest in AEMs, especially within AEM-WEs, hinges on their role as separators and facilitators of selective ion transport. Their effectiveness is contingent upon improving their ionic conductivity and chemical stability.

A key distinction arises when comparing operating media: alkaline solutions vs pure water. While alkaline solutions offer better conductivity and reaction rates, they pose corrosion and safety challenges. Conversely, pure water reduces these concerns but, without alkaline substances, results in higher hydroxide ion concentrations, putting membrane durability at risk.

Ionomers, whether part of the membrane or separate, can adjust ionic conductivities and might lessen high hydroxide concentrations adverse effects in pure water settings. Incorporating them thoughtfully in the MEAs can combine high conductivity with increased chemical stability.

For optimal utilization of pure water, it is crucial to redefine membrane and ionomer characteristics. Modifying the IEC can help balance conductivity and lifespan. Using advanced analytical methods can provide a better understanding of these conditions, leading to the development of superior membranes. As the pursuit of an electrolyte-free AEM-WE continues, understanding the intricate relationships between alkaline solutions, pure water, and ionomer components is critical, guiding and, ultimately, the lighthouse that has to drive the research.

According to the findings reported in this review, high current density operations have already been achieved and we believe that pure water-fed AEM-WE is feasible. The transition to pure water might simplify the electrolyzer design and operations, limiting the use of harmful alkalis and relying on abundant and inexpensive water purification technology for the upstream. To this respect, the major research challenges include the development of robust and conductive alkaline membranes and ionomers and tailored processes to integrate them with the ECs in MEAs.
